# Supplementary material for: OCIAD1 and prohibitins regulate the stability of the TIM23 protein translocase
Source: Cell Rep. 2024 Dec 3;43(12):115038. doi: 10.1016/j.celrep.2024.115038 (PMC11672691; doi:10.1016/j.celrep.2024.115038)
Supplement: Document S1. Figures S1–S10 [file mmc1.pdf]

Cell Reports, Volume *43*

Supplemental information

OCIAD1 and prohibitins regulate the stability of the TIM23 protein translocase

Praveenraj Elancheliyan, Klaudia K. Maruszczak, Remigiusz Adam Serwa, Till Stephan, Ahmet Sadik Gulgec, Mayra A. Borrero-Landazabal, Sonia Ngati, Aleksandra Gosk, Stefan Jakobs, Michal Wasilewski, and Agnieszka Chacinska

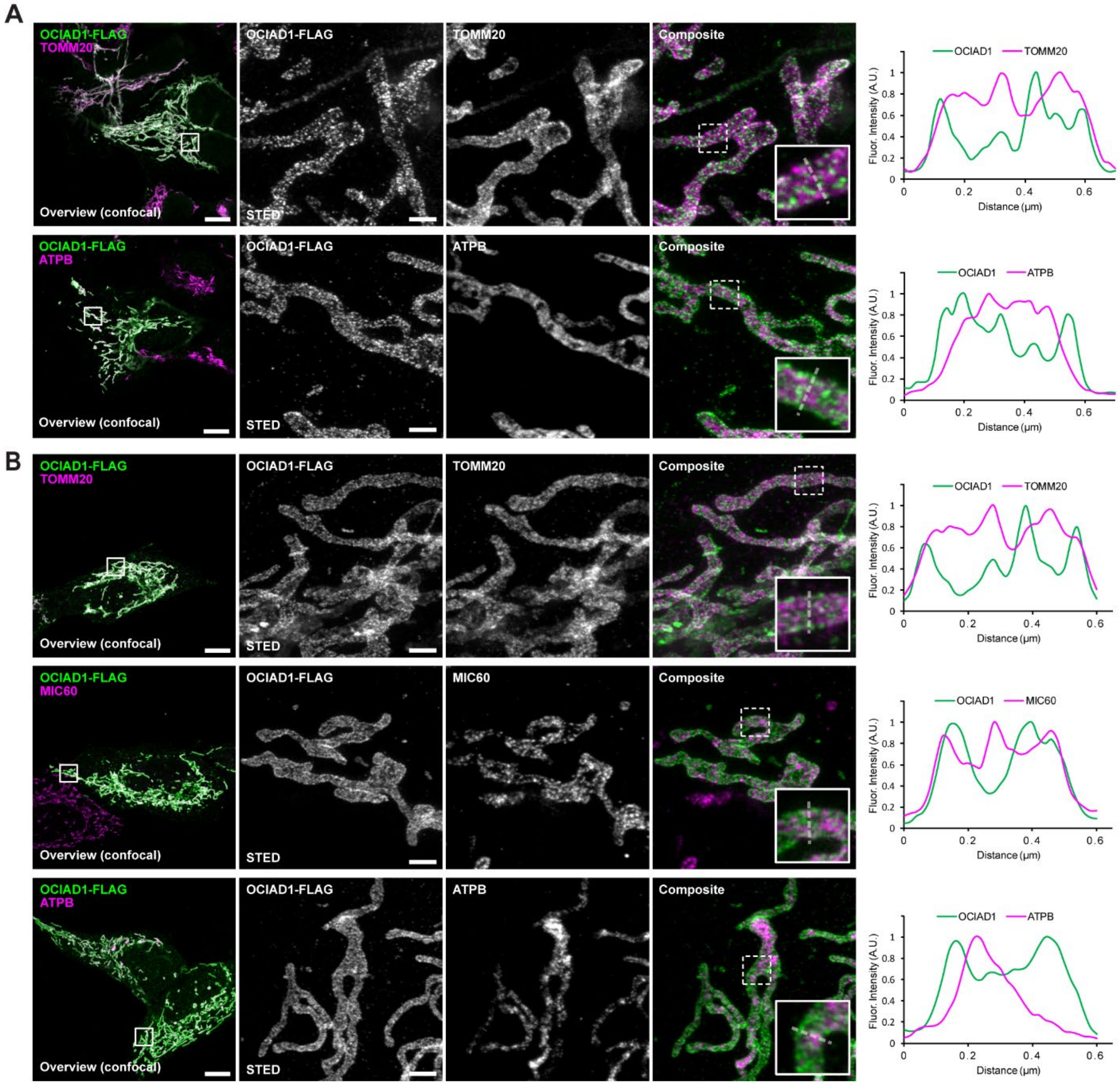

Figure S1. Submitochondrial distribution of OCIAD1-FLAG, related to Figure 1 (A, B) OCIAD1-FLAG submitochondrial localization, visualized by confocal and 2D STED microscopy. Cells were transfected with a plasmid coding OCIAD1-FLAG, fixed and immunolabeled against the FLAG-epitope and different mitochondrial proteins. Samples were recorded by 2-color 2D STED nanoscopy. Fluorescence intensity line profiles were measured along the dashed lines shown in the insets. Fluorescence intensities were normalized and plotted across the mitochondrial tubules. Line profiles indicate the intramitochondrial position of the individual protein clusters. (A) HEK 293 cells were labeled against the FLAG epitope and TOMM20 (outer membrane) or ATPB (cristae membrane), respectively. (B) U-2 OS cells were labeled against the FLAG epitope and

TOMM20 (outer membrane), MIC60 (inner boundary membrane/crista junctions), or ATPB (cristae membrane), respectively. ATPB signals are generally shifted towards the center of the mitochondrial tubules. Scale bars: 10 µm (overview), 1 µm (STED).

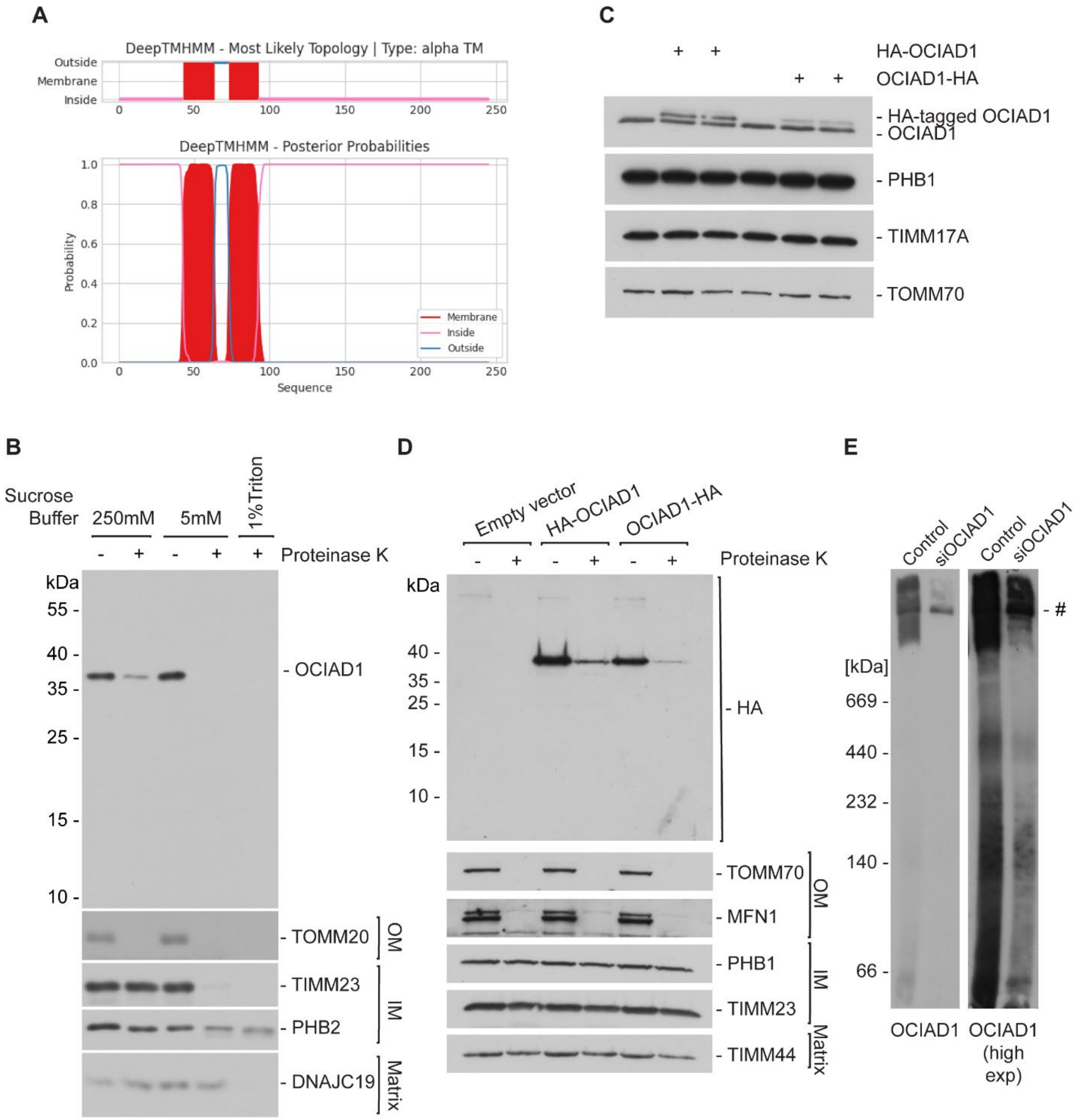

Figure S2. OCIAD1 is a transmembrane protein of the outer mitochondrial membrane and exposes both the N- and C-terminus to the cytosol, related to Figure 1 (A) DeepTMHMM prediction of OCIAD1 transmembrane domains. (B) Localization of mitochondrial proteins, analyzed by limited degradation by proteinase K in intact mitochondria (250 mM sucrose), mitoplasts (5 mM sucrose), and mitochondrial lysates (1% Triton X-100). The samples were analyzed by SDS-PAGE and Western blot. OM, outer membrane; IM, inner membrane. (C) Expression of HA-OCIAD1 and OCIAD1-HA in HEK293T cells. The samples were analyzed by

SDS-PAGE and Western blot. (D) Localization of mitochondrial proteins isolated from HEK293T cells expressing HA-OCIAD1 and OCIAD1-HA, analyzed by limited degradation by proteinase K in intact mitochondria. The samples were analyzed by SDS-PAGE and Western blot. OM, outer membrane; IM, inner membrane. (E) Analysis of OCIAD1 in mitochondria isolated HEK293 cells transfected with scramble (Control) or siRNA against OCIAD1 (siOCIAD1). The samples were analyzed by BN-PAGE and Western blot. #, unspecific band.

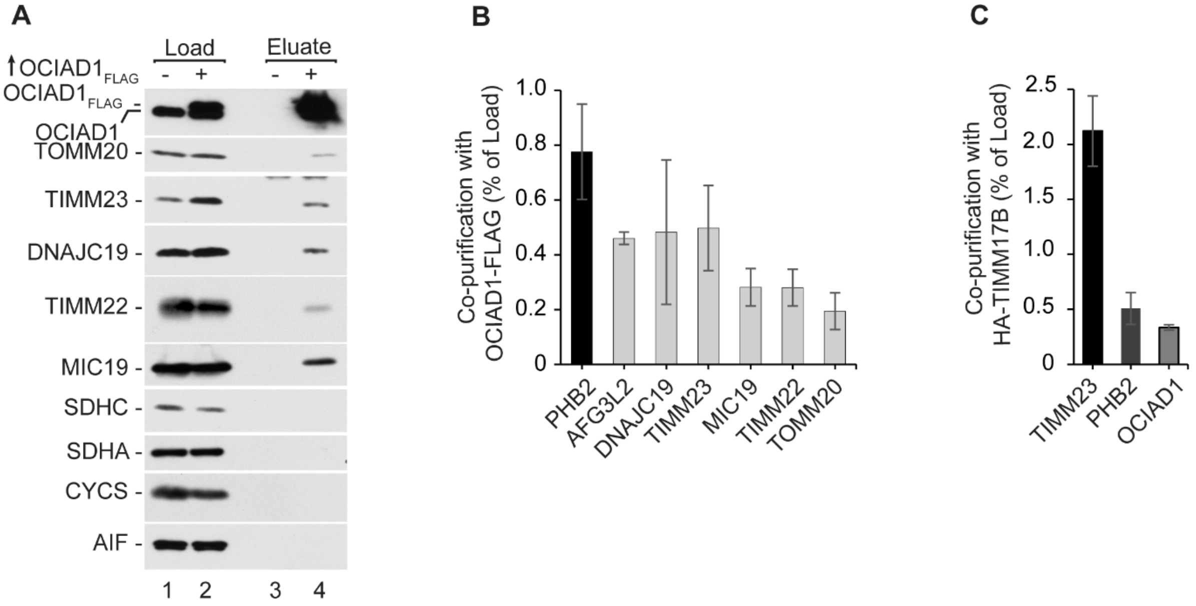

Figure S3. OCIAD1 interacts with the prohibitin complex and the TIM23 complex, related to Figure 2 (A) Affinity purification of $OCIAD1_{FLAG}$. Mitochondria were isolated from HEK293 cells that expressed $OCIAD1_{FLAG}$ or an empty vector, solubilized in digitonin-containing buffer, and subjected to immunoprecipitation with FLAG affinity resin. Samples were analyzed by SDS-PAGE and Western blot. Load: 2 %; Eluate: 100 %. (B) Quantification of protein co-purification with $OCIAD1_{FLAG}$ presented as a ratio of eluate to load (means ± SEM, n = 3). (C) Quantification of protein co-purification with HA-TIMM17B presented as a ratio of eluate to load (means ± SEM, n = 3).

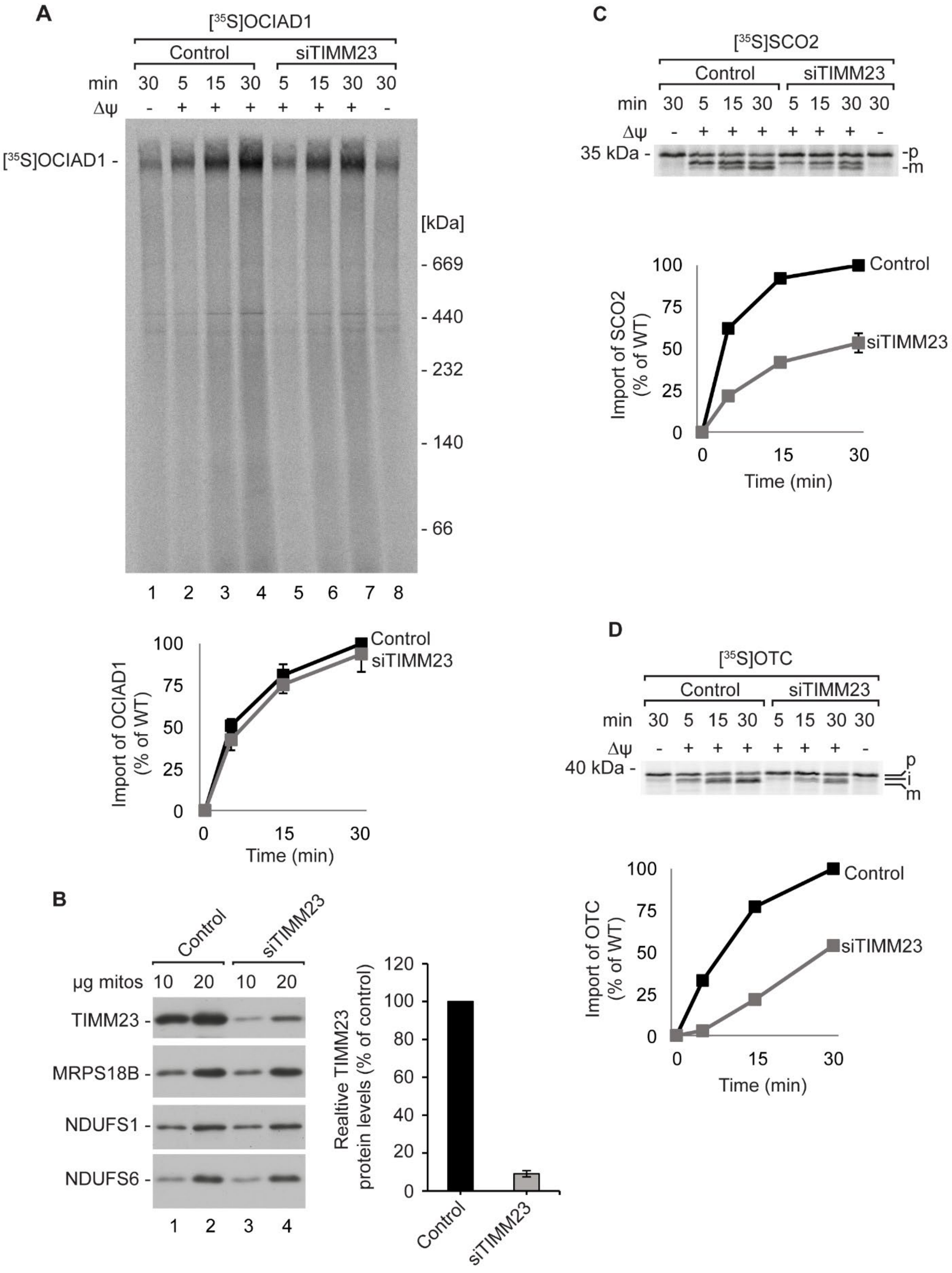
A
[35S]OCIAD1
Control
siTIMM23
min 30 5 15 30 5 15 30 30
Δψ - + + + + + + -
[35S]OCIAD1 -
[kDa]
- 669
- 440
- 232
- 140
- 66
1 2 3 4 5 6 7 8
Import of OCIAD1 (% of WT)
100
75
50
25
0
0 15 30
Time (min)
Control
siTIMM23
B
Control
siTIMM23
µg mitos 10 20 10 20
TIMM23 -
MRPS18B -
NDUFS1 -
NDUFS6 -
1 2 3 4
Realtive TIMM23 protein levels (% of control)
120
100
80
60
40
20
0
Control
siTIMM23
C
[35S]SCO2
Control
siTIMM23
min 30 5 15 30 5 15 30 30
Δψ - + + + + + + -
35 kDa -
-p
-m
Import of SCO2 (% of WT)
100
75
50
25
0
0 15 30
Time (min)
Control
siTIMM23
D
[35S]OTC
Control
siTIMM23
min 30 5 15 30 5 15 30 30
Δψ - + + + + + + -
40 kDa -
p
i
m
Import of OTC (% of WT)
100
75
50
25
0
0 15 30
Time (min)
Control
siTIMM23

Figure S4. OCIAD1 is not imported by the TIM23 pathway, related to Figure 2 (A) *In organello* import of OCIAD1. Radiolabeled $^{[35S]}$OCIAD1 precursors were imported into the mitochondria of siRNA TIMM23-depleted and control HEK293 cells. Mitochondria were analyzed with 3-13 % BN-PAGE gels and autoradiography. Control cells were transfected with Mission siRNA universal negative control. Where indicated the mitochondrial membrane electrochemical potential ($\Delta\Psi$) was dissipated by valinomycin, antimycin A, and oligomycin. The quantification of autoradiography signals is presented as the mean ± SEM (n = 3). The amount of imported protein into control mitochondria after 30 min was set to 100 %. (B) Western blot analysis of mitochondria isolated from control and TIMM23-depleted cells. The quantification of TIMM23 from lanes that were loaded with 10 µg mitochondria is presented as the mean ± SEM (n = 3). Control, cells transfected with Mission siRNA universal negative control. (C, D) *In organello* import of OCIAD1. Radiolabeled $^{[35S]}$SCO2 (C) or $^{[35S]}$OTC (D) were imported into the mitochondria of TIMM23-depleted and control HEK293 cells. Mitochondria were analyzed with SDS-PAGE gels and autoradiography. Control cells were transfected with Mission siRNA universal negative control. Where indicated the mitochondrial membrane electrochemical potential ($\Delta\Psi$) was dissipated by valinomycin, antimycin A, and oligomycin. The quantification of autoradiography signals is presented as the mean ± SEM (n = 3). The amount of imported protein into control mitochondria after 30 min was set to 100 %. p, precursor form; i, intermediate form; m, mature form.

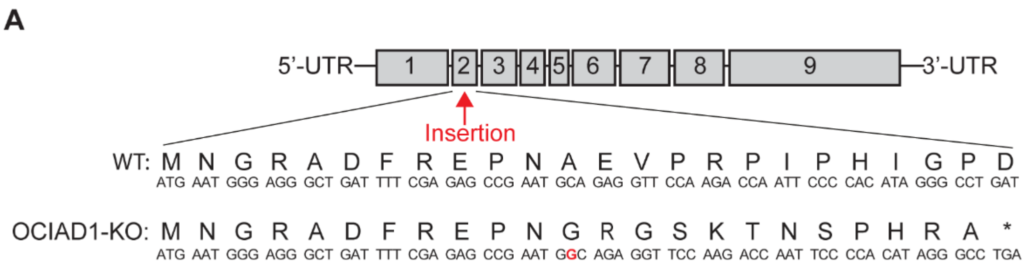

Figure S5. Schematic representation of mutation of the *OCIAD1* gene in OCIAD1-KO cells, related to Figure 3 (A) The OCIAD1-KO cell line was generated using CRISPR/Cas9 technology by the homozygous insertion of guanosine (G) in the second intron of *OCIAD1*, leading to a frameshift in the ORF and a premature stop codon.

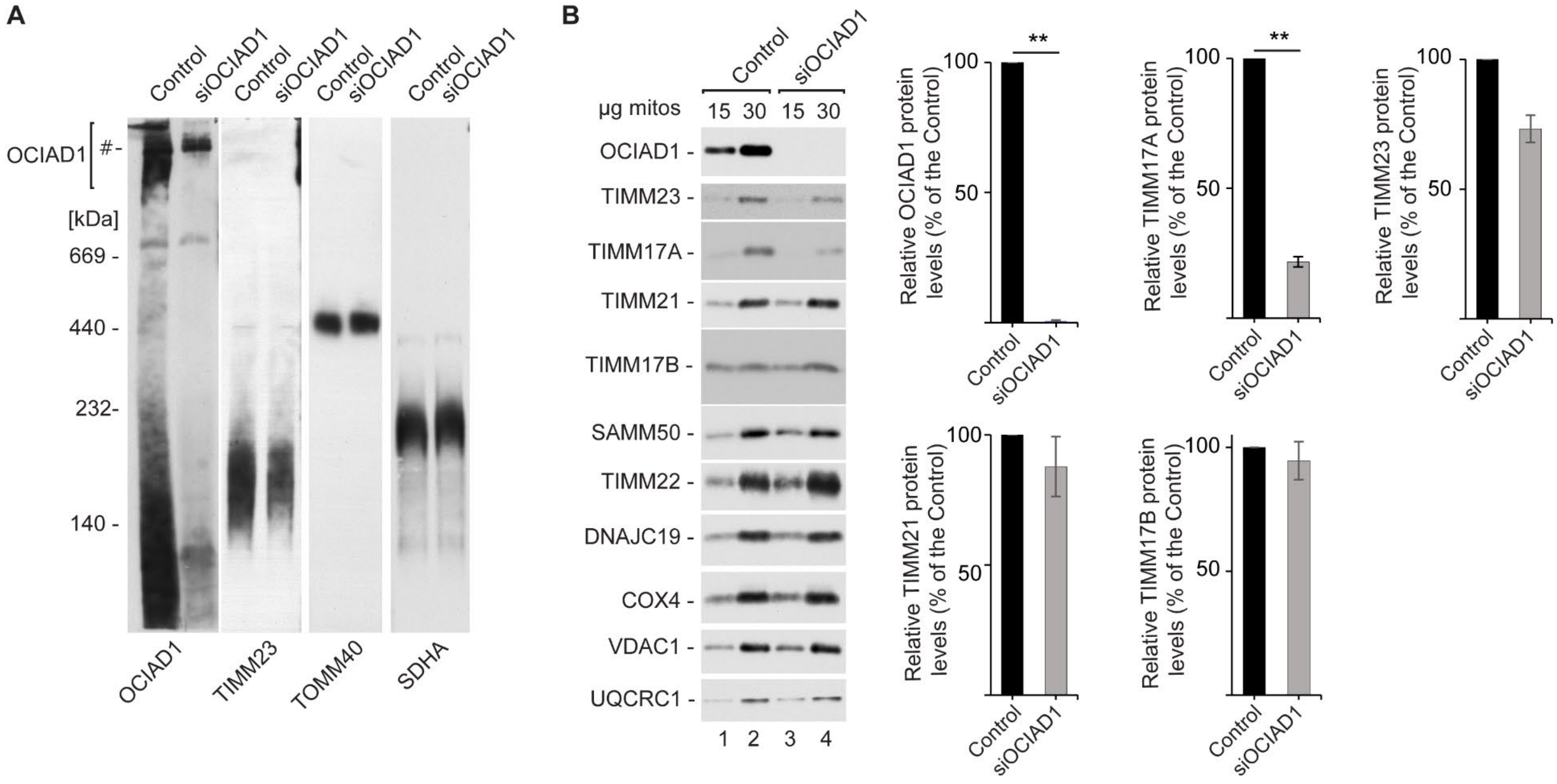
A
Control
siOCIAD1
Control
siOCIAD1
Control
siOCIAD1
Control
siOCIAD1
OCIAD1
#-
[kDa]
669 -
440 -
232-
140 -
OCIAD1
TIMM23
TOMM40
SDHA
B
Control
siOCIAD1
µg mitos
15 30 15 30
OCIAD1 -
TIMM23 -
TIMM17A -
TIMM21 -
TIMM17B -
SAMM50 -
TIMM22 -
DNAJC19 -
COX4 -
VDAC1 -
UQCRC1 -
1 2 3 4
Relative OCIAD1 protein levels (% of the Control)
Relative TIMM17A protein levels (% of the Control)
Relative TIMM23 protein levels (% of the Control)
Relative TIMM21 protein levels (% of the Control)
Relative TIMM17B protein levels (% of the Control)
100
50
**
Control
siOCIAD1

Figure S6. OCIAD1 depletion downregulates the TIM23 complex, related to Figure 5 (A) Protein complexes in mitochondria that were isolated from HEK293 cells with native or siRNA-depleted OCIAD1, solubilized with 1% digitonin-containing buffer, and resolved in 4-13 % BN-PAGE gels, followed by Western blot. Control, cells transfected with Mission siRNA universal negative control; #, unspecific band. (B) Western blot analysis of mitochondria that were isolated from HEK293 cells with native and siRNA-depleted OCIAD1. The quantification of protein signals from lanes that were loaded with 30 µg mitochondria is presented as the mean ± SEM (n = 3). *$p < 0.05$, **$p < 0.01$ (two-tailed Student's t-test). mitos, mitochondria

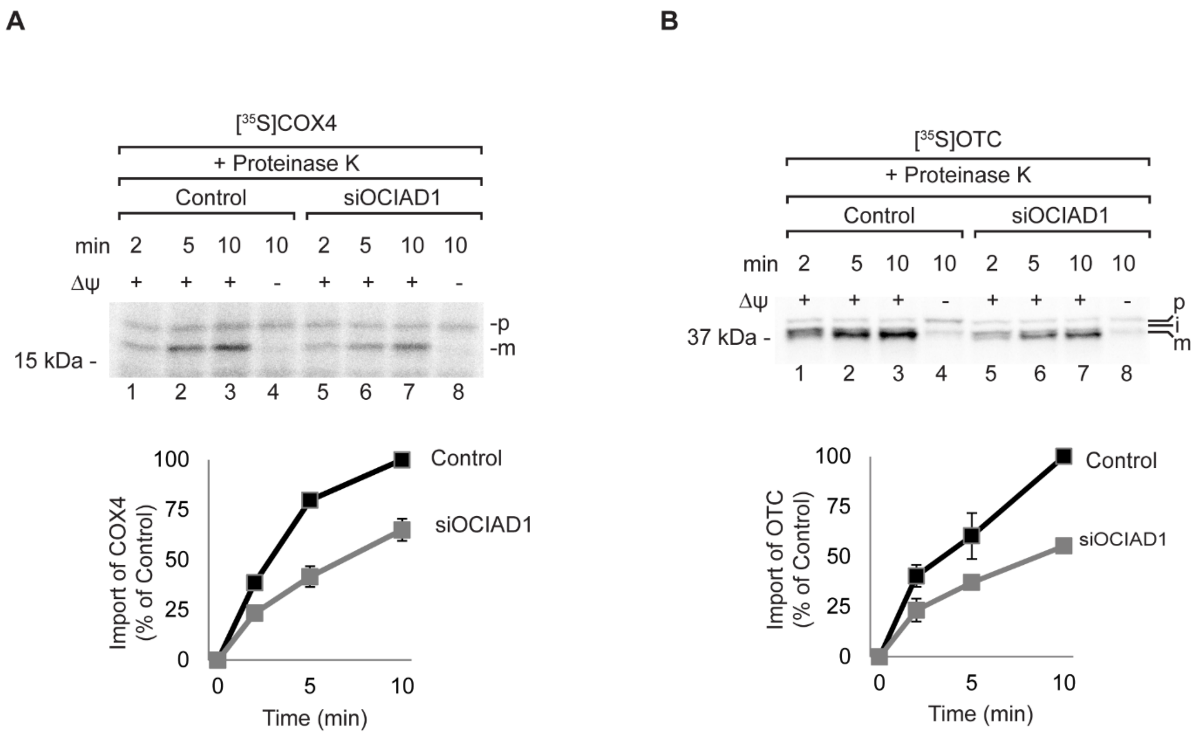

Figure S7. The absence of OCIAD1 downregulates the efficiency of the TIM23 import pathway, related to Figure 5 (A, B) *In organello* import of [^{35}S]COX4 (A) and [^{35}S]OTC (B) precursors into mitochondria that were isolated from HEK293 cells with native or siRNA-depleted OCIAD1, analyzed by SDS-PAGE and autoradiography. The quantification of autoradiography signals is presented as the mean ± SEM (*n* = 3). The amount of imported protein into wild-type mitochondria after 10 min was set to 100 %. Control, cells transfected with Mission siRNA universal negative control. VOA, valinomycin, antimycin A, and oligomycin. p, precursor form; i, intermediate form; m, mature form.

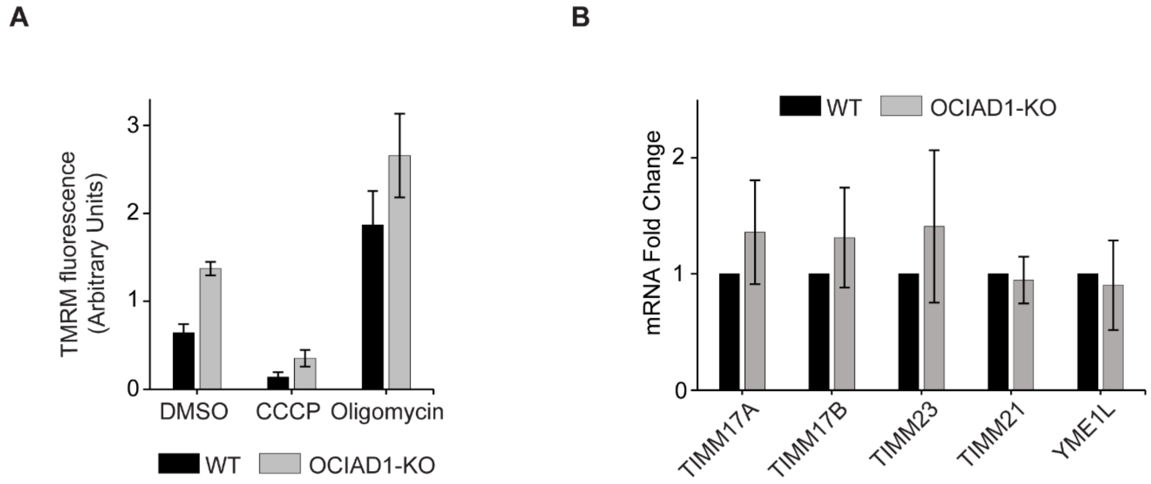

Figure S8. The absence of OCIAD1 does not influence the mitochondrial membrane electrochemical potential or mRNA levels of TIM23 subunits, related to Figure 5 (A) Mitochondrial membrane electrochemical potential in wild-type and OCIAD1-KO HEK293 cells treated with CCCP (10 μM) or oligomycin (0.6 μM) for 30 min, analyzed by TMRM staining, followed by flow cytometry. The quantification of TMRM fluorescence is presented as the mean ± SEM (*n* = 3). CCCP, carbonyl cyanide m-chlorophenyl hydrazone; TMRM, tetramethylrhodamine methyl ester. (B) mRNA expression patterns of selected transcripts, analyzed by RT-qPCR in wild-type and OCIAD1-KO cells. mRNA levels are presented as fold changes relative to wild-type HEK293 cells (mean ± SD, *n* = 3). WT, wild-type.

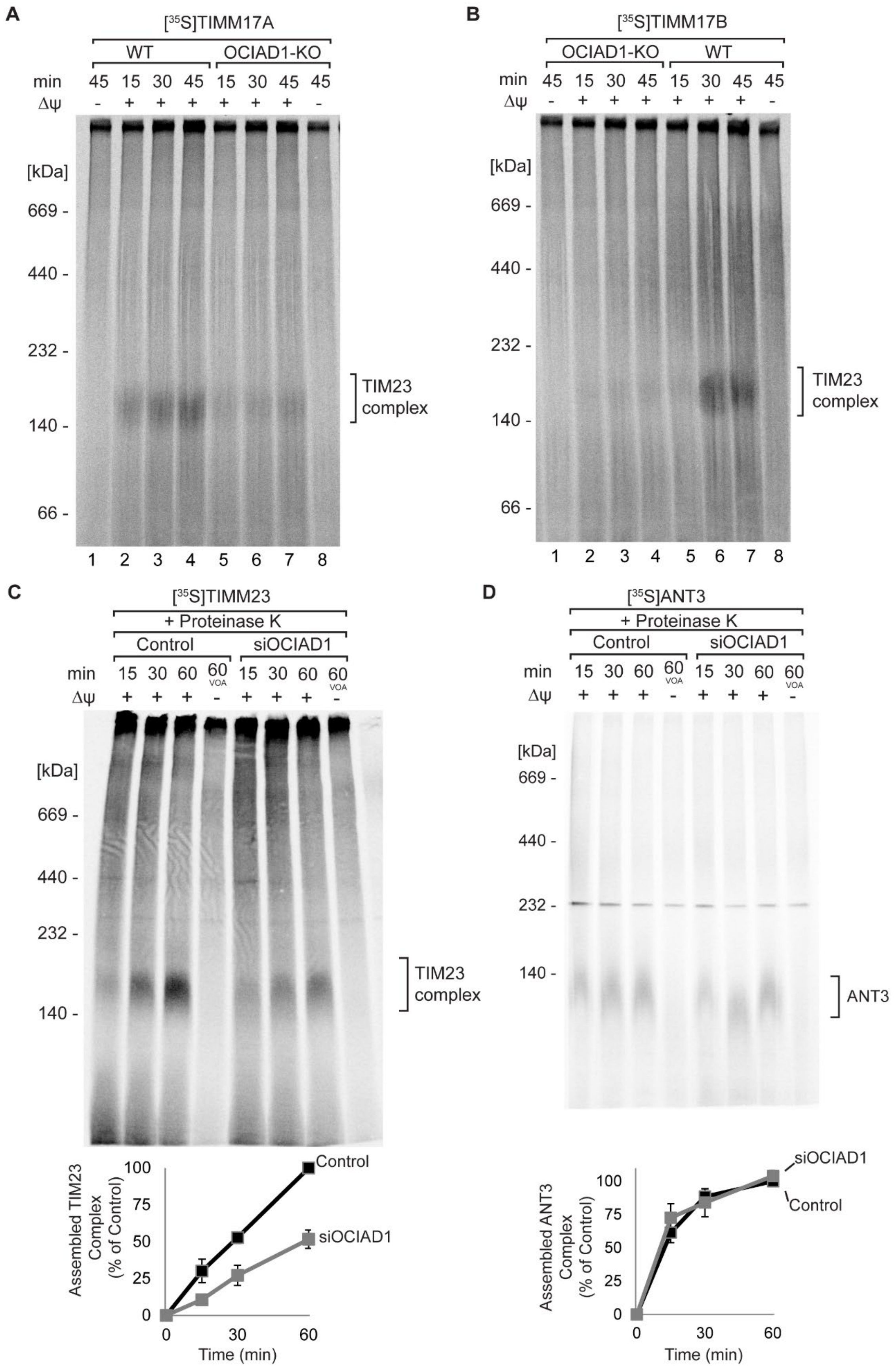

A
[35S]TIMM17A
WT
OCIAD1-KO
min 45 15 30 45 15 30 45 45
Δψ - + + + + + + -
[kDa]
669 -
440 -
232 -
140 -
66 -
TIM23 complex
1 2 3 4 5 6 7 8
B
[35S]TIMM17B
OCIAD1-KO
WT
min 45 15 30 45 15 30 45 45
Δψ - + + + + + + -
[kDa]
669 -
440 -
232 -
140 -
66 -
TIM23 complex
1 2 3 4 5 6 7 8
C
[35S]TIMM23
+ Proteinase K
Control
siOCIAD1
min 15 30 60 60 VOA 15 30 60 60 VOA
Δψ + + + - + + + -
[kDa]
669 -
440 -
232 -
140 -
TIM23 complex
Assembled TIM23 Complex (% of Control)
Time (min)
Control
siOCIAD1
D
[35S]ANT3
+ Proteinase K
Control
siOCIAD1
min 15 30 60 60 VOA 15 30 60 60 VOA
Δψ + + + - + + + -
[kDa]
669 -
440 -
232 -
140 -
ANT3
Assembled ANT3 Complex (% of Control)
Time (min)
siOCIAD1
Control

Figure S9. OCIAD1 depletion decreases the assembly of the TIM23 complex, related to Figure 6 (A, D) *In organello* import of $^{[35S]}$TIMM17A (A) and $^{[35S]}$TIMM17B (B) precursors into mitochondria that were isolated from the wild-type (WT) or OCIAD1-KO HEK293 cells, analyzed by 3-13 % BN-PAGE gels and autoradiography. Where indicated the mitochondrial membrane electrochemical potential (ΔΨ) was dissipated by VOA, valinomycin, antimycin A, and oligomycin. (C, D) *In organello* import of $^{[35S]}$TIMM23 (A) and $^{[35S]}$ANT3 (B) precursors into mitochondria that were isolated from HEK293 cells with native or siRNA-depleted OCIAD1, analyzed by 4-13 % BN-PAGE gels and autoradiography. Where indicated the mitochondrial membrane electrochemical potential (ΔΨ) was dissipated by VOA, valinomycin, antimycin A, and oligomycin. The quantification of autoradiography signals is presented as mean ± SEM (n = 3). The amount of imported protein into wild-type mitochondria at 60 min was set to 100 %. Control, cells transfected with Mission siRNA universal negative control.

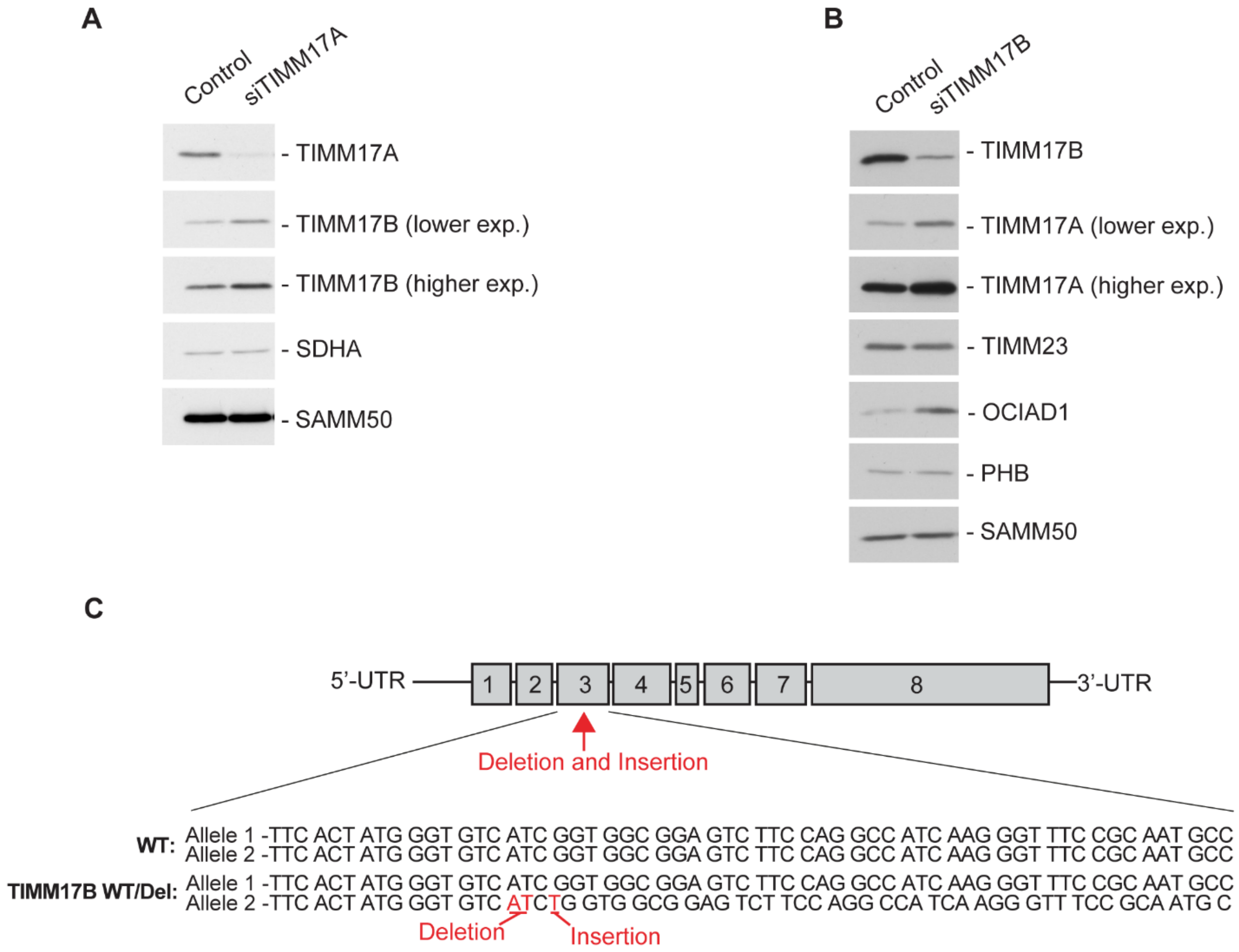

Figure S10. Compensatory regulation of TIMM17A and TIMM17B proteins. Schematic representation of mutations of the *TIMM17B* gene in TIMM17B WT/Del HEK293 cells, related to Figure 7 (A) Western blot analysis of mitochondria isolated from control and siRNA-depleted TIMM17A cells. Control, cells transfected with Mission siRNA universal negative control. (B) Western blot analysis of mitochondria isolated from control and siRNA-depleted TIMM17B cells. Control, cells transfected with Mission siRNA universal negative control. (C) The TIMM17B WT/Del cell line was generated using CRISPR/Cas9 technology by heterozygous deletion and insertion in the third intron of *TIMM17B*, leading to a frameshift in the ORF and a premature stop codon. WT, wild-type.
