# Supplementary material for: OCIAD1 and prohibitins regulate the stability of the TIM23 protein translocase
Source: Cell Rep. 2024 Dec 3;43(12):115038. doi: 10.1016/j.celrep.2024.115038 (PMC11672691; doi:10.1016/j.celrep.2024.115038)
Supplement: Document S2. Article plus supplemental information [file mmc3.pdf]

Cell Reports

Article

# OCIAD1 and prohibitins regulate the stability of the TIM23 protein translocase

## Graphical abstract

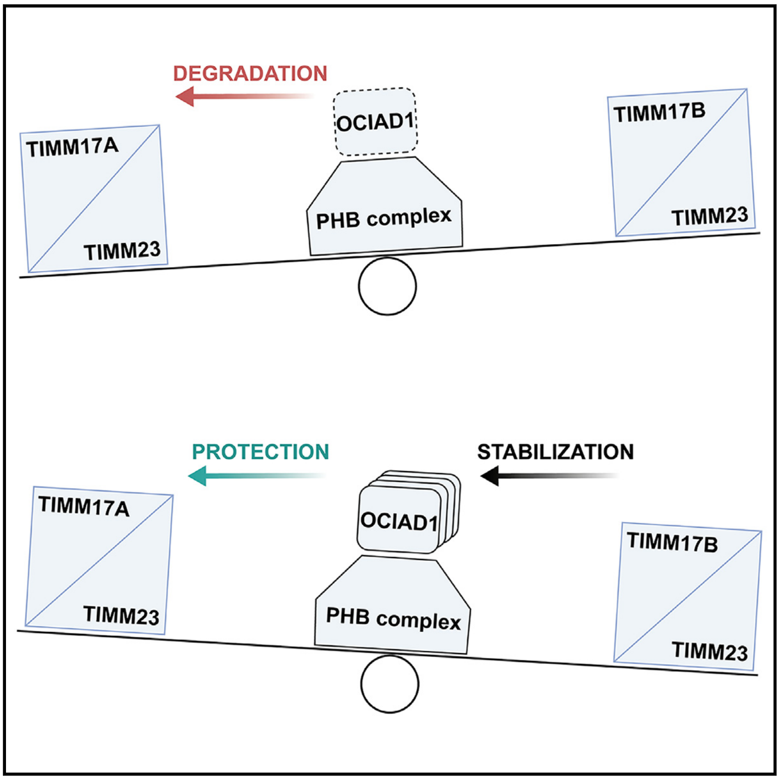


## Highlights

- The PHB complex controls both TIMM17A- and TIMM17B-containing TIM23 translocases
- Depletion of OCIAD1 reduces the levels of TIMM17A- but not TIMM17B-containing TIM23
- Absence of TIMM17B-containing TIM23 positively regulates OCIAD1 abundance
- OCIAD1 and prohibitins constitute a regulatory axis of the human TIM23 complex

## Authors

Praveenraj Elancheliyan, Klaudia K. Maruszczak, Remigiusz Adam Serwa, ..., Stefan Jakobs, Michal Wasilewski, Agnieszka Chacinska

## Correspondence

m.wasilewski@imol.institute (M.W.), a.chacinska@imol.institute (A.C.)

## In brief

Elancheliyan et al. demonstrate that the prohibitin complex is critical for biogenesis of the TIMM17A- and TIMM17B-containing TIM23 translocases. A regulatory protein, OCIAD1, differently controls the levels of both translocase variants. The authors propose a regulatory axis that controls the levels of the TIM23 complex in human cells.

Elancheliyan et al., 2024, Cell Reports 43, 115038
December 24, 2024 © 2024 The Authors. Published by Elsevier Inc.
https://doi.org/10.1016/j.celrep.2024.115038

CellPress

Cell Reports

CellPress
OPEN ACCESS

Article

# OCIAD1 and prohibitins regulate the stability of the TIM23 protein translocase

Praveenraj Elancheliyan,[1,2] Klaudia K. Maruszczak,[1] Remigiusz Adam Serwa,[1] Till Stephan,[3,4,5] Ahmet Sadik Gulgec,[1] Mayra A. Borrero-Landazabal,[1] Sonia Ngati,[1] Aleksandra Gosk,[6] Stefan Jakobs,[3,4,7] Michal Wasilewski,[1,*] and Agnieszka Chacinska[1,2,8,*]
[1]IMol Polish Academy of Sciences, 02-247 Warsaw, Poland
[2]ReMedy International Research Agenda Unit, IMol Polish Academy of Sciences, 02-247 Warsaw, Poland
[3]Department of NanoBiophotonics, Max Planck Institute for Multidisciplinary Sciences, 37077 Göttingen, Germany
[4]Department of Neurology, University Medical Center Göttingen, 37075 Göttingen, Germany
[5]Buchmann Institute for Molecular Life Sciences, Goethe University Frankfurt am Main, Frankfurt am Main 60438, Germany
[6]Centre of New Technologies, University of Warsaw, 02-097 Warsaw, Poland
[7]Fraunhofer Institute for Translational Medicine and Pharmacology ITMP, Translational Neuroinflammation and Automated Microscopy TNM, 37075 Göttingen, Germany
[8]Lead contact
*Correspondence: m.wasilewski@imol.institute (M.W.), a.chacinska@imol.institute (A.C.)
https://doi.org/10.1016/j.celrep.2024.115038

## SUMMARY

Mitochondrial proteins are transported and sorted to the matrix or inner mitochondrial membrane by the presequence translocase TIM23. In yeast, this essential and highly conserved machinery is composed of the core subunits Tim23 and Tim17. The architecture, assembly, and regulation of the human TIM23 complex are poorly characterized. The human genome encodes two paralogs, TIMM17A and TIMM17B. Here, we describe an unexpected role of the ovarian cancer immunoreactive antigen domain-containing protein 1 (OCIAD1) and the prohibitin complex in the biogenesis of human TIM23. Prohibitins were required to stabilize both the TIMM17A- and TIMM17B-containing variants of the translocase. Interestingly, OCIAD1 assembled with the prohibitin complex to protect the TIMM17A variant from degradation by the YME1L protease. The expression of OCIAD1 was in turn regulated by the status of the TIM23 complex. We postulate that OCIAD1 together with prohibitins constitute a regulatory axis that differentially regulates variants of human TIM23.

## INTRODUCTION

Mitochondria are indispensable eukaryotic organelles that play a role in a plethora of cellular processes, such as oxidative phosphorylation, fatty acid β-oxidation, and cell-death regulation. The vast majority of mitochondrial proteins are encoded in nuclear DNA, translated on cytosolic ribosomes, and imported into mitochondria via protein complexes called translocases. Translocases comprise several molecular machineries that recognize various signals embedded in sequences of mitochondrial precursor proteins and manage their translocation into their target submitochondrial compartments.[1–3] The presequence translocase of the inner membrane (TIM23) is a protein complex that is localized in the inner mitochondrial membrane (IM) and recognizes an N-terminal signal composed of an amphipathic helix, called a presequence.[4,5] Proteins that are imported by TIM23 constitute the broadest class of mitochondrial proteins and localize to the mitochondrial matrix, IM, or the intermembrane space (IMS). The energy needed for TIM23-dependent protein import comes from the mitochondrial membrane electrochemical potential, which drives the electrophoretic transport of presequences across the IM, and ATP, which is required for chaperone-mediated translocation into the matrix. To interact with proteins of diverse target topologies, the TIM23 complex associates with a suite of accessory subunits that govern the full translocation of mitochondrial precursor proteins into the matrix or their lateral release into the IM.[6–8] In yeast, the core of the TIM23 complex consists of the essential subunits Tim17 and Tim23. Despite recent discoveries that revealed the structure of the TIM23 complex core,[9–11] the mechanisms that govern its assembly remain poorly understood. Indirect evidence points to an important role of the Tim17 disulfide bonds in this process.[12,13]

The human TIM23 complex adds another level of complexity due to the existence of two human orthologs of yeast Tim17, namely TIMM17A and TIMM17B. The significance of this duplication and its relation to the assembly of TIM23 is unclear. Both paralogs assemble with TIMM23 into the functional TIM23 complex and form separate pools of the translocase.[14] To date, however, data that support significant functional differences are lacking. Both paralogs may be differentially regulated, as TIMM17A is short-lived and is readily degraded in response to


This is an open access article under the CC BY license (http://creativecommons.org/licenses/by/4.0/).

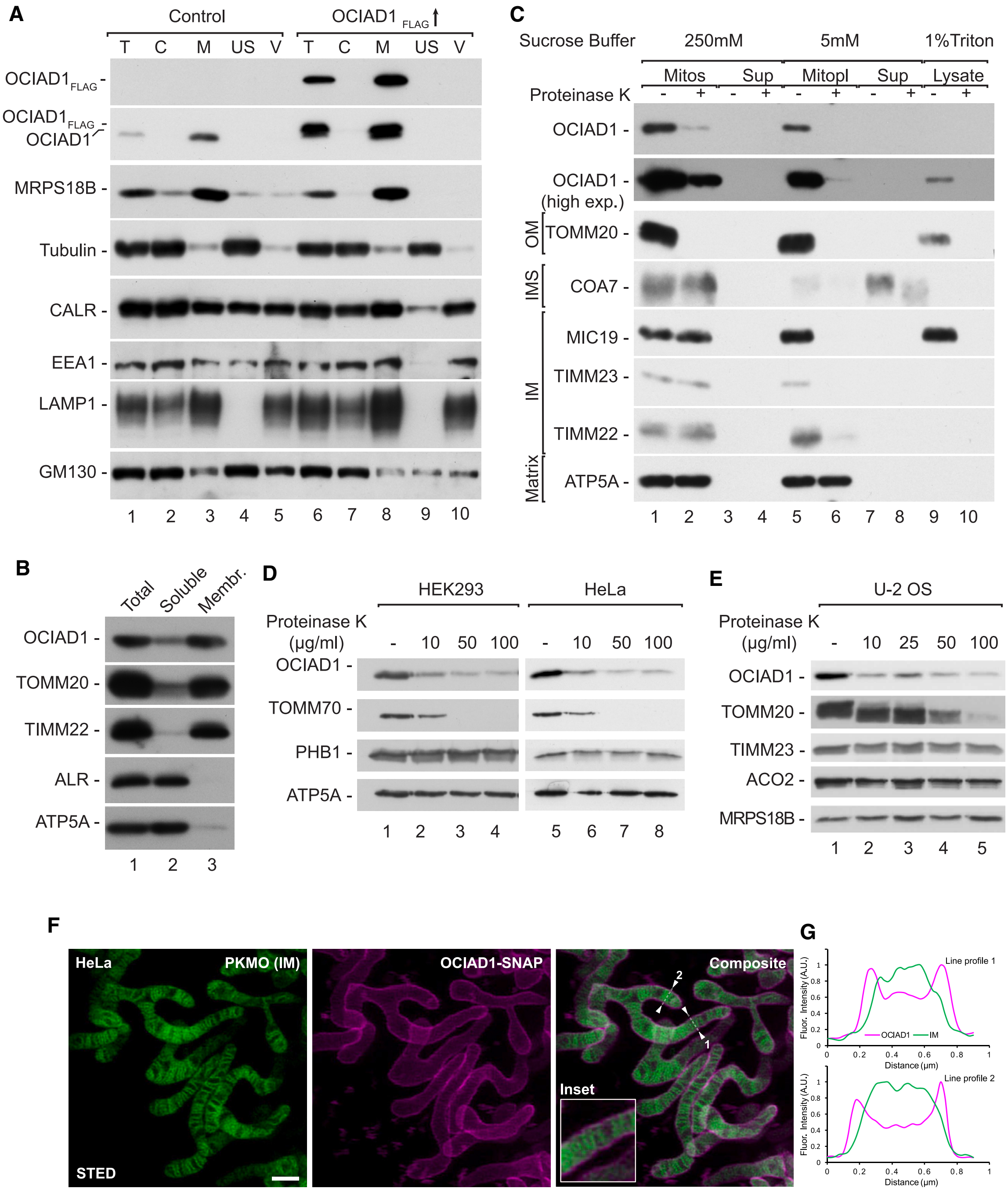


**Figure 1. OCIAD1 localizes predominantly in the outer membrane of mitochondria**

(A) Subcellular fractionation of HEK293 cells that expressed $OCIAD1_{FLAG}$ and an empty vector. T, total; C, cytosol; M, mitochondria; US, ultracentrifugation supernatant; V, light membranes.

(B) Extraction of proteins by sodium carbonate. Samples were analyzed by SDS-PAGE and western blot. Membr., membranes.

(C) Localization of mitochondrial proteins, analyzed by limited degradation with proteinase K in intact mitochondria (250 mM sucrose), mitoplasts (5 mM sucrose), and mitochondrial lysates (1% Triton X-100). The samples were analyzed by SDS-PAGE and western blot. Mitos, mitochondria; Mitopl, mitoplasts; Sup, supernatant; OM, outer membrane; IM, inner membrane; IMS, intermembrane space.

(D and E) Mitochondria isolated from HEK293 and HeLa (D) or U-2 OS (E) cells were treated with increasing concentrations of proteinase K. The samples were then analyzed with SDS-PAGE followed by western blotting.

*(legend continued on next page)*

unbalanced cellular homeostasis.[15–17] Although being of utmost importance for protein translocation, our current knowledge about the mechanism and the biogenesis of the human TIM23 complex and the functional differences between the two variants of TIMM17 is limited. For clarity, we refer to the subunits of the translocases present in human cells using protein names with double "M" (e.g., TIMM17A), while the names of translocases are abbreviated with a single "M" as adopted across different model organisms (e.g., TIM23, TIM22).[18]

In the present study, we identified the ovarian cancer immunoreactive antigen domain-containing protein 1 (OCIAD1) as a regulator of the TIM23 complex in human cells. We demonstrated that OCIAD1 interacts with TIM23 and prohibitins. We also found that the prohibitin complex is crucial for the biogenesis of the TIM23 complex and that OCIAD1 is required to differentiate between variants of the TIM23 translocase. In particular, we found that the deletion of OCIAD1 negatively affected the TIMM17A-containing and not the TIMM17B-containing TIM23, as in the absence of OCIAD1 the TIMM17A variant of the TIM23 complex was rapidly degraded by the YME1L1 protease. Lastly, we demonstrated that the defective TIM23 complex triggered the mitochondrial accumulation of OCIAD1, which potentially acts to rebuild the functional translocases. We propose that OCIAD1 and the prohibitin complex form a regulatory axis that senses the functional states of TIM23 machinery and governs the translocase fate.

## RESULTS

### OCIAD1 is localized in the outer mitochondrial membrane

OCIAD1 was shown to be present in endosomes and mitochondria.[19–21] To verify its intracellular localization, we fractionated HEK293 cells transiently expressing $OCIAD1_{FLAG}$ or the corresponding empty vector. We detected the endogenous OCIAD1 and $OCIAD1_{FLAG}$ in the mitochondrial fraction (Figure 1A, lanes 3 and 8). OCIAD1 was previously reported to be localized in either the outer mitochondrial membrane (OM) or the IM.[22,23] To test the submitochondrial localization of OCIAD1, we first performed a sodium carbonate extraction assay, employing the highly alkaline sodium carbonate solution (pH 10.8), which solubilizes and strips loosely associated proteins from cell membranes. OCIAD1 was resistant to extraction, indicating that it is very likely an integral membrane protein (Figure 1B, lane 3). Next, we used limited degradation by proteinase K in the isolated mitochondria from HEK293 cells to distinguish whether OCIAD1 is integrated into the OM or the IM. Proteinase K degraded OCIAD1 to a similar extent as the OM protein TOMM20 (Figure 1C, lanes 1 and 2). Upon rupturing the OM with hypo-osmotic swelling in 5 mM sucrose buffer, proteinase K degraded the remaining minor pool of OCIAD1, which was protected in intact mitochondria (Figure 1C, lanes 5 and 6). This suggests the OM localization of OCIAD1, with a small pool of OCIAD1 possibly also present in the IM. OCIAD1 was previously reported to localize exclusively to the IM of U-2 OS cells.[23] To verify whether the localization of OCIAD1 depends on cell type, we isolated mitochondria from HEK293, HeLa, and U-2 OS cells and titrated the amount of proteinase K in the assay (Figures 1D and 1E). OCIAD1 was largely degraded at the lowest proteinase K concentration (10 μg/mL), similar to the OM proteins TOMM70 and TOMM20. A minor resistant pool of OCIAD1 was present even at higher concentrations of proteinase K, while TOMM70 and TOMM20 were entirely degraded. These data further support our conclusion that OCIAD1 is mostly localized to the OM, similar to the localization reported by Antonicka et al.[22]

To further validate the submitochondrial localization of OCIAD1, we transiently overexpressed OCIAD1-SNAP in HeLa cells and investigated its localization by live-cell 2D stimulated emission depletion (STED) super-resolution microscopy. To this end, we labeled the cells with SNAP-cell 647-SiR[24] and the lipophilic IM marker PK Mito Orange (PKMO).[25] The live-cell recordings supported the notion that OCIAD1-SNAP was localized primarily in the OM, as the OCIAD1 signal was clearly shifted toward the periphery of the organelle when compared to the PKMO signal (Figures 1F and 1G). To rule out the possibility that the relatively large SNAP tag affects the submitochondrial localization of the protein, we next expressed OCIAD1-FLAG in HEK293 and U-2 OS cells and performed immunolabeling using antibodies against FLAG, TOMM20 (OM), MIC60 (inner boundary membrane), and ATPB (crista membrane) (Figures S1A and S1B). By measuring fluorescence intensity line profiles across the mitochondrial tubules, we investigated the submitochondrial distribution of the protein clusters revealed by STED microscopy. Corroborating our live-cell STED data, we found that OCIAD1, like TOMM20, was found on the periphery of the mitochondrial tubule, whereas the location of ATPB, marking the crista membrane, was generally shifted toward the center of the mitochondrial tubules (Figures S1A and S1B). These data further support the conclusions drawn from the biochemical analysis, indicating that OCIAD1 is primarily localized in the OM. However, we note that the optical resolution of the 2D STED microscope did not permit us to distinguish localization in the OM from one in the inner boundary membrane, the distance between these two membranes being smaller than the resolution limit of the microscope.

To decipher the topology of OCIAD1 in the OM, we analyzed its sequence with DeepTMHMM,[26] which predicted that OCIAD1 contains two transmembrane regions (Figure S2A). Thus, to examine which part of the protein is exposed to the cytosol, we treated intact and swelled mitochondria with proteinase K and used an antibody against the carboxy terminus of OCIAD1. We did not detect any proteolytic fragments of the protein, suggesting that both the N and C termini of OCIAD1 are exposed to the cytosol (Figure S2B, lanes 2 and 4). To further

(F and G) Submitochondrial localization of OCIAD1-SNAP in HeLa cells visualized by live-cell 2D STED microscopy. Cells were transfected with a plasmid encoding OCIAD-SNAP and labeled with SNAP-cell 647-SiR and the inner membrane (IM) marker PK Mito Orange (PKMO). The image shows a 2D projection of the mitochondrial tubules. (F) Representative dual-color STED recording. (G) Fluorescence intensity line profiles were measured at the sites indicated by arrowheads in the composite view. The fluorescence intensity was estimated along the transparent dashed lines, normalized, and plotted. Scale bar: 1 μm.
See also Figures S1 and S2.

substantiate this finding, we expressed OCIAD1 tagged on either the N or C terminus with hemagglutinin (HA) and tested its degradation in mitochondria (Figures S2C and S2D). Similarly to the native OCIAD1, both N- and C-terminal HA tags were sensitive to the exogenously added protease. Therefore, we propose that both the N and C termini of OCIAD1 face the cytosol and that the loop between both transmembrane domains is positioned in the IMS. Overall, our results show the mitochondrial localization of OCIAD1 with the main pool of the protein in the OM, but we cannot exclude the presence of a minor pool of the protein in the inner boundary membrane.

It was previously demonstrated that OCIAD1 forms high-molecular-mass complexes.[23] We performed blue native electrophoresis in samples with small interfering RNA (siRNA)-depleted OCIAD1 (Figure S2E). Most of the signal was sensitive to OCIAD1 depletion, demonstrating its specificity except for one unspecific band. In line with previous reports, we observed OCIAD1-specific signals mostly in the high-molecular-weight part of the gel corresponding to protein complexes larger than 669 kDa.

## OCIAD1 interacts with the prohibitin complex and protein translocases localized in the mitochondrial IM

To discover interaction partners of OCIAD1, we performed mass spectrometry analysis of proteins that co-purified with OCIAD1$_{FLAG}$ expressed in HEK293 cells. Among 197 mitochondrial proteins that were identified in our analysis, 28 were significantly enriched in the OCIAD1$_{FLAG}$ sample (Figure 2A and Table S1). Among the most enriched proteins, we found components of the prohibitin complex (PHB2, PHB, and AFG3L2) and the MICOS complex (MIC19). To validate our findings, the affinity-purified interactors of OCIAD1$_{FLAG}$ were analyzed by western blotting (Figures 2B, S3A, and S3B). We confirmed the interaction with all three components of the prohibitin complex and MIC19. In addition, we detected subunits of mitochondrial translocases (TIMM23, TIMM22, and TOMM20). Although peptides corresponding to these proteins were not detected in the mass spectrometry analysis, their enrichment in the western blot was comparable to that in PHB2 and MIC19 (Figure S3B). Interaction of OCIAD1 with the prohibitin complex has been previously reported.[23,27–30] To validate those observations, we expressed HA-tagged PHB2 in HEK293T cells, isolated mitochondria, and performed western blotting of affinity-purified PHB2 interactors (Figure 2C). The PHB protein, which is a known partner of PHB2 in the prohibitin complex, was the most efficiently co-purified protein.[31] We also co-purified OCIAD1, confirming its interaction with PHB2. The efficiency of affinity purification of OCIAD1 and PHB suggested that OCIAD1 was a substoichiometric partner of PHB2. In addition, we identified subunits of the TIM23 translocase, TIMM17A, TIMM17B, and TIMM23, among the interactors of PHB2, suggesting that the prohibitin complex interacted with the core of TIM23 translocase.

To validate interactions of OCIAD1 with subunits of TIM23 translocase, we performed reversed affinity purifications using components of the translocase as bait. We isolated mitochondria from HEK293T cells that expressed C-terminally tagged TIMM23$_{FLAG}$ and TIMM21$_{FLAG}$ and detected the interaction partners with western blotting. Both TIMM23$_{FLAG}$ and TIMM21$_{FLAG}$ interacted with OCIAD1, in line with the previously published mass spectrometry results (Figure 2D, lanes 5 and 6).[32] The OCIAD1 interaction with TIMM23$_{FLAG}$ and TIMM21$_{FLAG}$ was weaker than the interaction with their stoichiometric partner, TIMM17A. To further confirm the interactions of TIM23 subunits with OCIAD1, we isolated mitochondria and affinity purified the interactors of heterologously expressed HA-tagged TIMM17A and TIMM17B (Figures 2E and S3C). In both cases, OCIAD1 was reproducibly co-purified with efficiency lower than that of TIMM23, a known stoichiometric partner of both TIMM17A and TIMM17B. Notably, we observed co-purification of PHB2 with both versions of TIMM17, recapitulating our results of affinity purification with HA-PHB2 as the bait (Figures 2E and S3C). Altogether, our data demonstrate interactions between the prohibitin complex, the TIM23 translocase, and OCIAD1. The exact mechanism of these interactions as well as their sequence remains to be established.

The fact that OCIAD1 interacts with TIMM23 prompted us to explore whether TIM23 translocase affects OCIAD1 biogenesis. In yeast, several OM proteins were reported to use the TIM23 complex for their import and assembly in the OM.[33,34] Therefore, we performed the *in organello* import of OCIAD1 into mitochondria isolated from cells depleted of TIMM23 by RNA interference and analyzed it by blue native electrophoresis. The radiolabeled OCIAD1 formed a high-molecular-mass complex similar to the OCIAD1-specific signal obtained by western blot (Figure S4A). We found that the import of OCIAD1 was resistant to the depletion of TIMM23 (Figures S4A and S4B). Interestingly, the assembly of the OCIAD1 complex was compromised by the depletion of mitochondrial inner membrane potential with a mixture of valinomycin, oligomycin, and antimycin A. In agreement with TIMM23 depletion, the activity of the TIM23 pathway was efficiently inhibited, as documented by *in organello* import of classic presequence-containing TIM23 substrates SCO2 and OTC (Figures S4C and S4D). Our data show that the TIM23 translocase is dispensable for the import and assembly of OCIAD1, although the biogenesis of OCIAD1 requires mitochondrial inner membrane electrochemical potential.

## OCIAD1 assembles with the prohibitin complex to regulate TIM23 translocase

To understand the relationship between OCIAD1 and the prohibitin complex, we verified the biogenesis of PHB2 in OCIAD1-knockout (-KO) cells (Figures 3A and S5A). We found that steady-state levels of PHB2 were unaffected, similar to another IM protein, TIMM22. It was recently reported that OCIAD1 assembles with the prohibitin complex, but the depletion of OCIAD1 does not affect the complex presence.[23] To verify whether in our model OCIAD1 influenced the prohibitin complex, we compared protein complexes using blue native electrophoresis in wild-type and OCIAD1-KO cells and in cells in which OCIAD1 expression was reintroduced by the transient transfection (Figure 3B). The knockout of OCIAD1 induced a decrease in prohibitin complex levels, and this phenotype was partially rescued by exogenous expression of OCIAD1. To further confirm that OCIAD1 affects the prohibitin complex, we performed additional experiments with siRNA-depleted OCIAD1 and analyzed mitochondrial protein complexes with blue native electrophoresis. As expected, the prohibitin complex was decreased

CellPress
OPEN ACCESS

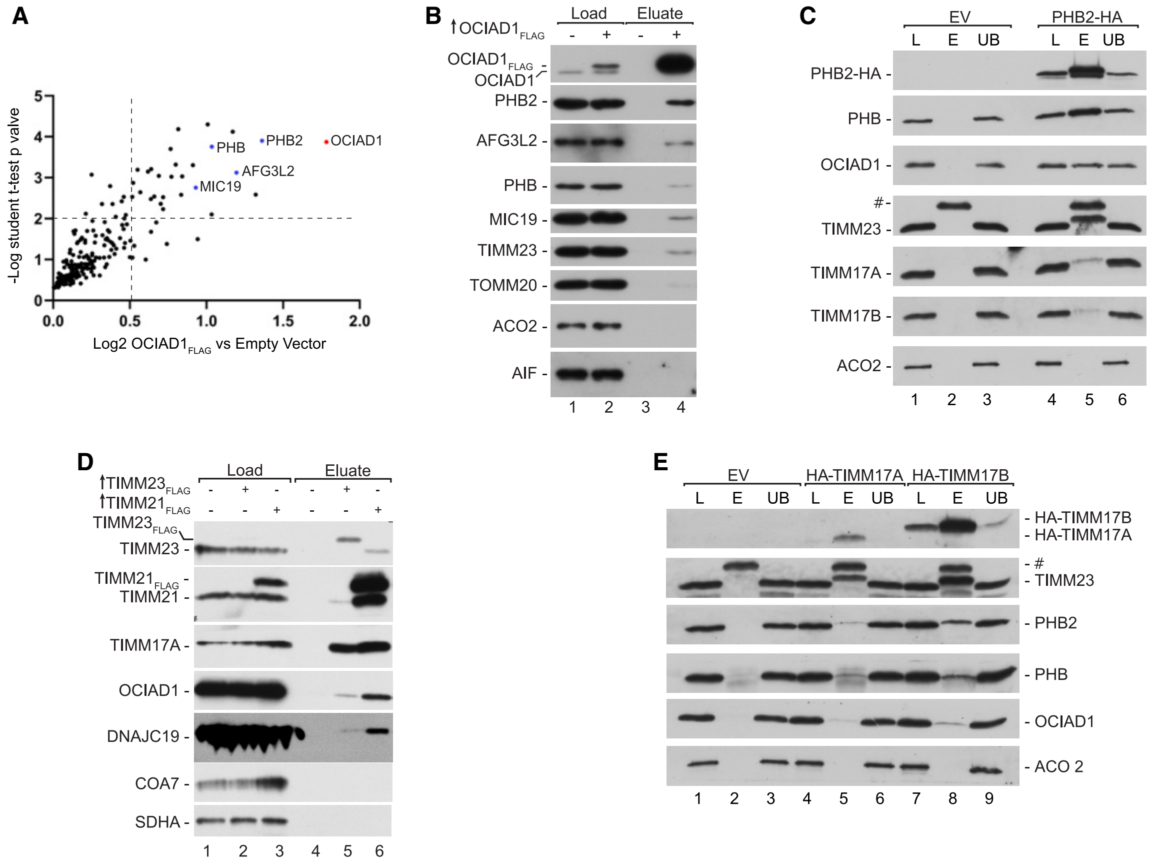


**Figure 2. OCIAD1 interacts with the prohibitin complex and the TIM23 complex**

(A) Enrichment of proteins co-purified with OCIAD1$_{FLAG}$. Mitochondria were isolated from HEK293 cells that expressed OCIAD1$_{FLAG}$ or an empty vector, solubilized in digitonin-containing buffer, and subjected to immunoprecipitation with FLAG affinity resin. The FLAG-peptide eluted fraction was trypsinized, desalted, and labeled with TMT. The samples were analyzed by LC-MS/MS ($n$ = 3).

(B) Affinity purification of OCIAD1$_{FLAG}$ as in (A). Affinity-purified proteins were eluted with Laemmli buffer. Samples were analyzed by SDS-PAGE and western blot. Load: 2%; eluate: 100%.

(C) Affinity purification of PHB2-HA. Mitochondria were isolated from HEK293T cells that expressed PHB2-HA or an empty vector, solubilized in digitonin-containing buffer, and subjected to immunoprecipitation with HA affinity resin. Samples were analyzed by SDS-PAGE and western blot. L, load (3%); E, elution (100%); UB, unbound (3%); #, antibody light chain.

(D) Affinity purification of TIMM21$_{FLAG}$ and TIMM23$_{FLAG.}$ Mitochondria from TIMM21$_{FLAG}$, TIMM23$_{FLAG}$, or empty-vector-transfected HEK293T cells were solubilized in a digitonin-containing buffer and subjected to immunoprecipitation with FLAG affinity resin. Samples were analyzed by SDS-PAGE and western blot. Load: 2%; eluate: 100%.

(E) Affinity purification of HA-TIMM17A and HA-TIMM17B. Mitochondria were isolated from HEK293T cells that expressed HA-TIMM17A, HA-TIMM17B, or an empty vector, solubilized in digitonin-containing buffer, and subjected to immunoprecipitation with HA affinity resin. Samples were analyzed by SDS-PAGE and western blot. L, load (3%); E, elution (100%); UB, unbound (3%); #, antibody light chain.

See also Figures S3 and S4.

in response to siRNA-mediated depletion of OCIAD1 (Figure 3C). In line and in agreement with previously published data,[23] the prohibitin complex and OCIAD1 migration partially overlapped in the native gel (Figures 3B and 3C). To directly test whether OCIAD1 assembles with the prohibitin complex, we imported radiolabeled OCIAD1 into the mitochondria of PHB2-silenced cells. The assembly of OCIAD1 into the high-molecular-weight complex was compromised in PHB2-depleted mitochondria (Figure 3D). Thus, we established that OCIAD1 participates in the prohibitin complex and is important for its stability.

To further analyze the relationship of the prohibitin complex with OCIAD1, we analyzed OCIAD1 abundance in PHB2-depleted cells. We found that while OCIAD1 was unaffected, unexpectedly the depletion of PHB2 decreased the levels of core subunits of TIM23, including TIMM23, TIMM17A, and TIMM17B (Figures 4A and 4B). Several other IM proteins (TIMM22, ANT2, and DNAJC19) as well as a matrix protein (SDHA) remained unchanged. The decrease in the abundance of TIMM17A and TIMM17B in response to siRNA-mediated silencing of PHB2 suggests that both versions of the TIM23 translocase are affected in PHB2-depleted cells. Accordingly, blue native electrophoresis revealed significantly lower levels of the TIM23 translocase in the PHB2-depleted cells (Figure 4C). Altogether, these data indicate that the prohibitin complex controls the biogenesis of TIMM17A- and

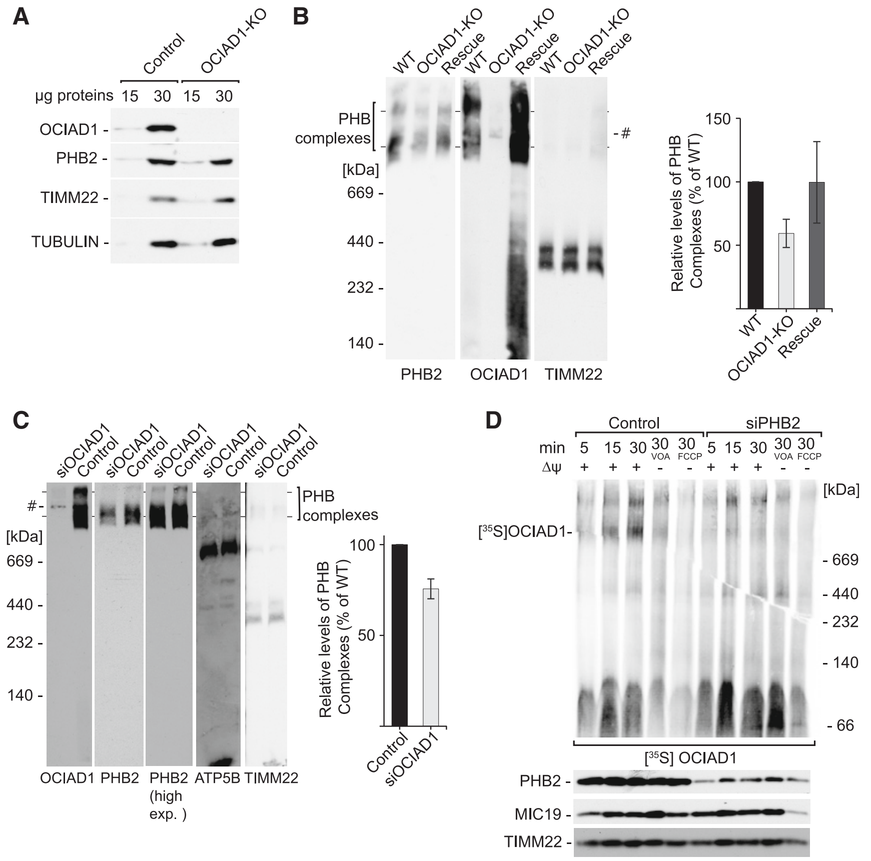


**Figure 3. OCIAD1 assembles with the prohibitin complex**

(A) Western blot analysis of whole-cell extracts of HEK293 wild-type and OCIAD1-KO cells. The samples were analyzed by SDS-PAGE and western blot.

(B) Protein complexes in mitochondria that were isolated from HEK293 wild-type (WT), OCIAD1-KO, and exogenous OCIAD1-expressing OCIAD1-KO cells (Rescue), solubilized with 1% digitonin-containing buffer, and resolved in 3%–13% blue native (BN)-PAGE gels, followed by western blot. The quantification of PHB complexes is presented as mean ± SEM ($n$ = 3). #, unspecific band.

(C) Protein complexes in mitochondria that were isolated from HEK293 with native or siRNA-depleted levels of OCIAD1, solubilized with 1% digitonin-containing buffer, and resolved in 3%–13% BN-PAGE gels, followed by western blot. The quantification of PHB complexes is presented as mean ± SEM ($n$ = 3). Control, cells transfected with Mission siRNA universal negative control; #, unspecific band.

(D) *In organello* import of OCIAD1. Radiolabeled [$^{35}$S] OCIAD1 precursors that were imported into mitochondria isolated from HEK293 control and PHB2 siRNA-depleted cells were resolved by 3%–13% BN-PAGE gel and analyzed by autoradiography or were resolved by SDS-PAGE and analyzed by western blot Control, cells transfected with Mission siRNA universal negative control; VOA, valinomycin, antimycin A, and oligomycin; FCCP, carbonyl cyanide-*p*-trifluoromethoxyphenylhydrazone.

See also Figure S5.

TIMM17B-containing versions of the TIM23 translocase and that the prohibitin complex is itself controlled by OCIAD1.

## OCIAD1 promotes the TIMM17A variant of the TIM23 complex

Considering the presented evidence for reciprocal interactions between OCIAD1, the prohibitin complex, and the TIM23 translocase, we asked whether OCIAD1 is involved in the control of the TIM23 translocase. To this end, we analyzed protein complexes in the mitochondria of OCIAD1-KO cells by blue native electrophoresis (Figures 5A and 5B). We found that the TIM23 complex was decreased in OCIAD1-KO cells and that the restoration of OCIAD1 levels by the transient transfection rescued TIM23 complex formation. To describe the depletion of TIM23 translocase more precisely, we analyzed the TIM23 complexes with antibodies against TIMM17A and TIMM17B. Surprisingly we discovered that while the TIMM17A-containing complex was severely decreased in OCIAD1-KO cells, the TIMM17B-containing complex was not affected. Further, we corroborated the discriminative influence on both versions of the TIM23 translocase by comparing the abundance of the TIM23 complex subunits. Western blot analysis of mitochondria from OCIAD1-KO cells revealed the downregulation of TIMM17A, TIMM21, and TIMM23 but not TIMM17B (Figure 5C). TIMM17A, TIMM21, and TIMM23 displayed a tendency to increase after the restoration of OCIAD1 levels. To further explore the discriminative control of OCIAD1 over TIM23 translocases, we used an alternative strategy to deplete OCIAD1 by transfecting cells with an siRNA against OCIAD1. We analyzed mitochondrial protein complexes in OCIAD1-depleted cells. As expected, levels of the TIM23 complex were decreased, whereas unrelated protein complexes, such as the TOM complex and OXPHOS complex II, remained unchanged (Figure S6A). Analysis of steady-state levels of the TIM23 complex revealed that similar to genetic ablation, the silencing of OCIAD1 expression resulted in the significant downregulation of TIMM23 and TIMM17A but not TIMM17B (Figure S6B). Therefore, our results indicate that the two variants TIMM17A and TIMM17B that form separate pools of the TIM23 translocase are differentially regulated in the absence of OCIAD1.

As mentioned before, the TIM23 complex is known to exist in two functional modules that are responsible for protein localization in the matrix or the IM.[14] Therefore, we extended the analysis to discern which of the two modules of the TIM23 complex is depleted in the absence of OCIAD1. We imported two substrates of TIM23 into the mitochondria of OCIAD1-KO and OCIAD1-silenced cells: COX4I1 (which is sorted into the IM) and ornithine transcarbamylase (OTC) (which is fully translocated into the matrix). As expected, the depletion of OCIAD1 resulted in the significantly lower efficiency of import in both protein substrates, suggesting that TIMM17A participates in both activities of the TIM23 complex (Figures 5E, 5F, S7A, and S7B). We also tested levels of mitochondrial inner membrane potential in OCIAD1-KO cells to

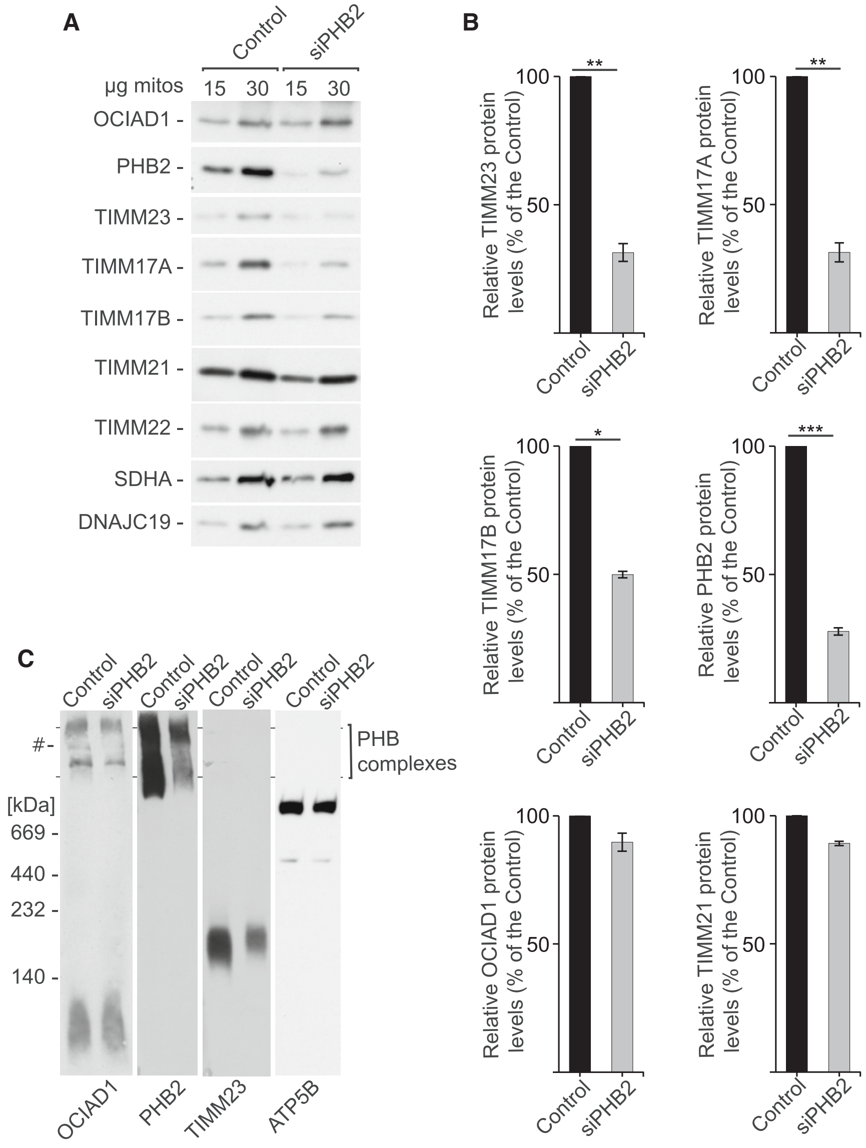


**Figure 4. Prohibitins are essential for the biogenesis of the TIM23 translocase**

(A) Western blot analysis of mitochondria that were isolated from control and PHB2 siRNA-depleted cells.

(B) Quantification of protein signals in (A) from lanes that were loaded with 30 μg mitochondria is presented as mean ± SEM ($n = 3$). $^{*}p < 0.05$, $^{**}p < 0.01$, $^{***}p < 0.001$ (two-tailed Student's t test).

(C) Protein complexes in mitochondria that were isolated from HEK293 cells with native or siRNA-depleted levels of PHB2, solubilized with 1% digitonin-containing buffer, and resolved in 3%–13% BN-PAGE gels, followed by western blot. Control, cells transfected with Mission siRNA universal negative control; #, unspecific band.

understand whether it is affected by the absence of OCIAD1, thus leading to the decreased import of IM proteins. On the contrary, we observed a significant increase of the mitochondrial inner membrane electrochemical potential in OCIAD1-KO cells, further indicating that compromised protein import was a consequence of lower levels of TIM23 (Figure S8A). To further investigate the relationship between OCIAD1 and the TIM23 complex, we performed qPCR for OCIAD1-KO cells. We did not observe a significant difference in mRNA levels of TIM23 subunits in OCIAD1-deficient cells compared with the wild-type cells (Figure S8B). Therefore, we concluded that OCIAD1 controls the formation or stability of the TIM23 complex through its TIMM17A variant.

## YME1L1 is responsible for the degradation of the TIMM17A variant of the TIM23 complex

The decrease in the TIM23 complex in OCIAD1-KO cells prompted us to investigate the activity of the TIM22 pathway, which is responsible for the import and membrane insertion of core TIM23 subunits such as TIMM23, TIMM17A, and TIMM17B. We performed the *in organello* import of four substrates of the TIM22 complex, TIMM23, TIMM17A, TIMM17B, and ANT3, and analyzed their complex assembly using blue native electrophoresis. We observed a significant decrease in the assembly of radiolabeled subunits of TIM23 translocase TIMM23, TIMM17A, and TIMM17B in OCIAD1-KO cells, whereas the assembly of ANT3 was unaffected (Figures 6A, 6B, S9A, and S9B). We corroborated this finding in cells with siRNA-mediated depletion of OCIAD1 (Figures S9C and S9D). Furthermore, the levels of the TIMM22 protein and the TIM22 complex observed in OCIAD1-KO cells were the same as in the control samples (Figures 5A and 5C). Altogether, these data indicate that the TIM22 pathway remains functional in OCIAD1-depleted cells and that the apparent decrease of the TIM23 complex assembly results from other factors such as the insufficient levels of other subunits of the complex.

Interestingly, YME1L protease was shown previously to degrade TIMM23 and TIMM17A under stress conditions.[16,32] To verify whether YME1L1 is involved in the degradation of the TIMM17A variant of the TIM23 complex in OCIAD1-KO cells, we treated cells with siRNA that targeted YME1L mRNA. Indeed, the depletion of YME1L protease rescued TIMM17A and TIMM23 levels in OCIAD1-KO cells, whereas it had a minimal effect on the presence of TIMM17B (Figures 6C and 6D). Thus, we established that OCIAD1 protects the TIMM17A variant of the TIM23 complex from degradation by YME1L.

## Depletion of the TIMM17B-containing TIM23 complex triggers OCIAD1 accumulation in mitochondria

We compared the effects of TIM23 core subunit depletion on OCIAD1. The ablation of TIMM23 by siRNA led to a marked

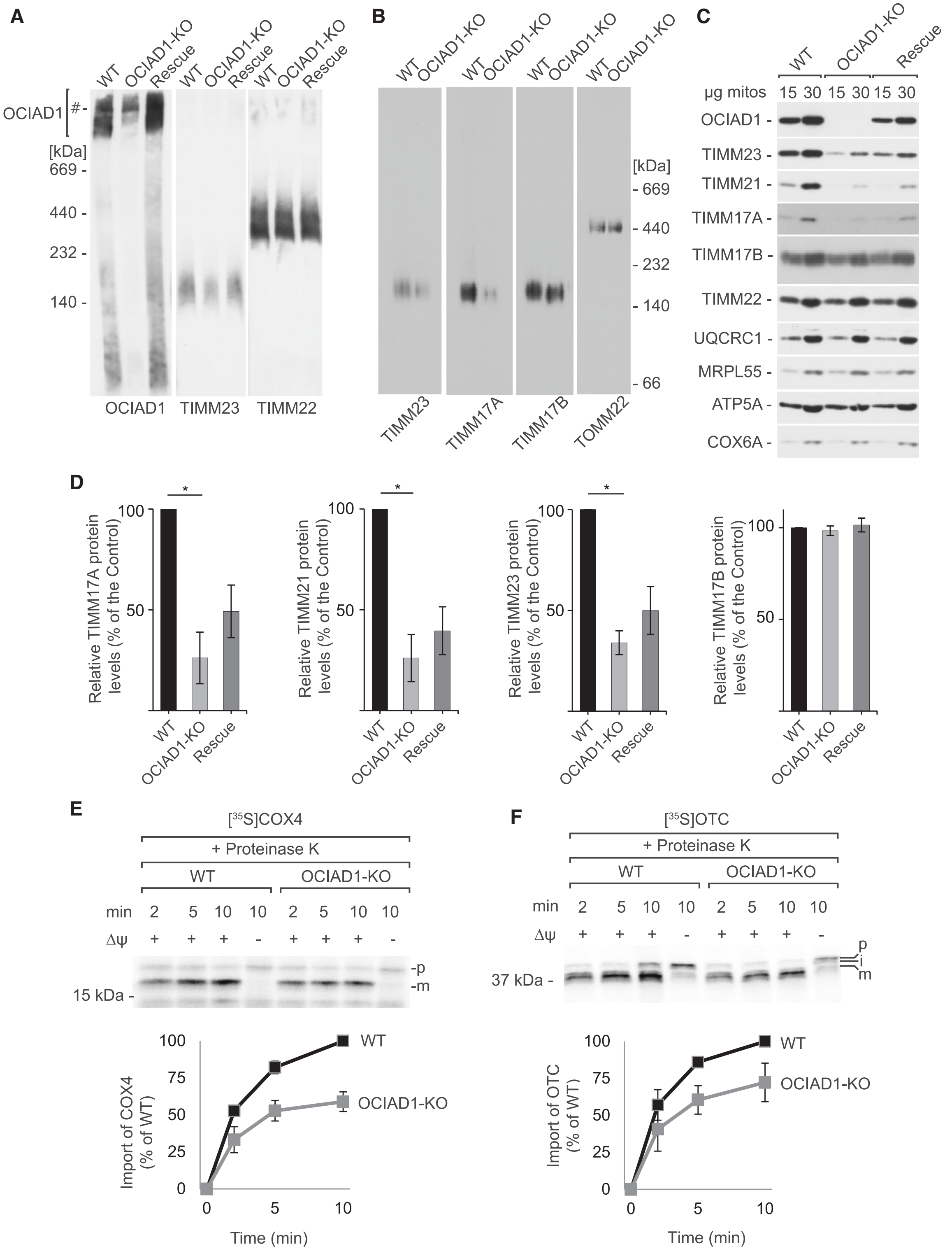


**Figure 5. OCIAD1 controls TIMM17A-containing variant of the TIM23 translocase**

(A) Protein complexes in mitochondria that were isolated from HEK293 wild-type (WT) cells, OCIAD1-KO cells, and exogenous OCIAD1-expressing OCIAD1-KO cells (Rescue) solubilized with 1% digitonin-containing buffer, and resolved in 4%–13% BN-PAGE gels, followed by western blot. #, unspecific band.

(B) Protein complexes in mitochondria that were isolated from HEK293 wild-type and OCIAD1-KO cells, solubilized with 1% digitonin-containing buffer, and resolved in 3%–13% BN-PAGE gels, followed by western blot.

*(legend continued on next page)*

increase in OCIAD1 protein expression, although OCIAD1 mRNA levels remained unchanged, suggesting that the regulation of OCIAD1 levels is post-transcriptional (Figures 7A and 7B). We then asked whether the effect of TIMM23 depletion can be replicated by the depletion of its core partners, TIMM17A and TIMM17B. To this end, we compared OCIAD1 levels in the mitochondria isolated from cells transfected with siRNA against TIMM23, TIMM17A, or TIMM17B (Figures 7C, S10A, and S10B). Similarly to TIMM23 depletion, the knockdown of TIMM17B induced levels of OCIAD1 while depletion of TIMM17A had no effect, demonstrating a specific role of TIMM17B in this phenomenon. Depletion of TIMM17A and TIMM17B did not affect TIMM23 levels, suggesting that it is the abundance of TIM23 translocase variants and not the TIMM23 protein per se that controls OCIAD1 levels. Interestingly, we also observed a compensatory increase of TIMM17A and TIMM17B induced by the depletion of the corresponding paralog. This suggested the presence of a compensatory mechanism that coordinates the abundance of both versions of the TIM23 translocase (Figures S10A and S10B). To further explore the role of TIMM17B in the regulation of OCIAD1 levels, we used the TIMM17B WT/Del cell line with a partial depletion of the *TIMM17B* gene (Figure S10C). Both TIMM17B and TIMM23 levels were significantly decreased in TIMM17B WT/Del cells, while TIMM17A was more abundant than in the wild-type cells (Figure 7D). Accordingly, we observed the increase in OCIAD1 levels, corroborating the involvement of the TIMM17B-containing TIM23 translocase in the regulation of OCIAD1. To further explore the regulation of OCIAD1 abundance by TIM23 variants, we analyzed levels of protein complexes in TIMM23-, TIMM17A-, and TIMM17B-depleted cells (Figures 7E and 7F). As expected, the depletion of TIMM23 and TIMM17B increased complexes of OCIAD1, while depletion of TIMM17A had no such effect.

In summary, we found that the prohibitin complex in concert with OCIAD1 controls the biogenesis of the TIM23 translocase (Figure 7G). This regulatory axis senses the status of TIM23 machinery to regulate variants of the TIM23 translocase. OCIAD1 is specifically required to promote the stability of the TIMM17A-containing variant of the TIM23 translocase, which in the absence of OCIAD1 undergoes proteolytic turnover.

## DISCUSSION

Vertebrate genomes encode two paralogs of the yeast essential gene *Tim17*. Thus, human TIM23 can be composed of either TIMM17A or TIMM17B.[14] Our understanding of mechanisms that control both variants of the TIM23 translocase remains rudimentary. In the present study, we found that OCIAD1 is an important factor in a regulatory mechanism that controls the biogenesis of TIM23 translocase in humans. Our data demonstrate that prohibitins are vital for the biogenesis of the TIM23 complex that contains both paralogs, whereas OCIAD1 influences only the assembly of the TIMM17A form of the translocase. Vice versa, the TIM23 translocase influences the abundance of OCIAD1, suggesting that OCIAD1 and the prohibitin complex form a regulatory axis that fine-tunes both variants of the TIM23 translocase.

Prohibitins, together with the matrix AAA protease, form a large complex in the IM, which is structurally conserved and can be traced back to the prokaryotic origins of mitochondria.[35–38] The prohibitin complex is involved in maintaining mtDNA, membrane scaffolding, control of the lipid composition of the IM, and the proteolytic activity of *m*-AAA protease. The present study demonstrates the dependence of the TIM23 complex on the presence of the prohibitin complex. The depletion of PHB2 led to a substantial decrease in levels of both core subunits of the TIM23 translocase and the assembled complex. Both TIMM17A and TIMM17B proteins were affected, suggesting that the prohibitin complex controls all forms of the TIM23 complex. This dependence is further strengthened by the physical interaction between PHB2 and TIMM23 protein, which was demonstrated by us and others,[27] as well as with other core components of the translocase, TIMM17A, and TIMM17B. The role of the prohibitin complex in TIM23 biogenesis was not recognized previously, but a few clues pointed in this direction. Prohibitins interact with stomatin-like protein 2 (SLP2), a scaffold that controls the activity of YME1L, which in turn degrades subunits of the TIM23 complex.[15,16,39–41] Additionally, yeast prohibitins interact with nascent, unassembled proteins that are translated in the matrix by mitoribosomes, and this interaction controls the degradation of unassembled subunits of the respiratory chain by *m*-AAA protease.[42,43] We propose that the prohibitin complex acts as a platform to control the biogenesis of TIM23 translocases.

We demonstrated that the absence of OCIAD1 specifically affected the TIMM17A-containing variant of the translocase. This suggests that OCIAD1 plays a role as a sensor that, in concert with prohibitins, specifically controls the TIMM17A form of the translocase. Examples of the discriminative regulation of both translocases were previously limited to the stress-induced degradation of the short-lived TIMM17A by YME1L, the activity of which is controlled by SLP2.[15,16,40] Our results corroborate these findings. Interestingly, we demonstrate that OCIAD1 protects TIMM17A against YME1L-mediated degradation. Only a small pool of the translocase interacts with the prohibitin complex at a given moment, suggesting that protection that is conferred by OCIAD1 and prohibitins could be limited to a transient state of the translocase, such as its assembly. In this model, nascent subunits building the TIM23 complex could interact with OCIAD1 and the prohibitin complex for proper assembly, and supernumerary

(C) Western blot analysis of mitochondria that were isolated from wild-type HEK293, OCIAD1-KO, and exogenous OCIAD1-expressing OCIAD1-KO cells (Rescue). mitos, mitochondria.

(D) Quantification of protein signals in (C) from lanes that were loaded with 30 μg mitochondria is presented as mean ± SEM ($n = 5$). *$p < 0.05$ (two-tailed Student's t test).

(E and F) *In organello* import of [$^{35}$S]COX4 (E) and [$^{35}$S]OTC (F) precursors into mitochondria that were isolated from wild-type and OCIAD1-KO HEK293 cells. Samples were analyzed by SDS-PAGE and autoradiography. The quantification of autoradiography signals is presented as mean ± SEM ($n = 3$). The amount of imported protein into wild-type mitochondria after 10 min was set to 100%. VOA, valinomycin, antimycin A, and oligomycin; p, presequence form; i, intermediate form; m, mature form.

See also Figures S6–S8.

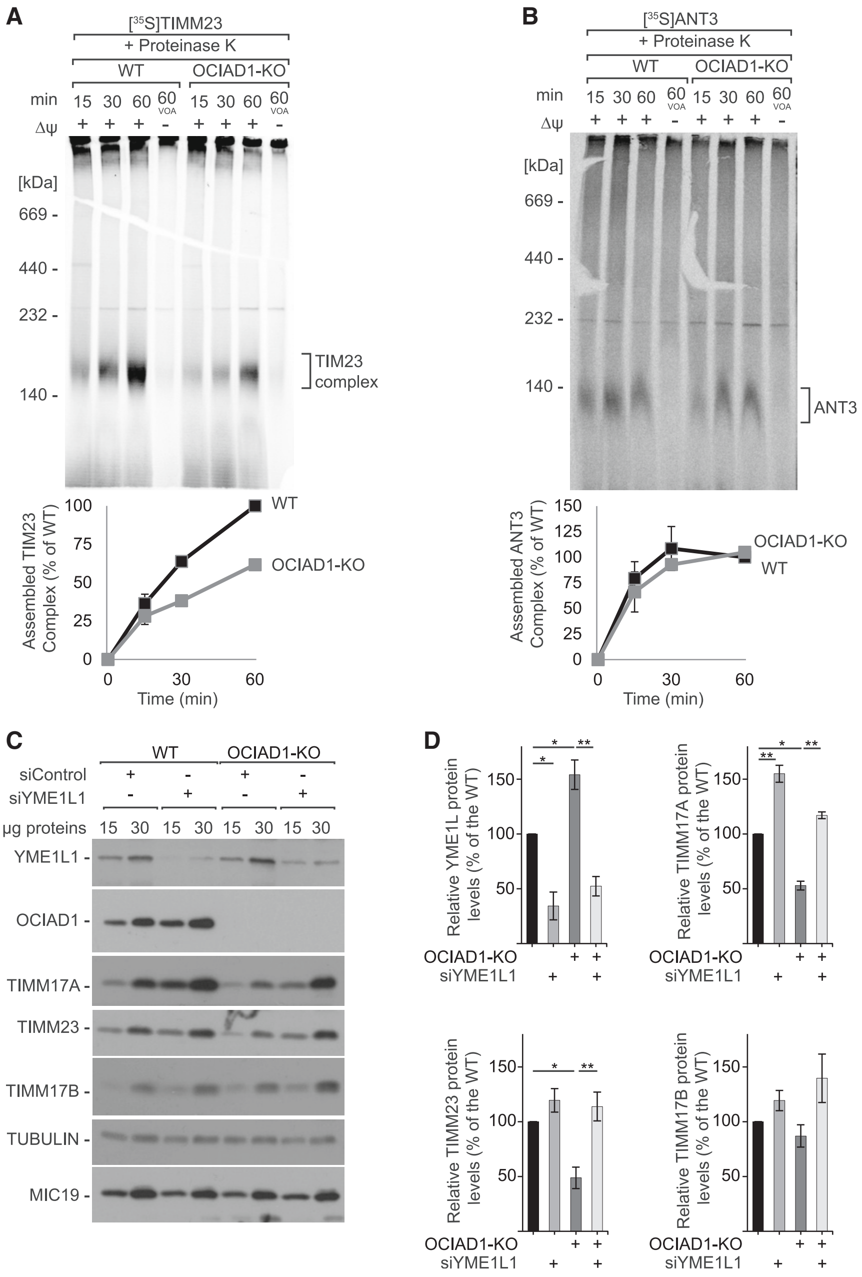


**Figure 6. OCIAD1 regulates the turnover of TIM23 complex subunits**

(A and B) *In organello* import of [$^{35}$S]TIMM23 (A) and [$^{35}$S]ANT3 (B) precursors into mitochondria that were isolated from wild-type and OCIAD1-KO HEK293 cells. Samples were analyzed by resolving in 4%–13% BN-PAGE gels and autoradiography. The quantification of autoradiography signals is presented as mean ± SEM ($n$ = 3). The amount of imported protein into wild-type mitochondria after 60 min was set to 100%. WT, wild type; VOA, valinomycin, antimycin A, and oligomycin.

(C) Western blot analysis of whole-cell extracts that were isolated from wild-type (WT) and OCIAD1-KO HEK293 cells with native or siRNA-depleted YME1L1.

(D) Quantification of protein signals from lanes in (C) that were loaded with 30 μg mitochondria is presented as mean ± SEM ($n$ = 3). *$p$ < 0.05, **$p$ < 0.01 (two-tailed Student's t test).

See also Figure S9.

subunits would undergo degradation by YME1L (Figure 7G). In line with the above hypothesis, we showed that the absence of OCIAD1 interferes with the assembly of imported TIMM23. Notably, the proper biogenesis of the yeast TIM23 complex depends on the formation of an intramolecular disulfide bond in Tim17.[12,13] Future studies should address whether OCIAD1 and the prohibitin complex could influence the formation of disulfide bonds in human orthologs of Tim17.

Intriguingly, OCIAD1 levels are controlled by the TIM23 complex, and this effect differently depends on TIMM17B and TIMM17A variants of the translocase. The depletion of TIMM17B and TIMM23 led to the significant accumulation of OCIAD1, whereas TIMM17A depletion did not have such an effect. Additionally, the depletion of either TIMM17A or TIMM17B led to an increase of the other paralog, suggesting a compensatory mechanism that synchronizes levels of both versions of the TIM23 translocase. The accumulation of OCIAD1 could contribute to this mechanism by protecting TIMM17A-containing translocase from YME1L-mediated degradation when levels of TIMM17B are insufficient (Figure 7G). Thus, OCIAD1 could act as a sensor that interprets cues about the state of the TIM23 machinery.

In conclusion, we identified a regulatory mechanism that controls TIM23 biogenesis through interaction with a membrane scaffolding platform organized by the prohibitin complex and OCIAD1. The mechanism controls TIMM17A and TIMM17B variants of the protein translocase and provides a means for their fine-tuning. Our findings contribute to a better understanding of the biogenesis of mitochondrial protein translocases and may reveal how selective degradation contributes to the biogenesis of mitochondrial complexes.

## Limitations of the study

In this study, we show that OCIAD1 controls the levels of the TIMM17A-containing TIM23 translocase. Our experiments localize OCIAD1 in the OM with its N and C termini facing the cellular cytosol; therefore, it remains to be elucidated whether OCIAD1 directly impacts the TIMM17A-containing TIM23 translocase or whether other, yet unidentified, proteins are involved in this regulatory axis. Additionally, our research does not explain

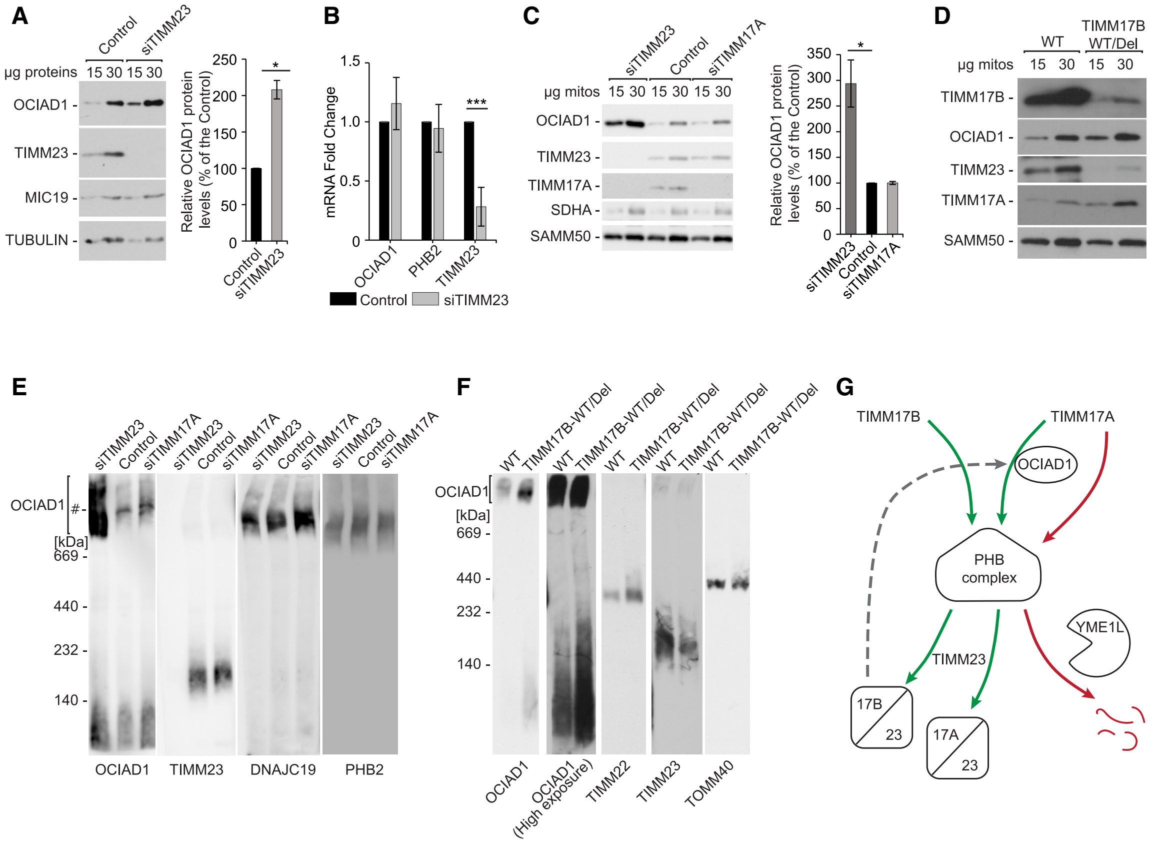


**Figure 7. OCIAD1 accumulates in cells with depletion of the TIMM17B variant of the TIM23 translocase**

(A) Western blot analysis of whole-cell extracts from HEK293 cells with native and siRNA-depleted TIMM23. The quantification of autoradiography signals from lanes that were loaded with 30 µg is presented as mean ± SEM ($n$ = 5). *$p$ < 0.05 (two-tailed Student's t test). Control, cells transfected with Mission siRNA universal negative control.

(B) mRNA expression patterns of selected transcripts in wild-type and OCIAD1-KO cells analyzed by RT-qPCR. mRNA levels are presented as fold changes relative to control cells (mean ± SD, $n$ = 5). ***$p$ < 0.001 (two-tailed Student's t test). Control, cells transfected with Mission siRNA universal negative control.

(C) Western blot analysis of mitochondria isolated from HEK293 cells with native or siRNA-depleted TIMM23 and TIMM17A. The quantification of protein signals from lanes that were loaded with 30 µg mitochondria is presented as mean ± SEM ($n$ = 3). *$p$ < 0.05 (two-tailed Student's t test).

(D) Western blot analysis of mitochondria isolated from TIMM17B WT/Del and wild-type HEK293 cells.

(E) Protein complexes in mitochondria isolated from HEK293 cells with native or siRNA-depleted TIMM23 and TIMM17A, solubilized with 1% digitonin-containing buffer, and resolved in 3%–13% BN-PAGE gel, followed by western blot. #, unspecific band.

(F) Protein complexes in mitochondria that were isolated from TIMM17B WT/Del and wild-type (WT) HEK293 cells, solubilized with 1% digitonin-containing buffer, and resolved in 6%–16% BN-PAGE gels, followed by western blot.

(G) Schema of the prohibitin complex and OCIAD1 control over the TIM23 complex biogenesis. Prohibitin complex regulates the biogenesis of TIMM17A- and TIMM17B-containing variants of the TIM23 translocase. OCIAD1 specifically protects the TIMM17A-containing variant from degradation by the YME1L protease. The absence of the TIMM17B-containing variant of the TIM23 translocase positively regulates OCIAD1.

See also Figure S10.

whether the whole TIMM17A-containing TIM23 complex or individual subunits (such as TIMM17A) are degraded in response to decreasing levels of OCIAD1. Our experiments demonstrate the interactions between OCIAD1, the prohibitin complex, and the TIM23 protein translocase, but the mechanism of these interactions, including their sequence, remains elusive. In particular, it remains to be elucidated whether the entire triad can form a ternary complex. In addition, we analyzed interactions between OCIAD1, the prohibitin complex, and TIM23 translocase using tagged protein bait, a commonly used approach, which, however, generally bears a risk, such as inefficient assembly of tagged proteins into their target complexes. An alternative strategy could involve affinity purification with antibodies against native proteins.

## RESOURCE AVAILABILITY

### Lead contact

Further information and requests for resources and reagents should be directed to and will be fulfilled by the lead contact, Agnieszka Chacinska (a.chacinska@imol.institute).

### Materials availability

Plasmids and cell lines generated in this study may be requested from the lead contact.

## Data and code availability

Source data are provided with this paper. The mass spectrometry data of $OCIAD1_{FLAG}$ affinity purification have been deposited in the ProteomeXchange Consortium[44] via the PRIDE[45] partner repository with the dataset identifier PRIDE: PXD043863. This paper does not report original code. Any additional information required to reanalyze the data reported in this paper is available from the lead contact upon request.

## ACKNOWLEDGMENTS

We thank Sven Dennerlein and Peter Rehling for sharing the HEK293T cell lines and scientific discussions. We thank Tomasz Banach for assistance with mass spectrometry sample preparation. We thank Vanessa Linke for the scientific discussions. The work was funded by the "Regenerative Mechanisms for Health - ReMedy" project (MAB/2017/2) carried out within the International Research Agendas Program of the Foundation for Polish Science co-financed by the European Union under the European Regional Development Fund; the National Science Centre, Poland (2015/19/B/NZ3/03272, 2019/35/B/NZ3/04095, 2019/35/B/NZ3/04066, and 2019/33/B/NZ5/00780); the European Molecular Biology Organization (ALTF 388-2023); and the Ministry of Education and Science, Poland (2/0050/DIA/2017/46). S.J. acknowledges support by the European Research Council (ERCAdG no. 835102) and the DFG-funded FOR2848 (project P04).

## AUTHOR CONTRIBUTIONS

A.C.,M.W., and P.E. conceived the study. P.E., K.K.M., M.W., A.S.G., M.A.B.-L., and S.N. performed the biochemical experiments. T.S. performed the microscopy experiments. P.E. and R.A.S. performed the proteomics experiments. A.C., S.J., M.W., P.E., K.K.M., R.A.S., and T.S. analyzed the data. P.E., M.W., K.K.M., and A.C. wrote the paper with input from all authors. A.G. generated the OCIAD1-KO and TIMM17B WT/Del cell lines.

## DECLARATION OF INTERESTS

The authors declare no competing interests.

## STAR★METHODS

Detailed methods are provided in the online version of this paper and include the following:

- KEY RESOURCES TABLE
- EXPERIMENTAL MODEL AND STUDY PARTICIPANT DETAILS
- METHOD DETAILS
  - Cell lines and growth conditions
  - Cloning of HA-TIMM17A, HA-TIMM17B, HA-OCIAD1, OCIAD1-HA, PHB2-HA into pcDNA 3.1(+)
  - Mitochondrial isolation by limited swelling
  - Subcellular fractionation
  - Mitoplasting
  - Sodium carbonate extraction
  - Cellular protein extract
  - siRNA mediated knock-down
  - FLAG tag affinity purification
  - HA-tag affinity purification
  - *In organello* import assay
  - Blue Native-PAGE
  - Quantitative real-time PCR
  - Mitochondrial membrane electrochemical potential measurement
  - Sample preparation for live-cell STED microscopy
  - STED microscopy
  - Sample preparation for fluorescence microscopy
  - Mass spectrometry
- QUANTIFICATION AND STATISTICAL ANALYSIS

## SUPPLEMENTAL INFORMATION

Supplemental information can be found online at https://doi.org/10.1016/j.celrep.2024.115038.

Received: October 12, 2023
Revised: September 27, 2024
Accepted: November 15, 2024
Published: December 3, 2024

## REFERENCES

1. Chacinska, A., Koehler, C.M., Milenkovic, D., Lithgow, T., and Pfanner, N. (2009). Importing Mitochondrial Proteins: Machineries and Mechanisms. Cell *138*, 628–644. https://doi.org/10.1016/j.cell.2009.08.005.
2. Neupert, W., and Herrmann, J.M. (2007). Translocation of proteins into mitochondria. Annu. Rev. Biochem. *76*, 723–749. https://doi.org/10.1146/annurev.biochem.76.052705.163409.
3. Busch, J.D., Fielden, L.F., Pfanner, N., and Wiedemann, N. (2023). Mitochondrial protein transport: Versatility of translocases and mechanisms. Mol. Cell *83*, 890–910. https://doi.org/10.1016/j.molcel.2023.02.020.
4. Schulz, C., Schendzielorz, A., and Rehling, P. (2015). Unlocking the presequence import pathway. Trends Cell Biol. *25*, 265–275. https://doi.org/10.1016/j.tcb.2014.12.001.
5. Callegari, S., Cruz-Zaragoza, L.D., and Rehling, P. (2020). From TOM to the TIM23 complex - handing over of a precursor. Biol. Chem. *401*, 709–721. https://doi.org/10.1515/hsz-2020-0101.
6. Popov-Čeleketić, D., Mapa, K., Neupert, W., and Mokranjac, D. (2008). Active remodelling of the TIM23 complex during translocation of preproteins into mitochondria. EMBO J. *27*, 1469–1480. https://doi.org/10.1038/emboj.2008.79.
7. van der Laan, M., Meinecke, M., Dudek, J., Hutu, D.P., Lind, M., Perschil, I., Guiard, B., Wagner, R., Pfanner, N., and Rehling, P. (2007). Motor-free mitochondrial presequence translocase drives membrane integration of preproteins. Nat. Cell Biol. *9*, 1152–1159. https://doi.org/10.1038/ncb1635.
8. Chacinska, A., Lind, M., Frazier, A.E., Dudek, J., Meisinger, C., Geissler, A., Sickmann, A., Meyer, H.E., Truscott, K.N., Guiard, B., et al. (2005). Mitochondrial Presequence Translocase: Switching between TOM Tethering and Motor Recruitment Involves Tim21 and Tim17. Cell *120*, 817–829. https://doi.org/10.1016/j.cell.2005.01.011.
9. Sim, S.I., Chen, Y., Lynch, D.L., Gumbart, J.C., and Park, E. (2023). Structural basis of mitochondrial protein import by the TIM23 complex. Nature *621*, 620–626. https://doi.org/10.1038/s41586-023-06239-6.
10. Fielden, L.F., Busch, J.D., Merkt, S.G., Ganesan, I., Steiert, C., Hasselblatt, H.B., Busto, J.V., Wirth, C., Zufall, N., Jungbluth, S., et al. (2023). Central role of Tim17 in mitochondrial presequence protein translocation. Nature *621*, 627–634. https://doi.org/10.1038/s41586-023-06477-8.
11. Zhou, X., Yang, Y., Wang, G., Wang, S., Sun, D., Ou, X., Lian, Y., and Li, L. (2023). Molecular pathway of mitochondrial preprotein import through the TOM–TIM23 supercomplex. Nat. Struct. Mol. Biol. *30*, 1996–2008. https://doi.org/10.1038/s41594-023-01103-7.
12. Wrobel, L., Sokol, A.M., Chojnacka, M., and Chacinska, A. (2016). The presence of disulfide bonds reveals an evolutionarily conserved mechanism involved in mitochondrial protein translocase assembly. Sci. Rep. *6*, 27484. https://doi.org/10.1038/srep27484.
13. Ramesh, A., Peleh, V., Martinez-Caballero, S., Wollweber, F., Sommer, F., van der Laan, M., Schroda, M., Alexander, R.T., Campo, M.L., and Herrmann, J.M. (2016). A disulfide bond in the TIM23 complex is crucial for voltage gating and mitochondrial protein import. J. Cell Biol. *214*, 417–431. https://doi.org/10.1083/jcb.201602074.
14. Sinha, D., Srivastava, S., Krishna, L., and D'Silva, P. (2014). Unraveling the intricate organization of mammalian mitochondrial presequence translocases: existence of multiple translocases for maintenance of

CellPress
OPEN ACCESS

mitochondrial function. Mol. Cell Biol. *34*, 1757–1775. https://doi.org/10.1128/MCB.01527-13.

15. Rainbolt, T.K., Atanassova, N., Genereux, J.C., and Wiseman, R.L. (2013). Stress-regulated translational attenuation adapts mitochondrial protein import through Tim17A degradation. Cell Metab. *18*, 908–919. https://doi.org/10.1016/j.cmet.2013.11.006.
16. MacVicar, T., Ohba, Y., Nolte, H., Mayer, F.C., Tatsuta, T., Sprenger, H.-G., Lindner, B., Zhao, Y., Li, J., Bruns, C., et al. (2019). Lipid signalling drives proteolytic rewiring of mitochondria by YME1L. Nature *575*, 361–365. https://doi.org/10.1038/s41586-019-1738-6.
17. Morgenstern, M., Peikert, C.D., Lübbert, P., Suppanz, I., Klemm, C., Alka, O., Steiert, C., Naumenko, N., Schendzielorz, A., Melchionda, L., et al. (2021). Quantitative high-confidence human mitochondrial proteome and its dynamics in cellular context. Cell Metab. *33*, 2464–2483.e18. https://doi.org/10.1016/j.cmet.2021.11.001.
18. Palmer, C.S., Lou, J., Kouskousis, B., Pandzic, E., Anderson, A.J., Kang, Y., Hinde, E., and Stojanovski, D. (2021). Super-resolution microscopy reveals the arrangement of inner membrane protein complexes in mammalian mitochondria. J. Cell Sci. *134*, jcs252197. https://doi.org/10.1242/jcs.252197.
19. Sinha, A., Khadilkar, R.J., RoyChowdhury Sinha, A., Roychowdhury Sinha, A., and Inamdar, M.S. (2013). Conserved Regulation of the JAK/STAT Pathway by the Endosomal Protein Asrij Maintains Stem Cell Potency. Cell Rep. *4*, 649–658. https://doi.org/10.1016/j.celrep.2013.07.029.
20. Shetty, D.K., Kalamkar, K.P., and Inamdar, M.S. (2018). OCIAD1 Controls Electron Transport Chain Complex I Activity to Regulate Energy Metabolism in Human Pluripotent Stem Cells. Stem Cell Rep. *11*, 128–141. https://doi.org/10.1016/j.stemcr.2018.05.015.
21. Motiwala, Z., Darne, P., Prabhune, A., Inamdar, M.S., and Kulkarni, K. (2021). Expression, Purification and Crystallization of Asrij, A Novel Scaffold Transmembrane Protein. J. Membr. Biol. *254*, 65–74. https://doi.org/10.1007/s00232-020-00166-7.
22. Antonicka, H., Lin, Z.-Y., Janer, A., Aaltonen, M.J., Weraarpachai, W., Gingras, A.-C., and Shoubridge, E.A. (2020). A High-Density Human Mitochondrial Proximity Interaction Network. Cell Metab. *32*, 479–497.e9. https://doi.org/10.1016/j.cmet.2020.07.017.
23. Le Vasseur, M., Friedman, J., Jost, M., Xu, J., Yamada, J., Kampmann, M., Horlbeck, M.A., Salemi, M.R., Phinney, B.S., Weissman, J.S., and Nunnari, J. (2021). Genome-wide CRISPRi screening identifies OCIAD1 as a prohibitin client and regulatory determinant of mitochondrial Complex III assembly in human cells. Elife *10*, e67624. https://doi.org/10.7554/eLife.67624.
24. Lukinavičius, G., Umezawa, K., Olivier, N., Honigmann, A., Yang, G., Plass, T., Mueller, V., Reymond, L., Corrêa, I.R., Luo, Z.-G., et al. (2013). A near-infrared fluorophore for live-cell super-resolution microscopy of cellular proteins. Nat. Chem. *5*, 132–139. https://doi.org/10.1038/nchem.1546.
25. Liu, T., Stephan, T., Chen, P., Keller-Findeisen, J., Chen, J., Riedel, D., Yang, Z., Jakobs, S., and Chen, Z. (2022). Multi-color live-cell STED nanoscopy of mitochondria with a gentle inner membrane stain. Proc. Natl. Acad. Sci. USA *119*, e2215799119. https://doi.org/10.1073/pnas.2215799119.
26. Hallgren, J., Tsirigos, K.D., Pedersen, M.D., Armenteros, J.J.A., Marcatili, P., Nielsen, H., Krogh, A., and Winther, O. (2022). DeepTMHMM predicts alpha and beta transmembrane proteins using deep neural networks. Preprint at: bioRxiv. https://doi.org/10.1101/2022.04.08.487609
27. Richter-Dennerlein, R., Korwitz, A., Haag, M., Tatsuta, T., Dargazanli, S., Baker, M., Decker, T., Lamkemeyer, T., Rugarli, E.I., and Langer, T. (2014). DNAJC19, a mitochondrial cochaperone associated with cardiomyopathy, forms a complex with prohibitins to regulate cardiolipin remodeling. Cell Metab. *20*, 158–171. https://doi.org/10.1016/j.cmet.2014.04.016.
28. Liu, F., Lössl, P., Rabbitts, B.M., Balaban, R.S., and Heck, A.J.R. (2018). The interactome of intact mitochondria by cross-linking mass spectrometry provides evidence for coexisting respiratory supercomplexes. Mol. Cell. Proteom. *17*, 216–232. https://doi.org/10.1074/mcp.RA117.000470.
29. Yoshinaka, T., Kosako, H., Yoshizumi, T., Furukawa, R., Hirano, Y., Kuge, O., Tamada, T., and Koshiba, T. (2019). Structural Basis of Mitochondrial Scaffolds by Prohibitin Complexes: Insight into a Role of the Coiled-Coil Region. iScience *19*, 1065–1078. https://doi.org/10.1016/j.isci.2019.08.056.
30. Zhu, Y., Akkaya, K.C., Ruta, J., Yokoyama, N., Wang, C., Ruwolt, M., Lima, D.B., Lehmann, M., and Liu, F. (2024). Cross-link assisted spatial proteomics to map sub-organelle proteomes and membrane protein topologies. Nat. Commun. *15*, 3290. https://doi.org/10.1038/s41467-024-47569-x.
31. Tatsuta, T., and Langer, T. (2017). Prohibitins. Curr. Biol. *27*, R629–R631. https://doi.org/10.1016/j.cub.2017.04.030.
32. Richter, F., Dennerlein, S., Nikolov, M., Jans, D.C., Naumenko, N., Aich, A., MacVicar, T., Linden, A., Jakobs, S., Urlaub, H., et al. (2019). ROMO1 is a constituent of the human presequence translocase required for YME1L protease import. J. Cell Biol. *218*, 598–614. https://doi.org/10.1083/jcb.201806093.
33. Wenz, L.-S., Opaliński, Ł., Wiedemann, N., and Becker, T. (2015). Cooperation of protein machineries in mitochondrial protein sorting. Biochim. Biophys. Acta - Mol. Cell Res. *1853*, 1119–1129. https://doi.org/10.1016/j.bbamcr.2015.01.012.
34. Sinzel, M., Tan, T., Wendling, P., Kalbacher, H., Özbalci, C., Chelius, X., Westermann, B., Brügger, B., Rapaport, D., and Dimmer, K.S. (2016). Mcp3 is a novel mitochondrial outer membrane protein that follows a unique IMP-dependent biogenesis pathway. EMBO Rep. *17*, 965–981. https://doi.org/10.15252/embr.201541273.
35. Osman, C., Merkwirth, C., and Langer, T. (2009). Prohibitins and the functional compartmentalization of mitochondrial membranes. J. Cell Sci. *122*, 3823–3830. https://doi.org/10.1242/jcs.037655.
36. Qiao, Z., Yokoyama, T., Yan, X.-F., Beh, I.T., Shi, J., Basak, S., Akiyama, Y., and Gao, Y.-G. (2022). Cryo-EM structure of the entire FtsH-HflKC AAA protease complex. Cell Rep. *39*, 110890. https://doi.org/10.1016/j.celrep.2022.110890.
37. Tatsuta, T., Model, K., and Langer, T. (2005). Formation of membrane-bound ring complexes by prohibitins in mitochondria. Mol. Biol. Cell *16*, 248–259. https://doi.org/10.1091/mbc.e04-09-0807.
38. Lange, F., Ratz, M., Dohrke, J.-N., Wenzel, D., Ilgen, P., Riedel, D., and Jakobs, S. (2024). In-situ architecture of the human prohibitin complex. Preprint at: bioRxiv. https://doi.org/10.1101/2024.02.14.579514
39. Da Cruz, S., Parone, P.A., Gonzalo, P., Bienvenut, W.V., Tondera, D., Jourdain, A., Quadroni, M., and Martinou, J.-C. (2008). SLP-2 interacts with prohibitins in the mitochondrial inner membrane and contributes to their stability. Biochim. Biophys. Acta *1783*, 904–911. https://doi.org/10.1016/j.bbamcr.2008.02.006.
40. Wai, T., Saita, S., Nolte, H., Müller, S., König, T., Richter-Dennerlein, R., Sprenger, H.-G., Madrenas, J., Mühlmeister, M., Brandt, U., et al. (2016). The membrane scaffold SLP2 anchors a proteolytic hub in mitochondria containing PARL and the i-AAA protease YME1L. EMBO Rep. *17*, 1844–1856. https://doi.org/10.15252/embr.201642698.
41. Mitsopoulos, P., Chang, Y.-H., Wai, T., König, T., Dunn, S.D., Langer, T., and Madrenas, J. (2015). Stomatin-Like Protein 2 Is Required for *In Vivo* Mitochondrial Respiratory Chain Supercomplex Formation and Optimal Cell Function. Mol. Cell Biol. *35*, 1838–1847. https://doi.org/10.1128/MCB.00047-15.
42. Steglich, G., Neupert, W., and Langer, T. (1999). Prohibitins regulate membrane protein degradation by the m-AAA protease in mitochondria. Mol. Cell Biol. *19*, 3435–3442. https://doi.org/10.1128/MCB.19.5.3435.
43. Nijtmans, L.G., de Jong, L., Artal Sanz, M., Coates, P.J., Berden, J.A., Back, J.W., Muijsers, A.O., van der Spek, H., and Grivell, L.A. (2000).

Prohibitins act as a membrane-bound chaperone for the stabilization of mitochondrial proteins. EMBO J. *19*, 2444–2451. https://doi.org/10.1093/emboj/19.11.2444.

44. Deutsch, E.W., Bandeira, N., Perez-Riverol, Y., Sharma, V., Carver, J.J., Mendoza, L., Kundu, D.J., Wang, S., Bandla, C., Kamatchinathan, S., et al. (2023). The ProteomeXchange consortium at 10 years: 2023 update. Nucleic Acids Res. *51*, D1539–D1548. https://doi.org/10.1093/nar/gkac1040.

45. Perez-Riverol, Y., Bai, J., Bandla, C., García-Seisdedos, D., Hewapathirana, S., Kamatchinathan, S., Kundu, D.J., Prakash, A., Frericks-Zipper, A., Eisenacher, M., et al. (2022). The PRIDE database resources in 2022: a hub for mass spectrometry-based proteomics evidences. Nucleic Acids Res. *50*, D543–D552. https://doi.org/10.1093/nar/gkab1038.

46. Ran, F.A., Hsu, P.D., Wright, J., Agarwala, V., Scott, D.A., and Zhang, F. (2013). Genome engineering using the CRISPR-Cas9 system. Nat. Protoc. *8*, 2281–2308. https://doi.org/10.1038/nprot.2013.143.

47. Panov, A. (2013). Practical Mitochondriology: Pitfalls and Problems in Studies of Mitochondria (CreateSpace Independent Publishing Platform).

48. Myers, S.A., Rhoads, A., Cocco, A.R., Peckner, R., Haber, A.L., Schweitzer, L.D., Krug, K., Mani, D.R., Clauser, K.R., Rozenblatt-Rosen, O., et al. (2019). Streamlined protocol for deep proteomic profiling of FAC-sorted cells and its application to freshly isolated murine immune cells. Mol. Cell. Proteomics *18*, 995–1009.

49. Tyanova, S., Temu, T., Sinitcyn, P., Carlson, A., Hein, M.Y., Geiger, T., Mann, M., and Cox, J. (2016). The Perseus computational platform for comprehensive analysis of (prote)omics data. Nat. Methods *13*, 731–740. https://doi.org/10.1038/nmeth.3901.

## STAR★METHODS

### KEY RESOURCES TABLE

| REAGENT or RESOURCE | SOURCE | IDENTIFIER |
|---|---|---|
| Antibodies | | |
| ACO2 | Cell Signaling Technology | Cat# 6571; RRID:AB_2797630 |
| AIF | Santa Cruz Biotechnology | Cat# sc-13116; RRID:AB_626654 |
| ALR | Santa Cruz Biotechnology | Cat# sc-134869; RRID:AB_10608344 |
| ATPB | Abcam | Cat# ab5432; RRID:AB_304883 |
| ATP5A | Abcam | Cat# ab14748; RRID:AB_301447 |
| ATP5B | Rabbit Serum | In-house produced, a gift from Prof. P. Rehling |
| ANT2 | Cell Signaling Technology | Cat# 14671; RRID:AB_2798562 |
| COA7 | Sigma-Aldrich | Cat# HPA029926; RRID:AB_10602173 |
| COX6A | 3282.7 | In-house produced, a gift from Prof. P. Rehling |
| COX4 | Cell Signaling Technology | Cat# 4850; RRID:AB_2085424 |
| CYCS | Abcam | Cat# ab133504; RRID:AB_2802115 |
| DNAJC19 | Proteintech | Cat# 12096-1-AP; RRID:AB_2094914 |
| FLAG tag | Sigma-Aldrich | Cat# F3165; RRID:AB_259529 |
| FLAG tag | Thermo Fisher Scientific | Cat# PA1-984B; RRID:AB_347227 |
| HA-Tag | Cell Signaling Technology | Cat# 3724 (also 3724S); RRID:AB_1549585 |
| MRPL55 | Proteintech | Cat# 17679-1-AP; RRID:AB_2878427 |
| MIC60 | Abcam | Cat# ab137057 |
| Mitofusin-1 | Cell Signaling Technology | Cat# 14739; RRID:AB_2744531 |
| OCIAD1 | GeneTex | Cat# GTX85234; RRID:AB_10734387 |
| PHB1 | Cell Signaling Technology | Cat# 2426; RRID:AB_823689 |
| PHB2 | Cell Signaling Technology | Cat# 14085; RRID:AB_2798387 |
| TIMM21 | Rabbit Serum | In-house produced, a gift from Prof. P. Rehling |
| TIMM22 | Proteintech | Cat# 14927-1-AP; RRID:AB_11183050 |
| TIMM23 | BD Biosciences | Cat# 611222; RRID:AB_398754 |
| TIMM17A | Abcam | Cat# ab192246 |
| TIMM17B | Thermo Fisher Scientific | Cat# PA5-56054; RRID:AB_2648475 |
| TIMM44 | Proteintech | Cat# 13859-1-AP; RRID:AB_2204679 |
| TOMM20 | Santa Cruz Biotechnology | Cat# sc-11415; RRID:AB_2207533 |
| TOMM40 | Santa Cruz Biotechnology | Cat# sc-365467; RRID:AB_10847086 |
| TOMM70 | Atlas Antibodies | Cat# HPA048020; RRID:AB_2680234 |
| Tubulin | Santa Cruz Biotechnology | Cat# sc-134239; RRID:AB_2210215 |
| SAMM50 | Abcam | Cat# ab167430 |
| SDHA | Santa Cruz Biotechnology | Cat# sc-166947; RRID:AB_10610526 |
| SDHC | Abcam | Cat# ab155999; RRID:AB_2810989 |
| UQCRC1 | Sigma-Aldrich | Cat# HPA002815; RRID:AB_1080486 |
| VDAC1 | Abcam | Cat# ab15895; RRID:AB_2214787 |
| YME1L | Proteintech | Cat# 11510-1-AP; RRID:AB_2217459 |
| Chemicals, peptides, and recombinant proteins | | |
| jetPRIME® Polyplus transfection | Polyplus | 114 |

(*Continued on next page*)

*Continued*

| REAGENT or RESOURCE | SOURCE | IDENTIFIER |
|---|---|---|
| TNT SP6 Quick Coupled Transcription/Translation System | Promega | L2080 |
| [$^{35}$S]Methionine | PerkinElmer | NEG009T005MC |
| TMRM | Thermo Fisher Scientific | M20036 |
| Digitonin | Calbiochem | 300410 |
| Calibration Kit High Molecular Weight For Electrophoresis | Cytvia | GE17-0445-01 |
| Lipofectamine™ RNAiMAX Transfection Reagent | Thermo Fisher Scientific | 13778150 |
| Mission siRNA universal negative control | Sigma | SIC001 |
| FLAG® Peptide | Sigma | F3290 |
| Pierce™ Anti-HA Agarose | Thermo Fisher Scientific | 26182 |
| anti-FLAG M2 affinity gel | Sigma | A2220 |
| TMT10plex™ Isobaric Label Reagent | Thermo Fisher Scientific | 90110 |
| **Critical commercial assays** | | |
| RNeasy Plus Mini Kit | Qiagen | 74134 |
| SuperScript™ IV First-Strand Synthesis System | Thermo Fisher Scientific | 18091050 |
| Maxima First Strand cDNA Synthesis Kit | Thermo Fisher Scientific | K1671 |
| SensiFAST™ SYBR® Hi-ROX Kit | Bioline | BIO-92020 |
| **Deposited data** | | |
| The mass spectrometry data of OCIAD1FLAG affinity purification | ProteomeXchange Consortium | PXD043863 |
| **Experimental models: Cell lines** | | |
| HEK293 cell line | American Type Culture Collection (ATCC) | – |
| HEK293 OCIAD1-KO cell line | This Paper | NA |
| HEK293 TIMM17B WT/Del cell line | This Paper | NA |
| HEK293T cell line | Richter et al.2019[32] | NA |
| HEK293T-TIMM23$_{FLAG}$ cell line | Richter et al.2019[32] | NA |
| HEK293T-TIMM21$_{FLAG}$ | Richter et al.2019[32] | NA |
| **Oligonucleotides** | | |
| OCIAD1-F (for cloning guide RNA in pX330 vector) | 5′CACC(G)TTTTCGAGAGCCGAATGCAG3′ | This Paper |
| OCIAD1-R (for cloning guide RNA in pX330 vector) | 5′AAACCTGCATTCGGCTCTCGAAAA(C)3′ | This Paper |
| TIMM17B-F (for cloning guide RNA in pX330 vector) | 5′CACC(G)TTCACTATGGGTGTCATCGG3′ | This Paper |
| TIMM17B-R (for cloning guide RNA in pX330 vector) | 5′AAACCCGATGACACCCATAGTGAAC3′ | This Paper |
| TIMM17A-F (for cloning guide RNA in pTNT vector) | 5′AAAACTCGAGGCCACCATGGA GGAGTACGCGCGAGAGC 3′ | This Paper |
| TIMM17A-R (for cloning guide RNA in pTNT vector) | 5′AAAAGCGGCCGCCTACTGATA TTGTCGATAGTCT3′ | This Paper |
| TIMM17B-F (for cloning guide RNA in pTNT vector) | 5′AAAACTCGAGGCCACCATGGA GGAGTACGCTCGGGAGC3′ | This Paper |
| TIMM17B-R (for cloning guide RNA in pTNT vector) | 5′AAAAGCGGCCGCTCAGTGGTA CTGCTGATAGCTG3′ | This Paper |
| OCIAD1 sense strand (siRNA Knockdown) | 5′GAGCCAAUUCCAUUCAGUU3′ | This Paper |
| OCIAD1 anti-sense strand (siRNA Knockdown) | 5′ACUGAAUGGAAUUGGCUC3′ | This Paper |

(*Continued on next page*)

***Continued***

| REAGENT or RESOURCE | SOURCE | IDENTIFIER |
|---|---|---|
| TIMM22 sense strand (siRNA Knockdown) | 5′GUGAGGAGCAGAAGAUGAU3′ | This Paper |
| TIMM22 anti-sense strand (siRNA Knockdown) | 5′AUCAUCUUCUGCUCCUCAC3′ | This Paper |
| TIMM23 sense strand (siRNA Knockdown) | 5′CCCUCUGUCUCCUUAUUUA3′ | This Paper |
| TIMM23 anti-sense strand (siRNA Knockdown) | 5′UAAAUAAGGAGACAGAGGG3′ | This Paper |
| TIMM17A-1 sense strand (siRNA Knockdown) | 5′AGACUAUCGACAAUAUCAGUAGGAC3′ | This Paper |
| TIMM17A-1 anti-sense strand (siRNA Knockdown) | 5′GUCCUACUGAUAUUGU CGAUAGUCUCC3′ | This Paper |
| TIMM17A-2 sense strand (siRNA Knockdown) | 5′CCAAUAUACCCAAUUCUAUAUGAAG3′ | This Paper |
| TIMM17A-2 anti-sense strand (siRNA Knockdown) | 5′CUUCAUAUAGAAUUGGGUAUAUUGGAA3′ | This Paper |
| PHB2 sense strand (siRNA Knockdown) | 5′AGAUUCGAGCAGCCCAGAAUAUCTC3′ | This Paper |
| PHB2 anti-sense strand (siRNA Knockdown) | 5′GUUCUAAGCUCGUCGGGUCUUAUAGAG3′ | This Paper |
| HA-TIMM17A_Forward (to clone *TIMM17A* cDNA sequence into pcDNA 3.1 (+), contains HA at 5′) | 5′aggccggatccATGTACCCATACGATG TTCCAGATTACGCTgaggagtacgcgcgagagcc 3′ | This paper |
| TIMM17A_Reverse (to clone *TIMM17A* cDNA sequence into pcDNA 3.1 (+)) | 5′ggcctctcgagctactgatattgtcgatagtctcc – 3′ | This paper |
| HA-TIMM17B_Forward (to clone *TIMM17B* cDNA sequence into pcDNA 3.1 (+), contains HA at 5′) | 5′aggccaagcttATGTACCCATACG ATGTTCCAGATTACGCTgaggagt acgctcgggagc 3′ | This paper |
| TIMM17B_Reverse (to clone *TIMM17B* cDNA sequence into pcDNA 3.1 (+)) | 5′ Ggcctctcgagtcagtggtactgctgatagctg 3′ | This paper |
| HA-OCIAD1_Forward (to clone *OCIAD1* cDNA sequence into pcDNA 3.1 (+), contains HA at 5′) | 5′aggccggatccATGTACCCATACGATGTTC CAGATTACGCTaatgggagggctgattttcgag 3′ | This paper |
| OCIAD1_Reverse (to clone *OCIAD1* cDNA sequence into pcDNA 3.1 (+)) | 5′ggcctctcgagtcactcatcccaagtatctcc 3′ | This paper |
| OCIAD1_Forward (to clone *OCIAD1* cDNA sequence into pcDNA 3.1 (+)) | 5′aggccggatccATGaatgggag ggctgattttcgag 3′ | This paper |
| OCIAD1-HA_Reverse (to clone *OCIAD1* cDNA sequence into pcDNA 3.1 (+), contains HA at 3′) | 5′ggcctctcgagtcaAGCGTAATCTGGAACAT CGTATGGGTActcatcccaagtatctccatac 3′ | This paper |
| Prohibitin 2_Forward (to clone *PHB2 cDNA* sequence into pcDNA 3.1 (+)) | 5′aggccaagcttATGgcccagaa cttgaaggacttgg 3′ | This paper |
| Prohibitin 2-HA_Reverse (to clone *PHB2 cDNA* sequence into pCDNA 3.1 (+), contains HA at 3′) | 5′ggcctctcgagtcaAGCGTAATCTGGAACATC GTATGGGTAtttcttacccttgatgagg 3′ | This paper |
| TIMM17B_KD_Forward | 5′CUCUAUCACCAGUGGAGCA 3′ | This paper |
| TIMM17B_KD_Reverse | 5′UGCUCCACUGGUGAUAGAG 3′ | This paper |
| Hs_TIMM17A_5 FlexiTube siRNA | Qiagen | GeneGlobe ID - SI03129042 |

(*Continued on next page*)

*Continued*

| REAGENT or RESOURCE | SOURCE | IDENTIFIER |
|---|---|---|
| Recombinant DNA | | |
| pEGFP-N1 | Clontech Laboratories, Inc | N/A |
| pX330 vector | Zhang Lab, Addgene | #42230 |
| pcDNA3.1(+) | ThermoFisher Scientific | V79020 |
| OCIAD1$_{FLAG}$ (pcDNA3.1(+)) | This paper | N/A |
| OCIAD1$_{SNAP}$ (pcDNA3.1(+)) | This paper | N/A |
| COX4(pTNT) | This paper | N/A |
| OTC(pTNT) | This paper | N/A |
| TIMM23(pTNT) | This paper | N/A |
| ANT3(pTNT) | This paper | N/A |
| HA-TIMM17A (pcDNA 3.1 (+)) | This paper | N/A |
| HA-TIMM17B (pcDNA 3.1 (+)) | This paper | N/A |
| HA-OCIAD1 (pcDNA 3.1 (+)) | This paper | N/A |
| OCIAD1-HA (pcDNA 3.1 (+)) | This paper | N/A |
| PHB2-HA (pcDNA 3.1 (+)) | This paper | N/A |
| TIMM17A (pTNT) | This paper | N/A |
| TIMM17B (pTNT) | This paper | N/A |
| Software and algorithms | | |
| Adobe Photoshop Elements | Adobe Systems | https://www.adobe.com |
| Adobe Illustrator | Adobe Inc. | https://www.adobe.com |
| Graphpad | Graphpad Software, LLC | https://www.graphpad.com |
| ImageJ | NIH | https://imagej.net/ij |
| MaxQuant v. 1.6.7.0 | Max Plank Institute of Biochemistry | https://www.maxquant.org |

## EXPERIMENTAL MODEL AND STUDY PARTICIPANT DETAILS

The cell lines used in the study are HEK293 wildtype, HEK293 OCIAD1-KO, HEK293 TIMM17B WT/Del, HEK293T wildtype, HEK293T TIMM23$_{FLAG}$, and HEK293T TIMM21$_{FLAG}$. They are listed in the key resources table and were cultivated as mentioned in the methods section.

## METHOD DETAILS

### Cell lines and growth conditions

Human Embryonic Kidney (HEK293) and HeLa cell lines were purchased from American Type Culture Collection (ATCC). The HEK293 OCIAD1-KO and TIMM17B WT/Del cell lines were generated using CRISPR/Cas9 technology as previously described.[46] The gene guide RNA was designed using software provided by Prof. Feng Zhang's laboratory. The selected guide was cloned into the pX330 vector using the following oligos: OCIAD1 (5′CACC(G)TTTTCGAGAGCCGAATGCAG3′, 5′AAACCTGCATTCGGCTCTCGAAAA(C)3′) and TIMM17B 5′CACC(G)TTCACTATGGGTGTCATCGG3′, 5′AAACCCGATGACACCCATAGTGAAC3′). The pX330 vector was co-transfected with an EGFP-containing plasmid (pEGFP-N1; Clontech Laboratories, Inc) into HEK293 cells. The knockouts that survived Zeocin selection were validated by sequencing, SDS–PAGE, and western blotting after single cell sorting by FACS Vantage SE equipment. The TIMM17B western blots presented in this manuscript correspond to a shorter isoform of TIMM17B (NCBI accession no. NP_005825.1, ~20 kDa). The HEK293T cell lines (HEK293T, HEK293T-TIMM23FLAG and HEK293T-TIMM21FLAG) engineered for cloning and tetracycline-controlled expression of genes were a kind gift from Peter Rehling, University of Gottingen, Germany. The cells were grown at 37°C with 5% CO2 in Dulbecco's modified Eagle medium (DMEM) containing high glucose (4.5 g/L), supplemented with 10% (v/v) fetal bovine serum, 2 mM L-glutamine, 100 U/ml penicillin, and 100 μg/mL streptomycin. U-2 OS cells (ATCC) were cultured in McCoy's medium (Thermo Fisher Scientific). Culture media were supplemented with 100 μg/mL streptomycin (Merck Millipore), 1 mM sodium pyruvate (Sigma-Aldrich), and 10% (v/v) fetal bovine serum (Merck Millipore). The growth rate of OCIAD1-KO cells was similar to the wild-type HEK293 cells. For overexpression of the protein, the plasmid pcDNA3.1(+) containing the gene of interest was transfected using jetPRIME polyplus transfection reagent as per manufacturer's protocol for 48 or 72 h.

### Cloning of HA-TIMM17A, HA-TIMM17B, HA-OCIAD1, OCIAD1-HA, PHB2-HA into pcDNA 3.1(+)

DNA sequences encoding TIMM17A and TIMM17B were synthetized by BioCat (Germany). Subsequently, TIMM17A and TIMM17B were cloned into pcDNA 3.1 (+) with N-terminally localized HA tag (TAC CCA TAC GAT GTT CCA GAT TAC GCT) between BamHI and XhoI and HindIII and XhoI restriction sites, respectively. DNA sequences encoding OCIAD1 and Prohibitin 2 were amplified directly from the cDNA isolated from HEK293T cells using primers listed in the key resources table. OCIAD1 was cloned either with N- or C-terminally localized HA tag into pcDNA 3.1 (+) between BamHI and XhoI restriction sites. Prohibitin 2 was cloned with C-terminally localized HA tag into pcDNA 3.1 (+) between HindIII and XhoI restriction sites.

### Mitochondrial isolation by limited swelling

The protocol of mitochondria isolation was adopted from.[47] Cells were grown in high glucose DMEM, harvested using a scraper and washed with 1X PBS by centrifugation (1000 x g, 5 min, 4°C). The washed cell pellet was resuspended in an ice-cold isotonic buffer (75 mM mannitol, 225 mM sucrose, 1 mM EGTA 10 mM MOPS-KOH, pH 7.2) containing 2 mM PMSF and centrifuged (1000 x g, 5 min, 4°C). Then, the cell pellet was resuspended in hypotonic buffer (100 mM sucrose, 1 mM EGTA 10 mM MOPS-KOH, pH 7.2) containing 2 mg/mL bovine serum albumin (BSA) and 2 mM PMSF and incubated on ice for 5–7 min. The cell suspension was homogenized with 15 strokes using a Dounce glass homogenizer (Sartorius, Catalog no. BBI-8540705). Cold hypertonic buffer (1.25 M sucrose, 10 mM MOPS-KOH, pH 7.2) was added to the cell homogenate suspension (1.1 mL/g cells), and the volume was doubled with isotonic buffer containing 2 mg/mL BSA and 2 mM PMSF. Next, the homogenate suspension was centrifuged (1,000 x *g*, 10 min, 4°C) and the supernatant containing mitochondria was centrifuged again to pellet the debris. Then, the supernatant was subjected to high-speed centrifugation (10,000 x *g*, 10 min, 4°C) to pellet mitochondria. The pellet was resuspended with ice-cold isotonic buffer (without BSA or PMSF) and centrifuged again. Lastly, the pellet was resuspended in isotonic buffer (without BSA or PMSF) and protein concentration was measured using the Bradford assay. Mitochondria were either used freshly or flash-frozen using liquid nitrogen and stored at −80°C for further usage.

### Subcellular fractionation

Subcellular fractionation was performed identical to the procedure for isolation of mitochondria until the addition of isotonic buffer post-homogenization, which in the case of subcellular fractionation was devoid of BSA. The suspension was then centrifuged (1,000 x *g*, 10 min, 4°C), and debris was discarded. The supernatant was separated into three equal portions. The first portion ("Total" fraction) was subjected to pyrogallol red precipitation to recover proteins. The second and third portions were centrifuged (20,000 x *g*, 10 min, 4°C). The second portion's supernatant was collected and subjected to pyrogallol red precipitation to recover all cytosolic proteins. The third portion's supernatant was subjected to ultracentrifugation (100,000 x g, 60 min, 4°C) to separate vesicles (pellet) from a cytosolic fraction (supernatant).

### Mitoplasting

The isolated mitochondria were placed in three different sucrose buffers: (1) 250 mM sucrose buffer (250mM sucrose, 20mM HEPES-KOH, pH 7.4), (2) 5 mM sucrose buffer (25mM sucrose, 20mM HEPES-KOH, pH 7.4), and (3) 1% Triton X-100 treated 250mM sucrose buffer and incubated on ice for 30 min. The solution was separated into two parts and treated with proteinase K (25 μg/mL) and incubated on ice for 5 min. Then, PMSF was added, and solutions were centrifuged (20,000 x *g*, 10 min, 4°C). Pyrogallol red precipitation was used to recover proteins from the supernatant. The pellet was washed with 100 μL sucrose buffer and centrifuged (20,000 x *g*, 10 min, 4°C). The washed pellet was solubilized with urea sample buffer with DTT (50 mM) and analyzed by SDS-PAGE and western blot.

### Sodium carbonate extraction

Isolated mitochondria (200 μg) were resuspended in 420 μL sodium carbonate solution (0.1 M, pH 10.8) and incubated on ice for 30 min. Then, the suspension was separated into two equal portions. One portion was ultra-centrifuged (100,000 x g, 60 min, 4°C), and the pellet (membrane fraction) was resuspended in 2x Laemmli buffer with 50 mM DTT. The other portion (total fraction) and the supernatant (soluble fraction) from ultracentrifugation were subjected to TCA precipitation, and the obtained pellet was resuspended in 2 x Laemmli buffer with 50 mM DTT. All samples were analyzed using SDS-PAGE and western blot.

### Cellular protein extract

After harvesting cells using a scraper, the cells were washed twice with 1X PBS by centrifugation (1,000 x g, 5 min, 4°C). Then, the cell pellet was solubilized in RIPA buffer (150 mM NaCl, 1% v/v NP–40, 0.25% sodium deoxycholate, 1 mM EDTA, 2 mM PMSF, 65 mM Tris-HCl, pH 7.4) for 30 min at 4°C. The post-nuclear supernatant was collected after the cell lysate was cleared by centrifugation (14,000 x g, 30min, 4°C). The protein concentration was measured using bicinchoninic acid (BCA) protein assay. The remaining fraction was solubilized in 2x Laemmli sample buffer with 50 mM DTT. For TIMM23 silenced cells: After harvesting cells using a scraper, the cells were washed twice with 1X PBS by centrifugation (1,000 x g, 5 min, 4°C). The cell pellet was resuspended in an ice-cold isotonic buffer (75 mM mannitol, 225 mM sucrose, 1 mM EGTA 10 mM MOPS-KOH, pH 7.2) containing 2 mM PMSF and centrifuged (1,000 x g, 5 min, 4°C). Afterward, the cell pellet was resuspended in hypotonic buffer (100 mM sucrose, 1 mM EGTA 10 mM MOPS-KOH, pH 7.2) containing BSA (2 mg/ml) and 2 mM PMSF and incubated on ice for 5-7min. The cell suspension was homogenized 15

times using a Dounce glass homogenizer (Sartorius, Catalog no. BBI-8540705). Cold hypertonic buffer (1.25 M sucrose, 10 mM MOPS-KOH, pH 7.2) was added to the cell homogenate suspension (1.1 mL/g cells), and the volume was doubled with isotonic buffer containing BSA (2 mg/mL) and PMSF (2 mM). Then the homogenate suspension was centrifuged (1,000 x g, 10 min, 4°C). The supernatant containing mitochondria was centrifuged again to pellet the debris. Protein content in the supernatant was assessed by Bradford assay. Proteins were recovered using pyrogallol red precipitation. The precipitated pellet was solubilized in 2x Laemmli sample buffer with 50 mM DTT.

### siRNA mediated knock-down

The siRNA duplexes from key resources table were used to knock-down the protein of interest. Mission siRNA universal negative control (Sigma, Catalog no.SIC001) was used as a negative control. Cells were reverse-transfected with 25nM of specific siRNA duplex using Lipofectamine RNAiMAX Transfection Reagent (Thermo Fisher Scientific, cat. no. 13778150) diluted in Opti-MEM I Reduced Serum Medium (Thermo Fisher Scientific, cat. no. 11058021) according to the manufacturer's instructions. Cells were collected for further analysis after 48h or 72h depending on the experiment.

### FLAG tag affinity purification

Mitochondria were isolated from cells, in which expression of FLAG-tagged proteins was obtained either by transient transfection ($OCIAD1_{FLAG}$) or by induction with 1 mg/mL tetracycline for 24h ($TIMM21_{FLAG}$, $TIMM23_{FLAG}$). Mitochondria were solubilized with digitonin buffer (1% digitonin %, 150 mM NaCl, 10% glycerol, 1 mM EDTA, 2 mM PMSF, 50 mM Tris-HCl, pH 7.4) to a final concentration of 1 μL of digitonin buffer per 1 μg of mitochondria. The solubilized mitochondria were incubated on ice for 20 min and centrifuged (20,000 x *g*, 15 min, 4°C). The supernatant fraction was collected and incubated with anti-FLAG M2 affinity gel (Sigma, Catalog no. A2220) for 2 h at 4°C. Finally, the anti-FLAG M2 affinity gel beads were pelleted by centrifugation (200 x g, 2 min, 4°C) and the supernatant was discarded. The anti-FLAG M2 affinity gel beads were suspended with wash buffer (150 mM NaCl, 10% glycerol, 1 mM EDTA, 2 mM PMSF, 50 mM Tris-HCl, pH 7.4) containing 0.3% digitonin, and added to a Mobicol spin column (MoBiTec). The anti-FLAG M2 affinity gel beads were washed five times by centrifugation (200 x g, 2 min, 4°C). For western blot analysis the anti-FLAG M2 affinity gel beads were resuspended with 2x Laemmli sample buffer with 50 mM DTT and denatured at 65°C for 5 min. After denaturation, the suspension was centrifuged at 20,000 x g for 30 s at RT. The supernatant containing the eluate was collected and denatured at 65°C for 15 min. The load fraction was mixed with an equal volume of Laemmli sample buffer and denatured at 65°C for 15 min. Samples were subjected to SDS-PAGE and western blot analysis. For mass spectrometry analysis the anti-FLAG M2 affinity gel beads were eluted with 5 mg/mL FLAG Peptide (Sigma, Catalog no. F3290) in wash solution (150 mM NaCl, 10 mM Tris-HCl, pH 7.4) for 2 h at 4°C with mild mixing.

### HA-tag affinity purification

Mitochondria were isolated from cells, in which expression of HA-tagged proteins (HA-TIMM17A, HA-TIMM17B, Prohibitin 2 (PHB2)-HA) was obtained by their transient transfection. Mitochondrial pellets were then resuspended in digitonin-containing (3:1 (w/w) ratio of detergent to protein) Buffer B (0.22 M mannitol, 0.07 M sucrose, 0.02 M HEPES–KOH, pH 7.6, 1 mM EDTA, 1 mM phenylmethylsulfonyl fluoride (PMSF)). Mitochondria were solubilized for 30 min at 4°C and afterward non-solubilized material was removed via centrifugation (13,000 × g, 10 min, 4°C). The load fraction (3%) was taken at this step. The supernatant was incubated with Pierce Anti-HA Agarose (Thermo Fisher Scientific, Catalog no. 26182) beads for 1 h at 4°C. Prior to protein binding, the HA beads were blocked with 3% BSA for 1 h at 4°C to minimize nonspecific interactions. After the incubation, the samples were centrifuged at 500 × g for 1 min at 4°C. The unbound fraction (3%) was taken at this step. The HA beads with bound target proteins were then washed 3 times with Buffer B. The elution step was performed by incubating protein-bound beads with 2x sample buffer (without a reducing agent) for 10 min at 95°C with shaking. The samples were centrifuged at 13 000 rpm for 1 min. The eluted proteins were then transferred to new Eppendorf tubes and 2-Mercaptoethanol was added to a final concentration of 5%. The samples were boiled again at 95°C for 5 min. All the fractions were analyzed using SDS-PAGE followed by western blotting.

### *In organello* import assay

The cDNA of precursor proteins was cloned into a pTNT vector under the SP6 promoter. The recombinant plasmid was used to synthesize $^{35}S$ radiolabeled precursors in TNT SP6 Quick Coupled Transcription/Translation system (Promega, Catalog no. L2080) according to the manufacturer's protocol. Freshly isolated mitochondria were mixed with import buffer (250 mM sucrose, 80 mM potassium acetate, 5 mM magnesium acetate, 5 mM methionine, 10 mM sodium succinate, 20 mM HEPES-KOH, pH 7.4) supplemented with fresh 5 mM ATP and incubated for 2 min at 24°C. After incubation, the import was initiated by adding radiolabeled precursor to import buffer containing mitochondria and aliquoted at specified time points. A combination of 0.1 mM valinomycin, 1 mM oligomycin and 0.8 mM antimycin A or carbonyl cyanide-*p*-trifluoromethoxyphenylhydrazone (FCCP) was used to treat mitochondria before adding radioactive precursors to block mitochondrial inner membrane potential, which affects import efficiency. After import, proteinase K was administered for 10 min at 4°C to degrade non-imported precursors. Then, PMSF (2 mM) was used to inactivate proteinase K. Next, the samples were centrifuged (20,000 x *g*, 10 min, 4°C). The mitochondrial pellet was centrifuged again after being rinsed with high sucrose solution (500 mM sucrose, 20 mM HEPES-KOH, pH 7.4) containing 2 mM PMSF. Finally, the mitochondrial pellet containing imported radioactive precursor proteins was solubilized in 2x Laemmli sample buffer (containing 2 mM PMSF and

50 mM DTT) or digitonin-containing buffer (1% digitonin, 0.1 mM EDTA, 50 mM NaCl, 10% (w/v) glycerol, 1 mM PMSF, 20 mM Tris-HCl, pH 7.4) for BN-PAGE. Samples were analyzed by reducing SDS-PAGE and autoradiography.

## Blue Native-PAGE

Mitochondrial pellets were solubilized in digitonin-containing buffer (1% digitonin, 0.1 mM EDTA, 50 mM NaCl, 10% (w/v) glycerol, 1 mM PMSF, 20 mM Tris-HCl, pH 7.4) to a final concentration of 1 μg/μL for 30 min at 4°C. After clearing the lysates by centrifugation at 14,000 x *g* for 10 min at 4°C, loading dye was added (5% Coomassie brilliant blue G-250, 500 mM 6-aminohexanoic acid, and 100 mM Bis-Tris, pH 7.0). Mitochondrial complexes were separated by resolving samples in 3–13%, 4–16%, 4–13% or 4–10% polyacrylamide gradient gels and analyzed by western blotting or autoradiography. The High Molecular Weight Calibration Kit for native electrophoresis (Cytvia, Catalog no. GE17-0445-01) was used as a molecular weight standard.

## Quantitative real-time PCR

Total RNA was isolated by RNeasy Plus Mini Kit (Qiagen, cat. no. 74134) according to the manufacturer's instructions. 1 μg total RNA was used to generate cDNA using SuperScript IV First-Strand Synthesis System (Thermo Fisher Scientific, cat. no. 18091050). From total RNA, cDNA was synthesized using Maxima First Strand cDNA Synthesis Kit for RT-qPCR with dsDNase (Thermo Fisher Scientific, cat. no. K1671). RT-qPCR was performed using SensiFAST SYBR Hi-ROX Kit (Bioline, cat. no. BIO-92020) in a 96-well white plate (Roche, cat. no. 4729692001) using a LightCycler480 (Roche). Fold changes in the mRNA expression level of the target genes were calculated using the ΔΔCt method. The expression levels of actin were used as internal standards.

## Mitochondrial membrane electrochemical potential measurement

Cells were treated with DMSO, CCCP (10 μM), and oligomycin (0.6 μM), respectively, for 30 min before they were collected by trypsinization and then incubated in a medium containing 15 nM TMRM (Thermo Fisher Scientific, Catalog no. M20036) at 37°C for 15 min. Cells were pelleted by centrifugation (1,000 x *g*, 5 min, RT), resuspended and immediately measured in Attune Nxt acoustic focusing cytometer (Life Technologies).

## Sample preparation for live-cell STED microscopy

HeLa cells were cultured in Dulbecco's modified Eagle's medium (DMEM) with glutaMAX and 4.5 g/L glucose (Thermo Fisher Scientific, Waltham, MA, USA) at 37°C and 5% $CO_2$. The culture medium was supplemented with 100 U/ml penicillin and 100 μg/mL streptomycin (Merck Millipore, Burlington, MA, USA), 1 mM sodium pyruvate (Sigma-Aldrich, Munich, Germany), and 10% (v/v) fetal bovine serum (Merck Millipore). Cells were seeded in 3.5 cm glass bottom dishes (Ibidi GmbH, Gräfelfing, Germany) and cultivated over night at 37°C and 5% $CO_2$. The cells were stained with 200 nM PK Mito Orange and 1 μM SNAP-cell 647-SiR (NEB) as described previously.[25]

## STED microscopy

Stimulated emission depletion (STED) nanoscopy was performed using a dual-color Expert Line STED microscope (Abberior Instruments, Göttingen, Germany) equipped with a 775 nm STED-Laser and an UPlanSApo 100×/1.40 Oil [infinity]/0.17/FN26.5 objective (Olympus, Tokyo, Japan). Alexa Fluor 594 and PK Mito Orange were excited at 561 nm. Abberior STAR RED was excited at 640 nm. STED was performed at 775 nm. Images were recorded with a pixel size of 20–30 nm in the 2D STED mode. Raw data were smoothed using a low-pass filter (fixed sample data) or deconvolved (live-cell data) in the Imspector Software (Version 0.14.11616, Abberior Instruments). Live-cell STED microscopy recordings were taken at room temperature in HEPES-buffered DMEM.

## Sample preparation for fluorescence microscopy

Cells were chemically fixed using a pre-warmed (37°C) solution of 8% formaldehyde in PBS (137 mM NaCl, 10 mM $Na_2HPO_4$, 2.68 mM KCl, pH 7.4) for 10 min. Following fixation, cells were extracted using 0.5% (v/v) Triton X-100 detergent in PBS. Samples were washed with PBS and blocked with 5% (w/v) BSA in PBS for 20 min. For immunolabeling, samples were incubated with primary antibodies against FLAG tag (Sigma Aldrich, cat. no. F3165; Thermo Fisher Scientific, cat. no. PA1-984B), TOMM20 (Santa Cruz Biotechnology, Dallas, TX, USA, cat. no. sc-11415), ATPB (Abcam, Cambridge, UK, cat. no. ab5432) and MIC60 (Abcam, cat. no. ab137057) diluted in 5% (w/v) BSA in PBS for 1.5 h at room temperature. Samples were washed 5 times in PBS to remove unbound antibodies. Afterward, primary antibodies were detected with secondary goat anti-rabbit or sheep anti-mouse antibodies labeled with either Alexa Fluor 594 (Thermo Fisher Scientific) or custom-labeled with Abberior STAR RED (dye from Abberior, Goettingen, Germany; antibody from Jackson Immuno Research Laboratories, West Grove, PA, USA). Secondary antibodies were diluted in 5% (w/v) BSA in PBS and incubated for 1 h at room temperature. After washing thoroughly with PBS, the cells were mounted in Mowiol supplemented with 0.1%, 1,4-Diazabicyclo[2.2.2]octane (DABCO) and 2.5 μg/mL 4′,6-Diamidin-2-phenylindol (DAPI) (Sigma-Aldrich).

## Mass spectrometry

### *Preparation of affinity purification samples for proteomic analysis*

Affinity-purified elute fractions were mixed with 0.1 M Tris-HCl pH 8.0 containing 10 mM chloroacetamide and 5 mM TCEP. Proteins were then digested overnight with sequencing grade modified trypsin (Promega) at 37°C, centrifuged (300 x g, 10 min, RT), and then trypsin was inactivated by 1% TFA. Desalting and TMT labeling were performed on stage tips.[48] Stage tips were packed with three punches of AttractSPE Discs Bio C18 (Affinisep) with a 16-gauge blunt end needle. The resin was conditioned with methanol, followed by 50% acetonitrile in 0.1% formic acid (FA), and twice equilibrated with 150 μL of 0.1% FA. The digested peptides were loaded and washed twice with 150 μL of 0.1% FA. 80 μg of a TMT10plex Isobaric Label Reagent (Thermo Fisher Scientific, Catalog no. 90110) was dissolved in 2 μL of acetonitrile and further diluted in 200 μL of 50 mM HEPES, pH 8. The solution was passed through the C18-adsorbed peptides and washed with 150 μL of 0.1% FA (3×) and eluted with 60 μL of 60% ACN in 0.1% FA. Samples labeled with isobaric TMT reagents were pooled into a TMT6plex sample, and the solvent was removed by SpeedVac Concentrator. TMT-labeled peptide mixtures were dissolved in 2% of acetonitrile in 0.1% TFA before LC-MS/MS measurement. Analysis of affinity purification by isobaric TMT labeling may report a lower apparent magnitude of enrichment as compared to western blot analysis due to a different method of elution (FLAG peptide vs. Laemmli buffer) and to peptide co-fragmentation resulting in ratio compression compared to western blot results.

### *LC-MS/MS measurements of affinity purification samples*

Chromatographic separation was performed using an Easy-Spray Acclaim PepMap column (50 cm long × 75 μm inner diameter, Thermo Fisher Scientific) at 45°C by applying 160 min acetonitrile gradients in 0.1% aqueous FA at a flow rate of 300 nL/min. An UltiMate 3000 nano-LC system was coupled to a Q Exactive HF-X mass spectrometer via an easy-spray source (Thermo Fisher Scientific). The Q Exactive HF-X was operated in data-dependent mode with survey scans acquiring at a resolution of 60,000 at m/z 200. Up to 15 of the most abundant isotope patterns with charges 2–6 from the survey scan were selected with an isolation window of 0.7 m/z and fragmented by higher-energy collision dissociation (HCD) with normalized collision energies of 32. At the same time, the dynamic exclusion was set to 30 s. The maximum ion injection times for the survey and MS/MS scans (acquired with a resolution of 45,000 at m/z 200) were 20 and 96 ms, respectively. The ion target value for MS was set to 3e6 and for MS/MS to 1e4, and the intensity threshold for MS/MS was set to 1.0e5.

### *Mass spectrometry data processing*

The data were processed with MaxQuant v. 1.6.7.0, and the peptides were identified from the MS/MS spectra searched against the UniProt KB Human Proteome (downloaded on 29.05.2019) using the built-in Andromeda search engine. Cysteine carbamidomethylation was set as a fixed modification, and methionine oxidation and protein N-terminal acetylation were set as variable modifications. Reporter ion MS2 was selected for quantification (TMT), and the minimal precursor intensity fraction was set to 0.75. For *in silico* digests of the reference proteome, cleavages of arginine or lysine followed by any amino acid were allowed (trypsin/P), and up to two missed cleavages were allowed. The FDR was set to 0.01 for peptides, proteins, and sites. Second peptide search was disabled. Other parameters were used as pre-set in the software. Unique and razor peptides were used for quantification helping protein grouping (razor peptides are the peptides uniquely assigned to protein groups and not to individual proteins). Reporter intensity values for protein groups were loaded into Perseus v. 1.6.6.0.[49] Standard filtering steps were applied to clean up the dataset: reverse (matched to decoy database), only identified by site, and potential contaminant (from a list of commonly occurring contaminants included in MaxQuant) protein groups were removed. Reporter intensity values were Log2 transformed. For each sample, the median reporter intensity was subtracted. Thresholds of protein enrichment of Log2 > 0.5 and *p* value –LogP >2 were used to return protein levels that were statistically significantly more abundant in $OCIAD_{FLAG}$ vs. empty vector. This dataset has been deposited to the ProteomeXchange Consortium[44] via the PRIDE[45] partner repository with the dataset identifier PRIDE: PXD043863.

## QUANTIFICATION AND STATISTICAL ANALYSIS

Experiments were performed in at least 3 independent biological replicates (*n* = 3). Densitometry of western blot and autoradiography images was performed using the ImageJ software. Statistical analyses were performed using GraphPad Prism v8.0. Data was analyzed using unpaired two-tailed Student's t test except mass spectrometry data, which was analyzed using one-tailed Student's t test. Data is presented as mean ± standard error of the mean unless stated otherwise. Sample size, error bars, *p*-values, and statistical methods are described in figure legends. A *p*-value less than 0.05 was considered to be significant.

Cell Reports, Volume *43*

## Supplemental information

# OCIAD1 and prohibitins regulate the stability of the TIM23 protein translocase

**Praveenraj Elancheliyan, Klaudia K. Maruszczak, Remigiusz Adam Serwa, Till Stephan, Ahmet Sadik Gulgec, Mayra A. Borrero-Landazabal, Sonia Ngati, Aleksandra Gosk, Stefan Jakobs, Michal Wasilewski, and Agnieszka Chacinska**

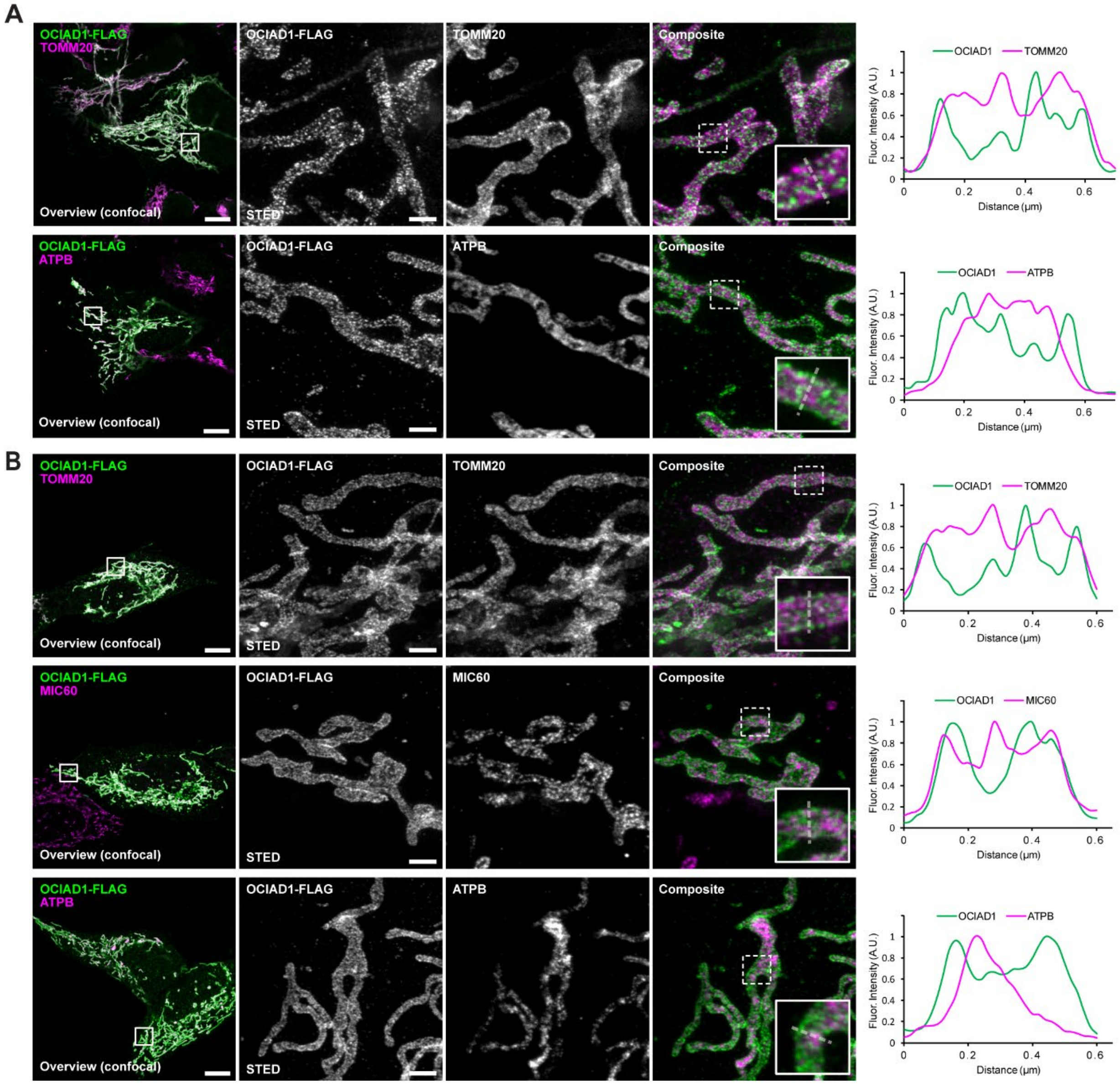


**Figure S1. Submitochondrial distribution of OCIAD1-FLAG, related to Figure 1** (A, B) OCIAD1-FLAG submitochondrial localization, visualized by confocal and 2D STED microscopy. Cells were transfected with a plasmid coding OCIAD1-FLAG, fixed and immunolabeled against the FLAG-epitope and different mitochondrial proteins. Samples were recorded by 2-color 2D STED nanoscopy. Fluorescence intensity line profiles were measured along the dashed lines shown in the insets. Fluorescence intensities were normalized and plotted across the mitochondrial tubules. Line profiles indicate the intramitochondrial position of the individual protein clusters. (A) HEK 293 cells were labeled against the FLAG epitope and TOMM20 (outer membrane) or ATPB (cristae membrane), respectively. (B) U-2 OS cells were labeled against the FLAG epitope and

TOMM20 (outer membrane), MIC60 (inner boundary membrane/crista junctions), or ATPB (cristae membrane), respectively. ATPB signals are generally shifted towards the center of the mitochondrial tubules. Scale bars: 10 µm (overview), 1 µm (STED).

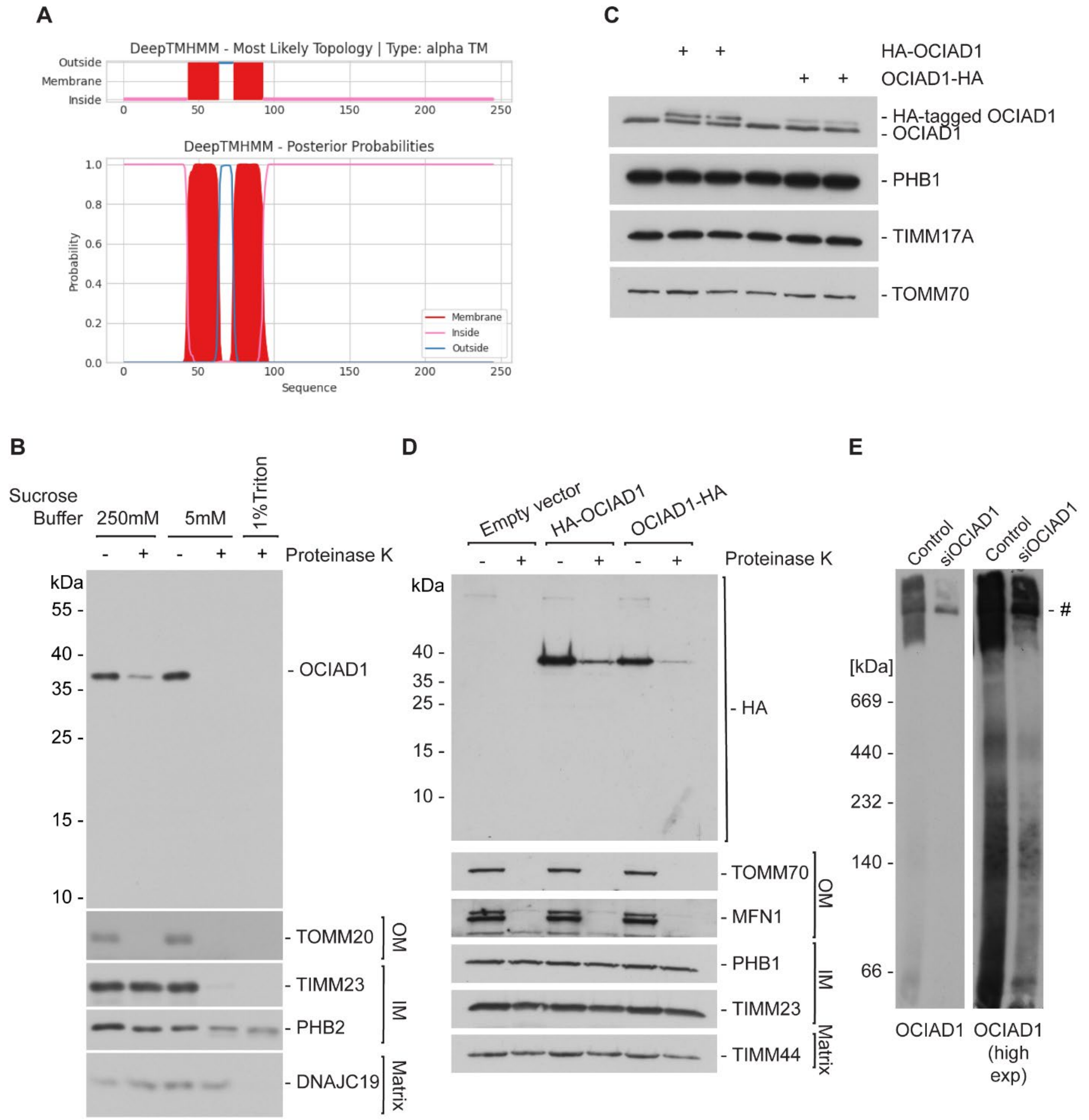


**Figure S2. OCIAD1 is a transmembrane protein of the outer mitochondrial membrane and exposes both the N- and C-terminus to the cytosol, related to Figure 1** (A) DeepTMHMM prediction of OCIAD1 transmembrane domains. (B) Localization of mitochondrial proteins, analyzed by limited degradation by proteinase K in intact mitochondria (250 mM sucrose), mitoplasts (5 mM sucrose), and mitochondrial lysates (1% Triton X-100). The samples were analyzed by SDS-PAGE and Western blot. OM, outer membrane; IM, inner membrane. (C) Expression of HA-OCIAD1 and OCIAD1-HA in HEK293T cells. The samples were analyzed by

SDS-PAGE and Western blot. (D) Localization of mitochondrial proteins isolated from HEK293T cells expressing HA-OCIAD1 and OCIAD1-HA, analyzed by limited degradation by proteinase K in intact mitochondria. The samples were analyzed by SDS-PAGE and Western blot. OM, outer membrane; IM, inner membrane. (E) Analysis of OCIAD1 in mitochondria isolated HEK293 cells transfected with scramble (Control) or siRNA against OCIAD1 (siOCIAD1). The samples were analyzed by BN-PAGE and Western blot. #, unspecific band.

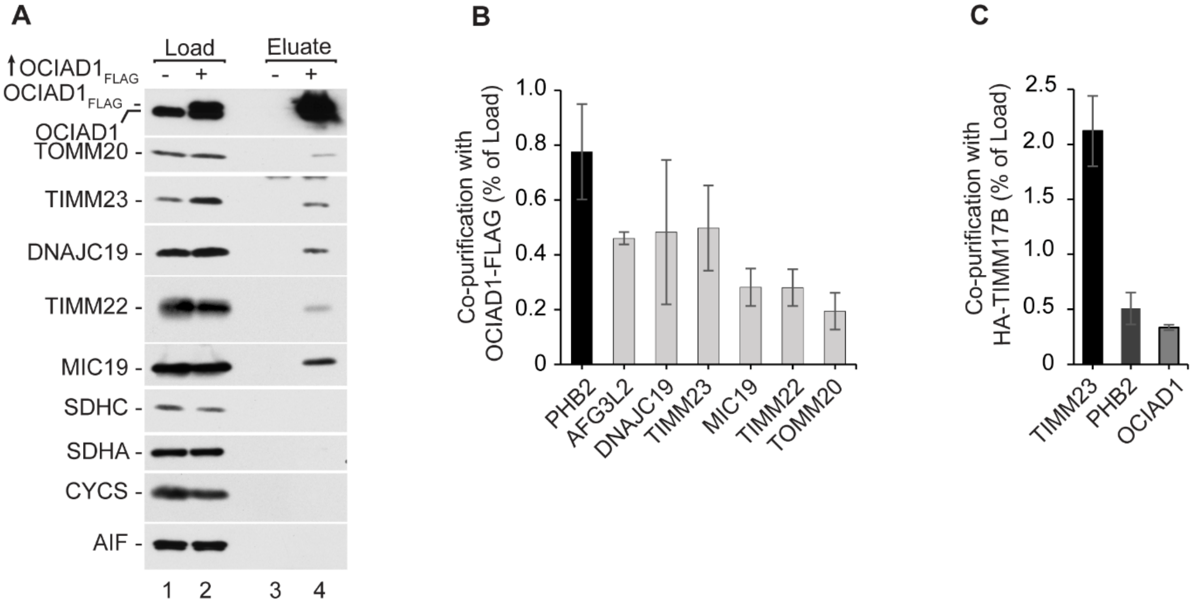


**Figure S3. OCIAD1 interacts with the prohibitin complex and the TIM23 complex, related to Figure 2** (A) Affinity purification of OCIAD1$_{FLAG}$. Mitochondria were isolated from HEK293 cells that expressed OCIAD1$_{FLAG}$ or an empty vector, solubilized in digitonin-containing buffer, and subjected to immunoprecipitation with FLAG affinity resin. Samples were analyzed by SDS-PAGE and Western blot. Load: 2 %; Eluate: 100 %. (B) Quantification of protein co-purification with OCIAD1$_{FLAG}$ presented as a ratio of eluate to load (means ± SEM, n = 3). (C) Quantification of protein co-purification with HA-TIMM17B presented as a ratio of eluate to load (means ± SEM, n = 3).

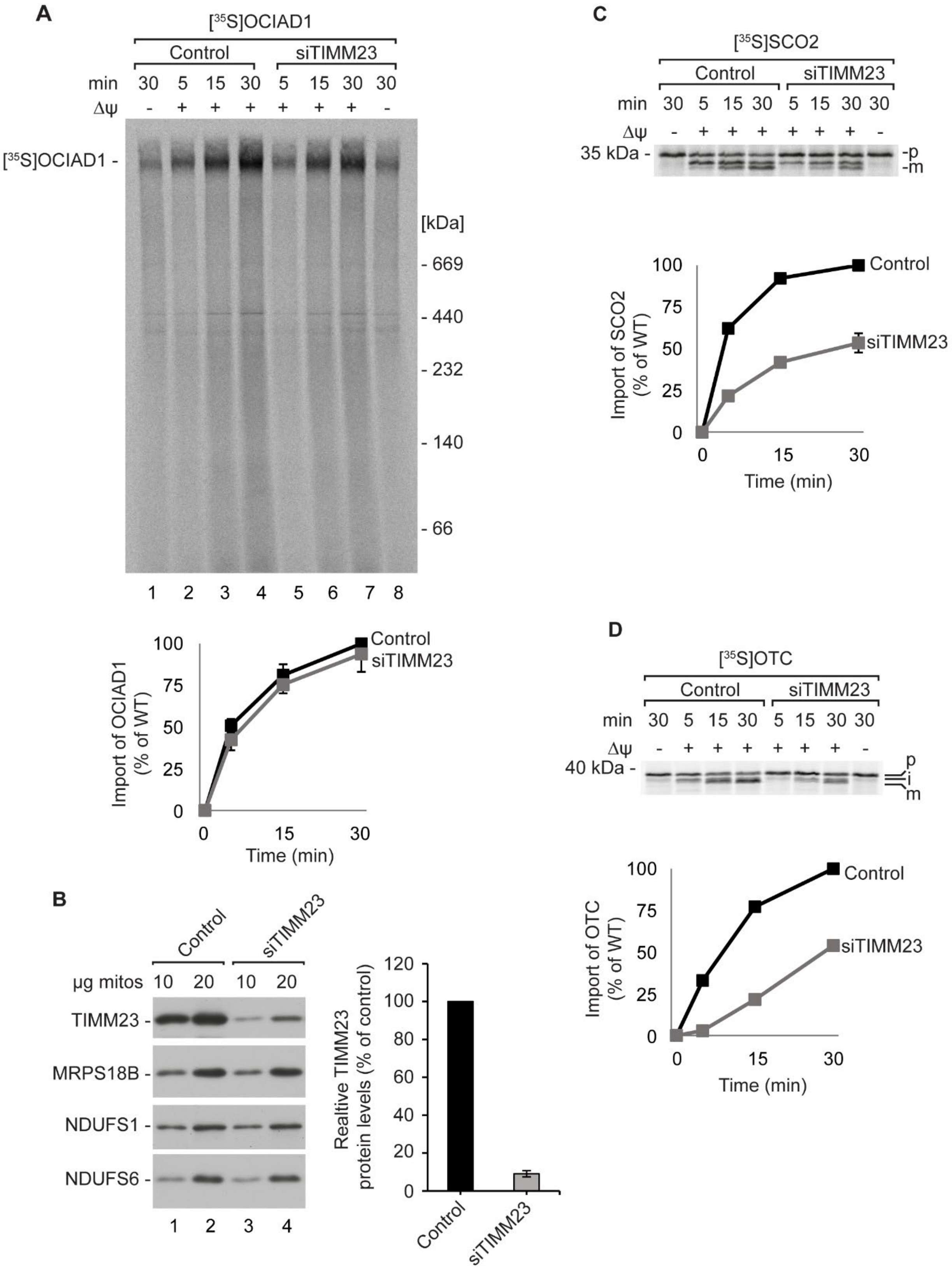

A
[35S]OCIAD1
Control
siTIMM23
min 30 5 15 30 5 15 30 30
Δψ - + + + + + + -
[35S]OCIAD1 -
[kDa]
- 669
- 440
- 232
- 140
- 66
1 2 3 4 5 6 7 8
Import of OCIAD1 (% of WT)
100
75
50
25
0
0 15 30
Time (min)
Control
siTIMM23
B
Control
siTIMM23
µg mitos 10 20 10 20
TIMM23 -
MRPS18B -
NDUFS1 -
NDUFS6 -
1 2 3 4
Realtive TIMM23 protein levels (% of control)
120
100
80
60
40
20
0
Control
siTIMM23
C
[35S]SCO2
Control
siTIMM23
min 30 5 15 30 5 15 30 30
Δψ - + + + + + + -
35 kDa -
-p
-m
Import of SCO2 (% of WT)
100
75
50
25
0
0 15 30
Time (min)
Control
siTIMM23
D
[35S]OTC
Control
siTIMM23
min 30 5 15 30 5 15 30 30
Δψ - + + + + + + -
40 kDa -
p
i
m
Import of OTC (% of WT)
100
75
50
25
0
0 15 30
Time (min)
Control
siTIMM23

**Figure S4. OCIAD1 is not imported by the TIM23 pathway, related to Figure 2** (A) *In organello* import of OCIAD1. Radiolabeled $^{[35S]}$OCIAD1 precursors were imported into the mitochondria of siRNA TIMM23-depleted and control HEK293 cells. Mitochondria were analyzed with 3-13 % BN-PAGE gels and autoradiography. Control cells were transfected with Mission siRNA universal negative control. Where indicated the mitochondrial membrane electrochemical potential (ΔΨ) was dissipated by valinomycin, antimycin A, and oligomycin. The quantification of autoradiography signals is presented as the mean ± SEM ($n$ = 3). The amount of imported protein into control mitochondria after 30 min was set to 100 %. (B) Western blot analysis of mitochondria isolated from control and TIMM23-depleted cells. The quantification of TIMM23 from lanes that were loaded with 10 μg mitochondria is presented as the mean ± SEM ($n$ = 3). Control, cells transfected with Mission siRNA universal negative control. (C, D) *In organello* import of OCIAD1. Radiolabeled $^{[35S]}$SCO2 (C) or $^{[35S]}$OTC (D) were imported into the mitochondria of TIMM23-depleted and control HEK293 cells. Mitochondria were analyzed with SDS-PAGE gels and autoradiography. Control cells were transfected with Mission siRNA universal negative control. Where indicated the mitochondrial membrane electrochemical potential (ΔΨ) was dissipated by valinomycin, antimycin A, and oligomycin. The quantification of autoradiography signals is presented as the mean ± SEM ($n$ = 3). The amount of imported protein into control mitochondria after 30 min was set to 100 %. p, precursor form; i, intermediate form; m, mature form.

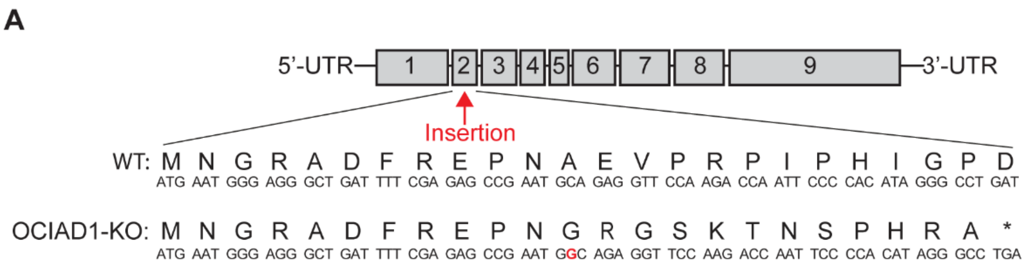


**Figure S5. Schematic representation of mutation of the *OCIAD1* gene in OCIAD1-KO cells, related to Figure 3** (A) The OCIAD1-KO cell line was generated using CRISPR/Cas9 technology by the homozygous insertion of guanosine (G) in the second intron of *OCIAD1*, leading to a frameshift in the ORF and a premature stop codon.

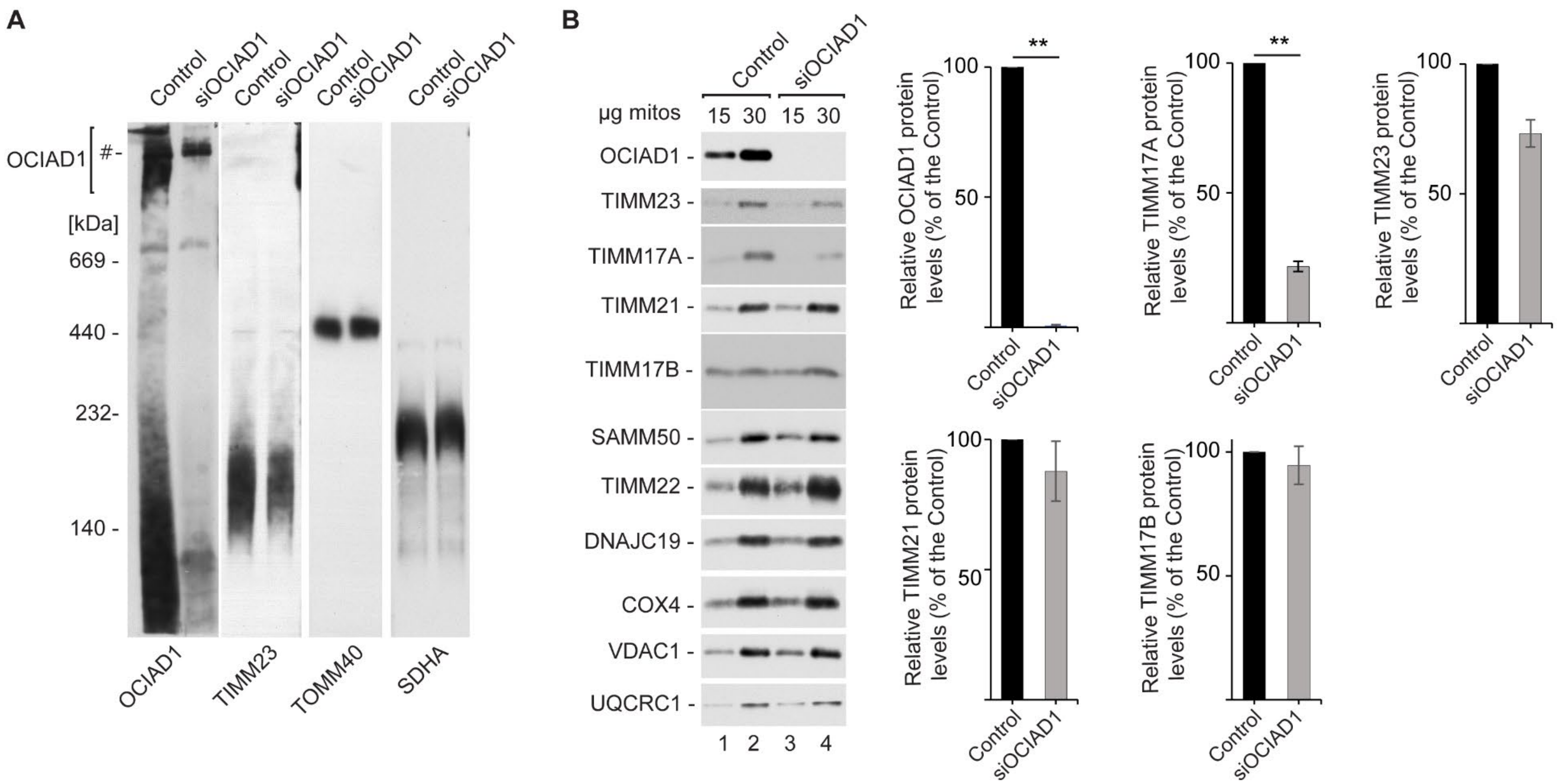
A
Control
siOCIAD1
OCIAD1
#-
[kDa]
669 -
440 -
232-
140 -
OCIAD1
TIMM23
TOMM40
SDHA
B
µg mitos
15 30 15 30
OCIAD1 -
TIMM23 -
TIMM17A -
TIMM21 -
TIMM17B -
SAMM50 -
TIMM22 -
DNAJC19 -
COX4 -
VDAC1 -
UQCRC1 -
1 2 3 4
Relative OCIAD1 protein levels (% of the Control)
Relative TIMM17A protein levels (% of the Control)
Relative TIMM23 protein levels (% of the Control)
Relative TIMM21 protein levels (% of the Control)
Relative TIMM17B protein levels (% of the Control)
100
50
**

**Figure S6. OCIAD1 depletion downregulates the TIM23 complex, related to Figure 5** (A) Protein complexes in mitochondria that were isolated from HEK293 cells with native or siRNA-depleted OCIAD1, solubilized with 1% digitonin-containing buffer, and resolved in 4-13 % BN-PAGE gels, followed by Western blot. Control, cells transfected with Mission siRNA universal negative control; #, unspecific band. (B) Western blot analysis of mitochondria that were isolated from HEK293 cells with native and siRNA-depleted OCIAD1. The quantification of protein signals from lanes that were loaded with 30 µg mitochondria is presented as the mean ± SEM ($n$ = 3). *$p < 0.05$, **$p < 0.01$ (two-tailed Student's $t$-test). mitos, mitochondria

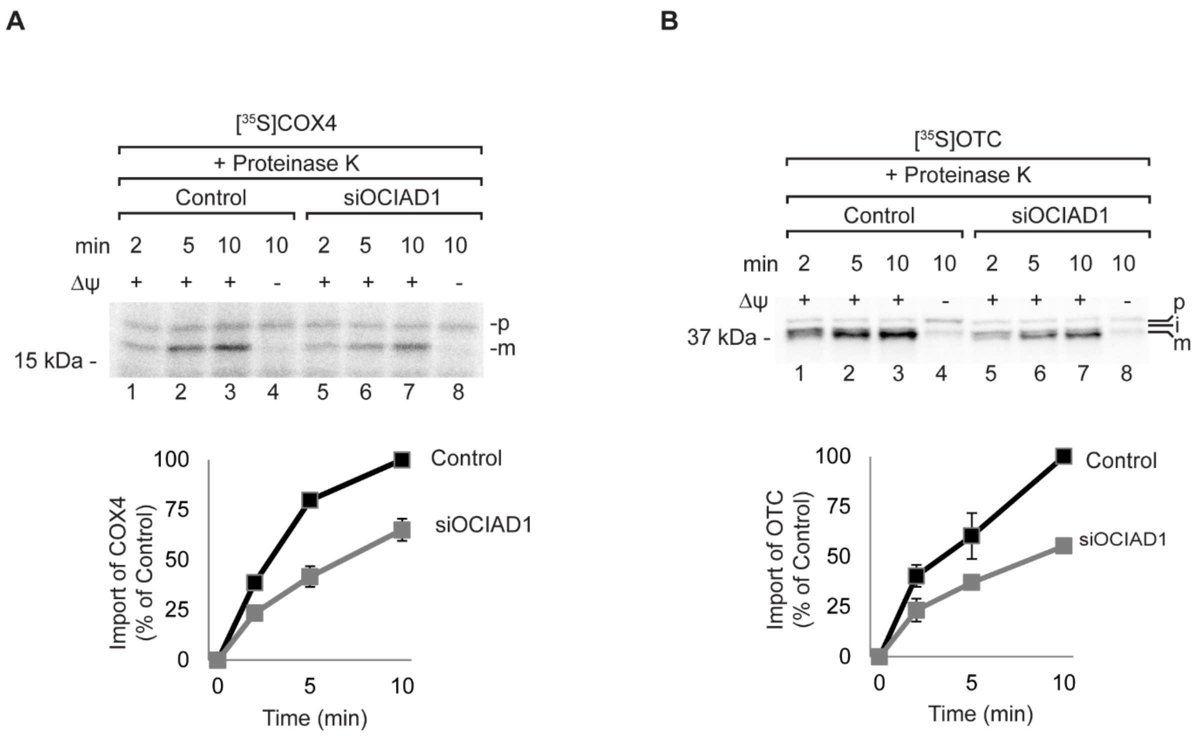


**Figure S7. The absence of OCIAD1 downregulates the efficiency of the TIM23 import pathway, related to Figure 5** (A, B) *In organello* import of [$^{35}$S]COX4 (A) and [$^{35}$S]OTC (B) precursors into mitochondria that were isolated from HEK293 cells with native or siRNA-depleted OCIAD1, analyzed by SDS-PAGE and autoradiography. The quantification of autoradiography signals is presented as the mean ± SEM ($n$ = 3). The amount of imported protein into wild-type mitochondria after 10 min was set to 100 %. Control, cells transfected with Mission siRNA universal negative control. VOA, valinomycin, antimycin A, and oligomycin. p, precursor form; i, intermediate form; m, mature form.

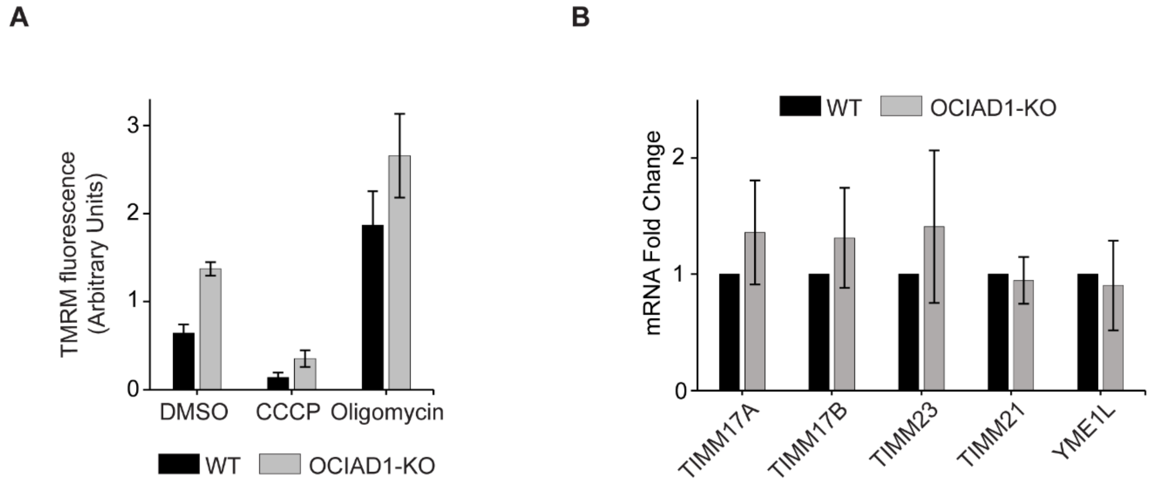


**Figure S8. The absence of OCIAD1 does not influence the mitochondrial membrane electrochemical potential or mRNA levels of TIM23 subunits, related to Figure 5** (A) Mitochondrial membrane electrochemical potential in wild-type and OCIAD1-KO HEK293 cells treated with CCCP (10 µM) or oligomycin (0.6 µM) for 30 min, analyzed by TMRM staining, followed by flow cytometry. The quantification of TMRM fluorescence is presented as the mean ± SEM (*n* = 3). CCCP, carbonyl cyanide m-chlorophenyl hydrazone; TMRM, tetramethylrhodamine methyl ester. (B) mRNA expression patterns of selected transcripts, analyzed by RT-qPCR in wild-type and OCIAD1-KO cells. mRNA levels are presented as fold changes relative to wild-type HEK293 cells (mean ± SD, *n* = 3). WT, wild-type.

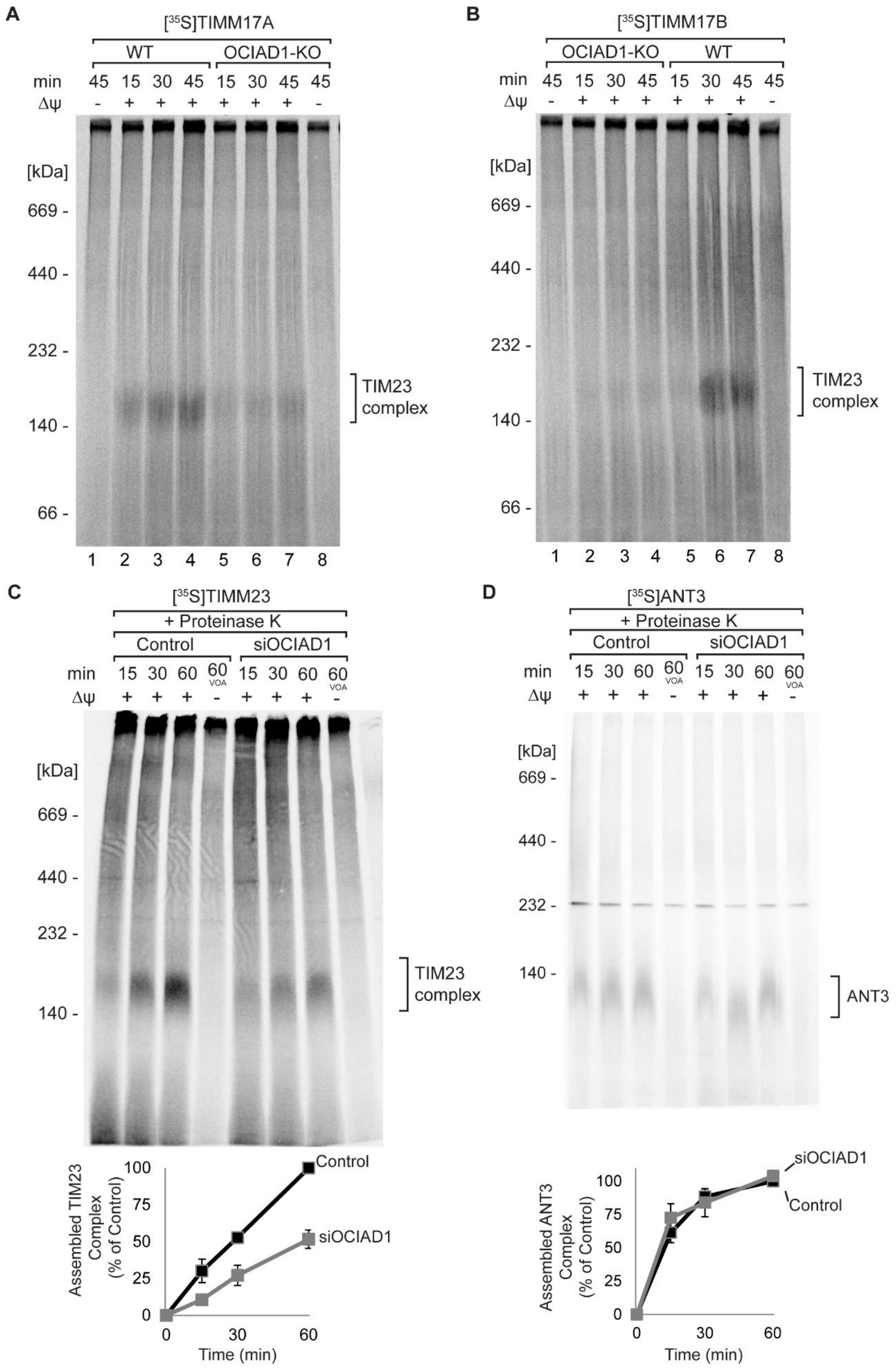

A
[35S]TIMM17A
WT
OCIAD1-KO
min 45 15 30 45 15 30 45 45
Δψ - + + + + + + -
[kDa]
669
440
232
140
66
TIM23 complex
1 2 3 4 5 6 7 8
B
[35S]TIMM17B
OCIAD1-KO
WT
min 45 15 30 45 15 30 45 45
Δψ - + + + + + + -
[kDa]
669
440
232
140
66
TIM23 complex
1 2 3 4 5 6 7 8
C
[35S]TIMM23
+ Proteinase K
Control
siOCIAD1
min 15 30 60 60 VOA 15 30 60 60 VOA
Δψ + + + - + + + -
[kDa]
669
440
232
140
TIM23 complex
Assembled TIM23 Complex (% of Control)
Time (min)
Control
siOCIAD1
D
[35S]ANT3
+ Proteinase K
Control
siOCIAD1
min 15 30 60 60 VOA 15 30 60 60 VOA
Δψ + + + - + + + -
[kDa]
669
440
232
140
ANT3
Assembled ANT3 Complex (% of Control)
Time (min)
siOCIAD1
Control

**Figure S9. OCIAD1 depletion decreases the assembly of the TIM23 complex, related to Figure 6** (A, D) *In organello* import of $^{[35S]}$TIMM17A (A) and $^{[35S]}$TIMM17B (B) precursors into mitochondria that were isolated from the wild-type (WT) or OCIAD1-KO HEK293 cells, analyzed by 3-13 % BN-PAGE gels and autoradiography. Where indicated the mitochondrial membrane electrochemical potential ($\Delta\Psi$) was dissipated by VOA, valinomycin, antimycin A, and oligomycin. (C, D) *In organello* import of $^{[35S]}$TIMM23 (A) and $^{[35S]}$ANT3 (B) precursors into mitochondria that were isolated from HEK293 cells with native or siRNA-depleted OCIAD1, analyzed by 4-13 % BN-PAGE gels and autoradiography. Where indicated the mitochondrial membrane electrochemical potential ($\Delta\Psi$) was dissipated by VOA, valinomycin, antimycin A, and oligomycin. The quantification of autoradiography signals is presented as mean ± SEM ($n$ = 3). The amount of imported protein into wild-type mitochondria at 60 min was set to 100 %. Control, cells transfected with Mission siRNA universal negative control.

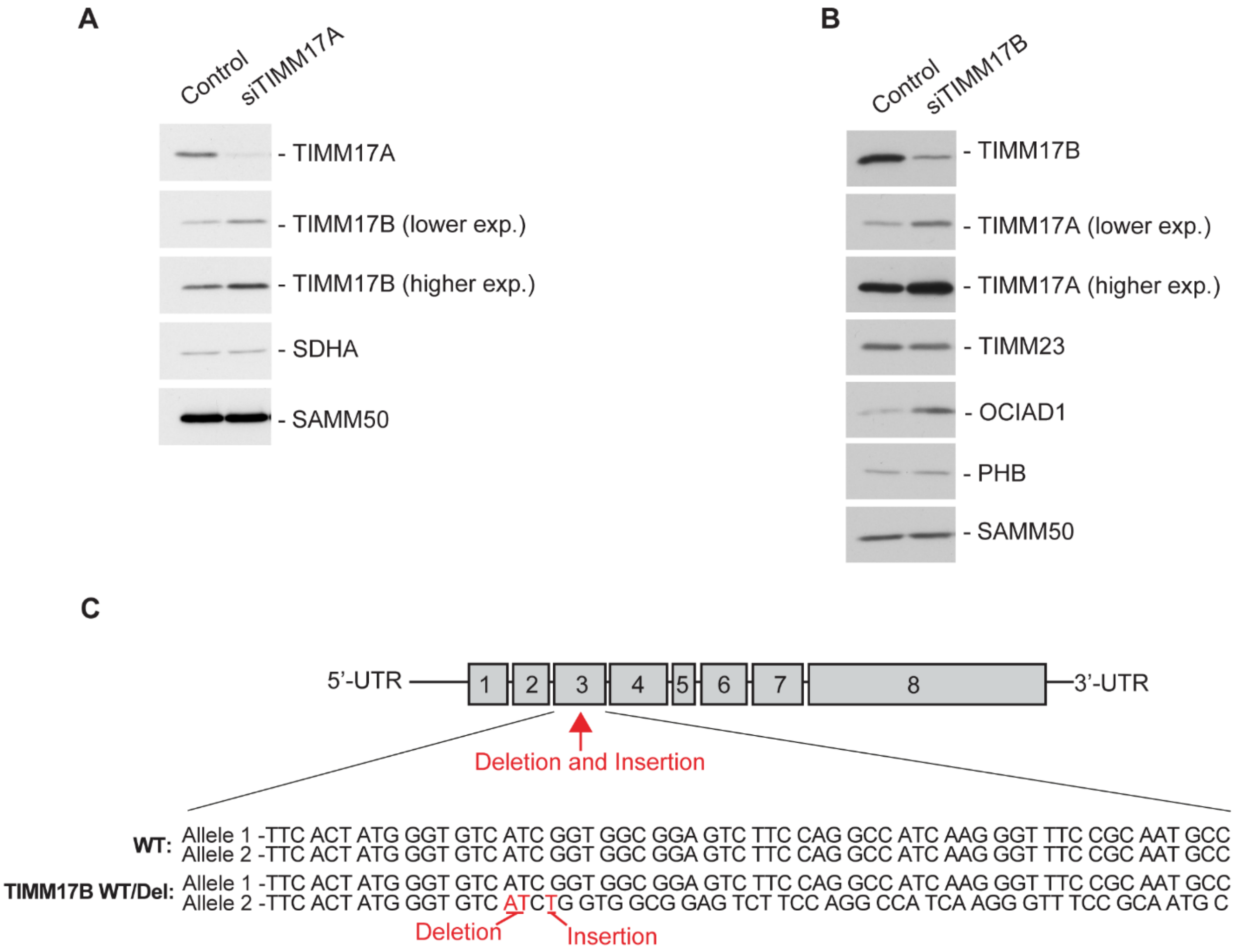


**Figure S10. Compensatory regulation of TIMM17A and TIMM17B proteins. Schematic representation of mutations of the *TIMM17B* gene in TIMM17B WT/Del HEK293 cells, related to Figure 7** (A) Western blot analysis of mitochondria isolated from control and siRNA-depleted TIMM17A cells. Control, cells transfected with Mission siRNA universal negative control. (B) Western blot analysis of mitochondria isolated from control and siRNA-depleted TIMM17B cells. Control, cells transfected with Mission siRNA universal negative control. (C) The TIMM17B WT/Del cell line was generated using CRISPR/Cas9 technology by heterozygous deletion and insertion in the third intron of *TIMM17B*, leading to a frameshift in the ORF and a premature stop codon. WT, wild-type.
